# Marine microbial metagenomes sampled across space and time

**DOI:** 10.1038/sdata.2018.176

**Published:** 2018-09-04

**Authors:** Steven J. Biller, Paul M. Berube, Keven Dooley, Madeline Williams, Brandon M. Satinsky, Thomas Hackl, Shane L. Hogle, Allison Coe, Kristin Bergauer, Heather A. Bouman, Thomas J. Browning, Daniele De Corte, Christel Hassler, Debbie Hulston, Jeremy E. Jacquot, Elizabeth W. Maas, Thomas Reinthaler, Eva Sintes, Taichi Yokokawa, Sallie W. Chisholm

**Affiliations:** 1 Department of Civil and Environmental Engineering, Massachusetts Institute of Technology, Cambridge, MA 02139, USA; 2 Department of Limnology and Bio-Oceanography, University of Vienna, Vienna 1090, Austria; 3 Department of Earth Sciences, University of Oxford, Oxford OX1 3AN, UK; 4 Marine Biogeochemistry Division, GEOMAR Helmholtz Centre for Ocean Research, Kiel 24148, Germany; 5 Research and Development Center for Marine Biosciences, Japan Agency for Marine-Earth Science and Technology, Yokosuka 237-0061, Japan; 6 Department F.-A. Forel for Environmental and Aquatic Sciences, University of Geneva, Geneva 1211, Switzerland; 7 National Institute of Water and Atmospheric Research, Auckland 1010, New Zealand; 8 Department of Biological Sciences, University of Southern California, Los Angeles, CA 90089, USA; 9 Ministry for Primary Industries, Napier 4144, New Zealand; 10 Department of Biology, Massachusetts Institute of Technology, Cambridge, MA 02139, USA

**Keywords:** Water microbiology, Metagenomics, Marine biology, Marine biology

## Abstract

Recent advances in understanding the ecology of marine systems have been greatly facilitated by the growing availability of metagenomic data, which provide information on the identity, diversity and functional potential of the microbial community in a particular place and time. Here we present a dataset comprising over 5 terabases of metagenomic data from 610 samples spanning diverse regions of the Atlantic and Pacific Oceans. One set of metagenomes, collected on GEOTRACES cruises, captures large geographic transects at multiple depths per station. The second set represents two years of time-series data, collected at roughly monthly intervals from 3 depths at two long-term ocean sampling sites, Station ALOHA and BATS. These metagenomes contain genomic information from a diverse range of bacteria, archaea, eukaryotes and viruses. The data’s utility is strengthened by the availability of extensive physical, chemical, and biological measurements associated with each sample. We expect that these metagenomes will facilitate a wide range of comparative studies that seek to illuminate new aspects of marine microbial ecosystems.

## Background & Summary

Microbial communities are key drivers of marine biogeochemistry. Our understanding of the incredible complexity and diversity of natural microbial populations has been greatly enhanced by the advent of cultivation-independent techniques for sequencing DNA directly from an environmental sample. Despite progress in describing the complexity of these natural systems, many gaps remain in our understanding of the distribution of genes and organisms in the oceans as well as the selective forces that structure community composition and distribution across space and time.

While previous large-scale marine sequencing efforts such as the Global Ocean Survey^1,2^ and Tara Oceans^3^ expeditions have greatly expanded our understanding of ocean microbiomes, these ecosystems remain vastly undersampled. The oceans present many challenges for sampling, including both their dynamic nature–e.g. weather, turbulence, movements of water masses, and mixing–as well as their remoteness. Further, understanding the forces that shape these communities requires detailed physical and chemical measurements associated with individual samples to provide information on the selective pressures that might play a role.

**Figure 1:**
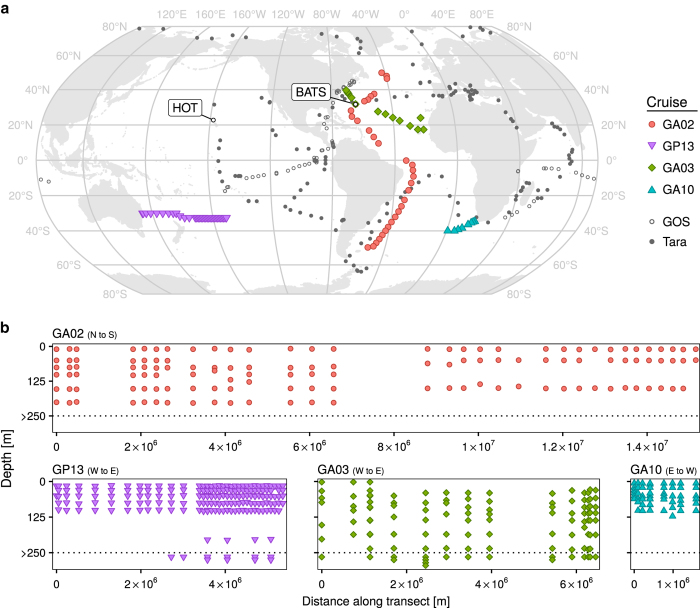
GEOTRACES metagenomic sampling locations. (**a**) Global map indicating the location of each sampling station where metagenomes were collected on the indicated cruise. Sample locations are shown in relation to sites sampled during two other large-scale marine metagenome sampling projects, the GOS and TARA datasets^1–3^ for context. (**b**) Depth distribution of metagenome samples along each of the four GEOTRACES cruises. Transect distances are calculated relative to the first station sampled in the indicated orientation. The depth distribution of samples collected below 250 m are not shown to scale for clarity (ranging from 281–5601 m; see Table 3 (available online only)).

Here we present whole community metagenomic data from 610 samples collected in the Atlantic and Pacific Oceans. These data represent snapshots of microbial communities sampled across space and time, and are associated with physical and chemical measurements which are of value in addressing integrative research questions. The first set of metagenomes, collected under the auspices of the bioGEOTRACES component of the international GEOTRACES program^4^, comprises 480 samples collected in 2010-2011 (Data Citation 1). These samples come from 91 stations visited over four major cruise transects, with 2-10 depths (median 5) sampled at each station (Fig. 1; Table 1). An extensive suite of physical and chemical measurements, comprising over 147 unique data types including salinity, oxygen, temperature, nutrients, and detailed trace metal concentrations^4^, are available for these samples.

**Figure 2:**
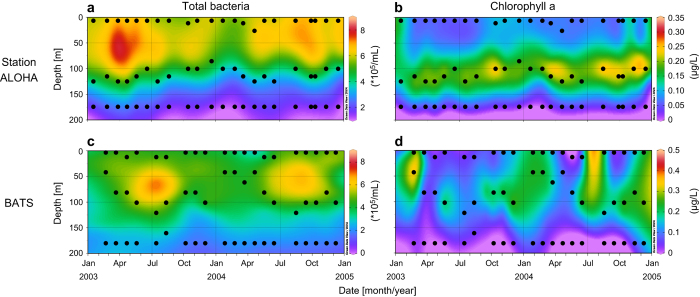
Time-series metagenome sampling. Black dots indicate the time and depth of each sample sequenced from Station ALOHA (Hawai’i Ocean Time-Series; **a**, **b**) and the Bermuda-Atlantic Time Series station (BATS; **c**, **d**). Sampling scheme is depicted in the context of total bacterial counts (**a**, **c**) and chlorophyll abundance (**b**, **d**) data from HOT^22^ and BATS^6^. The middle depth samples were chosen to track the deep chlorophyll maximum.

The second set of metagenomes contains time series data collected at two long-term ocean study sites: Station ALOHA in the North Pacific Subtropical Gyre, sampled as part of the Hawai’i Ocean Time-series (HOT) program^5^, and the Bermuda-Atlantic Time-series Study (BATS) Station^6^ in the Sargasso Sea (Data Citation 2). Water samples were collected every month for two years (2003-2004) at both locations, and we sequenced libraries from 3 depths per month (between 1-180 m), representing surface water, the deep chlorophyll maximum, and the bottom of the euphotic zone (Fig. 2; Table 2). Two additional samples collected from each site in 2009 are also included (Table 3 (available online only)). This temporal sampling scheme provides opportunities to compare and contrast variations within and between these two oligotrophic ocean regimes across seasonal, inter-annual and intra-annual time scales. Station ALOHA, for example, remains stably stratified throughout much of the summer^7^, and is often considered to be a N-limited ecosystem^8,9^; BATS, on the other hand, is subject to deep winter mixing events and is generally considered to be a P-limited system^6^. Both HOT and BATS metagenomes are associated with concurrent measurements of numerous other parameters, including physical characterization (e.g., light, temperature, salinity), nutrient concentrations, biological process rate measurements, and *in situ* cell concentrations^5,6^.

The complete dataset contains over 5 terabases (in 1.67×10^10^ paired-end reads) of raw sequence data (Table 4) (Data Citation 1 and Data Citation 2). In addition to the paired-end reads, we also include a set of assembled contigs from each metagenome library (Data Citation 3 for GEOTRACES and Data Citation 4 for HOT and BATS). As these metagenomes represent the microbial community in whole water samples, sequences from bacteria (39% of reads), archaea (4%), eukaryotes (1%) and viruses (2%) are present in roughly the same proportions observed in other marine datasets^10^. Future improvements in reference databases will likely continue to reduce the number of unidentified reads and refine read recruitment.

We anticipate that these data will be useful for addressing a wide variety of research questions and generating new hypotheses across a broad range of disciplines including, but not limited to, microbial ecology, population genetics, evolution, and oceanography. In particular, the physical, chemical, and biological measurements associated with these samples enable studies of the relationships between microbial community structure, functional potential, biogeochemical cycles, and specific environmental variables.

## Methods

Whole water samples were collected onto 0.2 μm filters and preserved using previously described protocols for qPCR sampling^11–14^. Briefly, water was transferred from the appropriate Niskin bottle into a clean 500 mL amber bottle which had been washed three times with seawater from the same Niskin bottle. Replicate filters were prepared from each water sample by passing 100 mL of seawater through a 25mm diameter, 0.2 μm pore size polycarbonate filter under vacuum (9 in Hg maximum pressure). Filters were then chased with 3 mL of sterile preservation solution (10 mM Tris, pH 8.0; 100 mM EDTA; 0.5 M NaCl) and then immediately transferred to cryovials and stored at -80 °C. All glassware and collection bottles were cleaned in 10% bleach followed by extensive rinsing with 18 mΩ water (Millipore Milli-Q).

Total community DNA was extracted using a phenol/chloroform-based extraction method^15^ that was slightly modified for these samples. Lysing Matrix E beads (MP Biomedicals), 400 ul Phenol:Chloroform:IAA (25:24:1) and 400 ul 2x TENS buffer (100 mM Tris-HCL pH 8.0, 40 mM EDTA, 200 mM NaCl, 2% SDS for 2x buffer) were added to a microcentrifuge tube containing the filter and then vigorously agitated using a beadbeater for 40 seconds. After spinning at 19,000 x*g* for 5 minutes, the aqueous phase was transferred into a Phase Lock Gel tube (5 Prime), mixed with an equal volume of chloroform, and then spun at ~27,000 x *g* for 5 minutes. The supernatant was removed and mixed with an equal volume of AMPure XP beads (Beckman Coulter), and incubated at room temperature for 10 minutes. Beads were washed twice with 75% ethanol, dried, and resuspended in 20 uL ultrapure glass distilled water (Teknova). Total DNA yield was quantified using the PicoGreen assay (ThermoFisher) with yields ranging from ~10-2600 ng total DNA.

Sequencing libraries were prepared and sequenced by the MIT BioMicro Center. Libraries were constructed using the NextEra XT kit (Illumina) on an automated Tecan Freedom EVO robotics platform, starting from 1ng of input DNA. Relevant adapter sequences for downstream quality trimming are 5’-CTGTCTCTTATACACATCTCCGAGCCCACGAGAC-3’ and 5’-CTGTCTCTTATACACATCTGACGCTGCCGACGA-3’. Target library insert length was ~250 nt. The resulting libraries were sequenced using the Illumina NextSeq platform to produce 150+150 nt paired reads. Sixteen metagenomes were multiplexed on each lane, and a median total of ~25 million raw paired-end reads was obtained for each sample (range: ~2.6–323 million, due to variations in library loading).

To characterize the overall taxonomic content of the metagenomes (see Background & Summary), low quality regions of sequencing data and Illumina adapter sequences were first removed using Trimmomatic (V0.36)^16^. The trimmed reads were then assigned taxonomy using Kaiju (V1.5.0)^17^ in MEM mode with the SEG low complexity filter enabled. Kaiju classification employed a database containing the NCBI nr database (consisting of 103 million bacterial, archaeal viral, fungal, and microbial eukaryotic protein sequences; accessed 2017-05-16)^18^, the Moore Foundation Marine Microbial Eukaryote Transcriptome Sequencing Project dataset^19^, and 729 marine single cell genomes^20^. Figures were generated using Ocean Data View (V4.7.10; http://odv.awi.de) and R (V3.3.2; https://www.R-project.org), with the assistance of a number of R-based tools (tidyverse: https://CRAN.R-project.org/package=tidyverse; sp: https://CRAN.R-project.org/package=sp; geosphere: https://CRAN.R-project.org/package=geosphere; patchwork: https://github.com/thomasp85/patchwork; ggworldmap: https://github.com/thackl/ggworldmap).

Metagenome assemblies of each library were generated using metaSPAdes^21^ (v3.9.0 and v3.10.1). Paired-end reads were first quality trimmed with Trimmomatic as above, and then used as input for the metaSPAdes algorithm (with the default --meta settings). Assembled contigs shorter than 200bp were discarded.

### Code availability

No custom code was used to generate or process these data. Software versions and any relevant variables and parameters employed are as follows:

Trimmomatic (V0.36): -phred33 ILLUMINACLIP:NexteraPE-PE.fa:2:30:10

LEADING:3 TRAILING:3 SLIDINGWINDOW:10:20 MINLEN:75

Kaiju (V1.5.0): -a mem -x

SPAdes (V3.9.0): --meta

SPAdes (V3.10.1): --meta

## Data Records

The raw Illumina sequencing reads and sets of assembled contigs for all metagenomes are available from the NCBI Sequence Read Archive (Data Citations 1, 2, 3, 4). Accession numbers, sample date/location, cruise information, and library size for each metagenome can be found in Table 3 (available online only).

## Technical Validation

To confirm the reliability of the automated library preparation steps and ensure that sample cross-contamination was minimized, we randomly included blank buffer samples among our samples and verified that these did not yield successful libraries. Prior to sequencing, the quality of the Illumina libraries was assessed on a Fragment Analyzer (Advanced Analytical) to ensure that the median insert size and overall distribution was in the expected range (peak fragment length ~1200 bp, range 300-3000 bp), with a total yield >1 ng; libraries which did not meet these criteria were reprepared. Whenever possible, libraries with relatively low sequencing coverage (<1×10^7^ paired-end reads) were subjected to additional rounds of sequencing. Sequencing quality (as assessed by per-base average sequence quality scores, quality over the length of the read, kmer overrepresentation, etc) was monitored by the MIT BioMicro Center’s automated sequencing analysis pipelines, and libraries were resequenced if necessary.

## Usage Notes

All metagenomes are associated with standardized GEOTRACES, HOT, and BATS bottle identification numbers to enable cross-referencing with the relevant databases of physical, chemical, and biological measurements (Table 3 (available online only)). GEOTRACES data can be accessed from the British Oceanographic Data Centre (https://www.bodc.ac.uk/geotraces/). HOT data can be accessed from http://hahana.soest.hawaii.edu/hot/hot-dogs/index.html, and BATS data from http://bats.bios.edu/. GEOTRACES data are periodically updated, and users are encouraged to access the most recent data release. Note that each individual metagenome may not have associated measurements for all possible parameters. Access to data from all of these sources is subject to their respective data use policies.

The assembled contigs for each metagenome (Data Citations 3 and Data Citations 4) are deposited in the NCBI Sequence Read Archive as analysis objects with accession numbers as listed in Table 3 (available online only). Assemblies are also available from the iMicrobe database: Geotraces samples at https://www.imicrobe.us/#/projects/277; HOT samples at https://www.imicrobe.us/#/projects/271; and BATS samples at https://www.imicrobe.us/#/projects/276.

Some use cases for the metagenomes could benefit from overlapping the paired-end data to create longer reads for downstream analysis. In our experience, on average 51% of paired reads overlap. We also note that these reads and assembled contigs may contain a small amount of contamination arising from the sampling, library preparation, and/or sequencing steps. While we worked to minimize this as much as possible, note that the data described here have not been pre-screened.

## Additional information

**How to cite this article**: Biller, S. J. *et al*. Marine microbial metagenomes sampled across space and time. *Sci. Data* 5:180176 doi: 10.1038/sdata.2018.176 (2018).

**Publisher’s note**: Springer Nature remains neutral with regard to jurisdictional claims in published maps and institutional affiliations.

## Supplementary Material



## Figures and Tables

**Table 1:** Summary of GEOTRACES cruise metagenome samples.

**Geotraces section ID**	**Location/track**	**Sampling dates**	**# Stations**	**# Depths per station**	**# Samples**	**Available ancillary data fields**	**Ancillary data categories**
GA02	North to South Atlantic	May 2010–March 2011	32	2–6	127	≥39	Physical, Chemical (nutrients, trace metals)
GA03	North Atlantic	October 2010–December 2011	15	6-10	114	≥90	Physical, Chemical (nutrients, trace metals)
GA10	South Atlantic, 40 ºS	October 2010–November 2010	9	6	54	≥85	Physical, Chemical (nutrients, trace metals)
GP13	South Pacific Ocean	May 2011–June 2011	35	2-8	185	≥16	Physical, Chemical (nutrients, trace metals)

**Table 2:** Summary of time series metagenome samples.

**Time series**	**Location**	**Latitude, longitude**	**Cruise numbers**	**Dates sampled**	**# Depths per time point**	**# Samples**	**Available ancillary data fields**	**Ancillary data categories**
BATS	Sargasso Sea	31º 40’ N, 64º 10’ W	173-195	February 2003 - December 2004	3	60	≥27	Physical, Chemical, Biological
HOT	N. Pacific gyre, Station ALOHA	22º 45’ N, 158º W	144-166	January 2003 - December 2004	3	66	≥80	Physical, Chemical, Biological

**Table 3:** Detailed sampling location and accession information for all metagenomic datasets.

**Sample name**	**Cruise series**	**GEOTRACES section**	**Cruise ID**	**Cruise Station**	**Collection Date**	**Depth (m)**	**env_biome**	**env_feature**	**env_material**	**geo_loc_name**	**Latitude and Longitude**	**Bottle ID**	**NCBI BioProject**	**NCBI SRA Study**	**NCBI BioSample**	**NCBI SRA Accession**	**Total read pairs**	**NCBI SRA Accession for assembled metagenome contigs**	**Assembly method and version**
S0001	GEOTRACES	GA02	PE319	10	2010-05-10T12:13:00	10	ocean_biome	ocean	water	Atlantic Ocean	49.7225 N 42.4467 W	631397	PRJNA385854	SRP110813	SAMN07136483	SRR5788236	20058749	SRZ187206	metaSPAdes v3.9.0
S0002	GEOTRACES	GA02	PE319	10	2010-05-10T12:13:00	50	ocean_biome	ocean	water	Atlantic Ocean	49.7225 N 42.4467 W	631391	PRJNA385854	SRP110813	SAMN07136484	SRR5788235	29418997	SRZ187207	metaSPAdes v3.9.0
S0003	GEOTRACES	GA02	PE319	10	2010-05-10T12:13:00	75	ocean_biome	ocean	water	Atlantic Ocean	49.7225 N 42.4467 W	631388	PRJNA385854	SRP110813	SAMN07136485	SRR5788238	28686315	SRZ187208	metaSPAdes v3.9.0
S0004	GEOTRACES	GA02	PE319	10	2010-05-10T12:13:00	101	ocean_biome	ocean	water	Atlantic Ocean	49.7225 N 42.4467 W	631385	PRJNA385854	SRP110813	SAMN07136486	SRR5788237	30938631	SRZ187209	metaSPAdes v3.9.0
S0005	GEOTRACES	GA02	PE319	10	2010-05-10T12:13:00	152	ocean_biome	ocean	water	Atlantic Ocean	49.7225 N 42.4467 W	631382	PRJNA385854	SRP110813	SAMN07136487	SRR5788232	25991174	SRZ187210	metaSPAdes v3.9.0
S0006	GEOTRACES	GA02	PE319	10	2010-05-10T12:13:00	199	ocean_biome	ocean	water	Atlantic Ocean	49.7225 N 42.4467 W	631379	PRJNA385854	SRP110813	SAMN07136488	SRR5788231	21561486	SRZ187211	metaSPAdes v3.9.0
S0008	GEOTRACES	GA02	PE319	12	2010-05-12T20:39:00	10	ocean_biome	ocean	water	Atlantic Ocean	46.312 N 39.658 W	631829	PRJNA385854	SRP110813	SAMN07136489	SRR5788234	25267615	SRZ187212	metaSPAdes v3.9.0
S0009	GEOTRACES	GA02	PE319	12	2010-05-12T20:39:00	49	ocean_biome	ocean	water	Atlantic Ocean	46.312 N 39.658 W	631823	PRJNA385854	SRP110813	SAMN07136490	SRR5788233	26551896	SRZ187213	metaSPAdes v3.9.0
S0010	GEOTRACES	GA02	PE319	12	2010-05-12T20:39:00	73	ocean_biome	ocean	water	Atlantic Ocean	46.312 N 39.658 W	631820	PRJNA385854	SRP110813	SAMN07136491	SRR5788230	25920948	SRZ187214	metaSPAdes v3.9.0
S0011	GEOTRACES	GA02	PE319	12	2010-05-12T20:39:00	100	ocean_biome	ocean	water	Atlantic Ocean	46.312 N 39.658 W	631817	PRJNA385854	SRP110813	SAMN07136492	SRR5788229	33506457	SRZ187215	metaSPAdes v3.9.0
S0012	GEOTRACES	GA02	PE319	12	2010-05-12T20:39:00	153	ocean_biome	ocean	water	Atlantic Ocean	46.312 N 39.658 W	631814	PRJNA385854	SRP110813	SAMN07136493	SRR5788436	25967480	SRZ187216	metaSPAdes v3.9.0
S0013	GEOTRACES	GA02	PE319	12	2010-05-12T20:39:00	198	ocean_biome	ocean	water	Atlantic Ocean	46.312 N 39.658 W	631811	PRJNA385854	SRP110813	SAMN07136494	SRR5788435	28657562	SRZ187217	metaSPAdes v3.9.0
S0015	GEOTRACES	GA02	PE319	16	2010-05-20T13:30:00	8	ocean_biome	ocean	water	Atlantic Ocean	36.2067 N 53.2681 W	632891	PRJNA385854	SRP110813	SAMN07136495	SRR5788434	24265082	SRZ187218	metaSPAdes v3.9.0
S0016	GEOTRACES	GA02	PE319	16	2010-05-20T13:30:00	50	ocean_biome	ocean	water	Atlantic Ocean	36.2067 N 53.2681 W	632885	PRJNA385854	SRP110813	SAMN07136496	SRR5788433	24656118	SRZ187219	metaSPAdes v3.9.0
S0017	GEOTRACES	GA02	PE319	16	2010-05-20T13:30:00	74	ocean_biome	ocean	water	Atlantic Ocean	36.2067 N 53.2681 W	632882	PRJNA385854	SRP110813	SAMN07136497	SRR5788432	23071938	SRZ187220	metaSPAdes v3.9.0
S0018	GEOTRACES	GA02	PE319	16	2010-05-20T13:30:00	102	ocean_biome	ocean	water	Atlantic Ocean	36.2067 N 53.2681 W	632879	PRJNA385854	SRP110813	SAMN07136498	SRR5788431	21337686	SRZ187221	metaSPAdes v3.9.0
S0019	GEOTRACES	GA02	PE319	16	2010-05-20T13:30:00	151	ocean_biome	ocean	water	Atlantic Ocean	36.2067 N 53.2681 W	632876	PRJNA385854	SRP110813	SAMN07136499	SRR5788430	24669237	SRZ187222	metaSPAdes v3.9.0
S0020	GEOTRACES	GA02	PE319	16	2010-05-20T13:30:00	199	ocean_biome	ocean	water	Atlantic Ocean	36.2067 N 53.2681 W	632873	PRJNA385854	SRP110813	SAMN07136500	SRR5788429	17147643	SRZ187223	metaSPAdes v3.9.0
S0021	GEOTRACES	GA02	PE319	15	2010-05-18T13:22:00	10	ocean_biome	ocean	water	Atlantic Ocean	37.5673 N 50.6855 W	632459	PRJNA385854	SRP110813	SAMN07136501	SRR5788428	23533825	SRZ187224	metaSPAdes v3.9.0
S0022	GEOTRACES	GA02	PE319	15	2010-05-18T13:22:00	52	ocean_biome	ocean	water	Atlantic Ocean	37.5673 N 50.6855 W	632453	PRJNA385854	SRP110813	SAMN07136502	SRR5788427	23735638	SRZ187225	metaSPAdes v3.9.0
S0023	GEOTRACES	GA02	PE319	15	2010-05-18T13:22:00	75	ocean_biome	ocean	water	Atlantic Ocean	37.5673 N 50.6855 W	632450	PRJNA385854	SRP110813	SAMN07136503	SRR5788371	23089410	SRZ187226	metaSPAdes v3.9.0
S0024	GEOTRACES	GA02	PE319	15	2010-05-18T13:22:00	103	ocean_biome	ocean	water	Atlantic Ocean	37.5673 N 50.6855 W	632447	PRJNA385854	SRP110813	SAMN07136504	SRR5788372	24224072	SRZ187227	metaSPAdes v3.9.0
S0025	GEOTRACES	GA02	PE319	15	2010-05-18T13:22:00	151	ocean_biome	ocean	water	Atlantic Ocean	37.5673 N 50.6855 W	632444	PRJNA385854	SRP110813	SAMN07136505	SRR5788369	26113050	SRZ187228	metaSPAdes v3.9.0
S0026	GEOTRACES	GA02	PE319	15	2010-05-18T13:22:00	201	ocean_biome	ocean	water	Atlantic Ocean	37.5673 N 50.6855 W	632441	PRJNA385854	SRP110813	SAMN07136506	SRR5788370	24657052	SRZ187229	metaSPAdes v3.9.0
S0027	GEOTRACES	GA02	PE319	17	2010-05-21T13:30:00	10	ocean_biome	ocean	water	Atlantic Ocean	34.3277 N 55.4316 W	632129	PRJNA385854	SRP110813	SAMN07136507	SRR5788367	50998999	SRZ187230	metaSPAdes v3.9.0
S0028	GEOTRACES	GA02	PE319	17	2010-05-21T13:30:00	50	ocean_biome	ocean	water	Atlantic Ocean	34.3277 N 55.4316 W	632123	PRJNA385854	SRP110813	SAMN07136508	SRR5788368	29124395	SRZ187231	metaSPAdes v3.9.0
S0029	GEOTRACES	GA02	PE319	17	2010-05-21T13:30:00	77	ocean_biome	ocean	water	Atlantic Ocean	34.3277 N 55.4316 W	632120	PRJNA385854	SRP110813	SAMN07136509	SRR5788365	36118801	SRZ187232	metaSPAdes v3.9.0
S0030	GEOTRACES	GA02	PE319	17	2010-05-21T13:30:00	103	ocean_biome	ocean	water	Atlantic Ocean	34.3277 N 55.4316 W	632117	PRJNA385854	SRP110813	SAMN07136510	SRR5788366	27750917	SRZ187233	metaSPAdes v3.9.0
S0031	GEOTRACES	GA02	PE319	17	2010-05-21T13:30:00	152	ocean_biome	ocean	water	Atlantic Ocean	34.3277 N 55.4316 W	632114	PRJNA385854	SRP110813	SAMN07136511	SRR5788373	31928726	SRZ187234	metaSPAdes v3.9.0
S0032	GEOTRACES	GA02	PE319	17	2010-05-21T13:30:00	199	ocean_biome	ocean	water	Atlantic Ocean	34.3277 N 55.4316 W	632111	PRJNA385854	SRP110813	SAMN07136512	SRR5788374	30029518	SRZ187235	metaSPAdes v3.9.0
S0033	GEOTRACES	GA02	PE321	33	2010-06-26T12:25:00	9	ocean_biome	ocean	water	Atlantic Ocean	13.1623 N 53.4215 W	636587	PRJNA385854	SRP110813	SAMN07136513	SRR5788197	23050701	SRZ187236	metaSPAdes v3.9.0
S0034	GEOTRACES	GA02	PE321	33	2010-06-26T12:25:00	75	ocean_biome	ocean	water	Atlantic Ocean	13.1623 N 53.4215 W	636578	PRJNA385854	SRP110813	SAMN07136514	SRR5788196	26335287	SRZ187237	metaSPAdes v3.9.0
S0035	GEOTRACES	GA02	PE321	33	2010-06-26T12:25:00	99	ocean_biome	ocean	water	Atlantic Ocean	13.1623 N 53.4215 W	636575	PRJNA385854	SRP110813	SAMN07136515	SRR5788199	27447364	SRZ187238	metaSPAdes v3.9.0
S0036	GEOTRACES	GA02	PE321	33	2010-06-26T12:25:00	150	ocean_biome	ocean	water	Atlantic Ocean	13.1623 N 53.4215 W	636572	PRJNA385854	SRP110813	SAMN07136516	SRR5788198	26750868	SRZ187239	metaSPAdes v3.9.0
S0037	GEOTRACES	GA02	PE321	33	2010-06-26T12:25:00	200	ocean_biome	ocean	water	Atlantic Ocean	13.1623 N 53.4215 W	636569	PRJNA385854	SRP110813	SAMN07136517	SRR5788193	33684808	SRZ187240	metaSPAdes v3.9.0
S0039	GEOTRACES	GA02	PE321	35	2010-06-28T14:16:00	9	ocean_biome	ocean	water	Atlantic Ocean	9.5462 N 50.4686 W	634616	PRJNA385854	SRP110813	SAMN07136518	SRR5788192	26955573	SRZ187241	metaSPAdes v3.9.0
S0040	GEOTRACES	GA02	PE321	35	2010-06-28T14:16:00	72	ocean_biome	ocean	water	Atlantic Ocean	9.5462 N 50.4686 W	634607	PRJNA385854	SRP110813	SAMN07136519	SRR5788195	26308765	SRZ187242	metaSPAdes v3.9.0
S0041	GEOTRACES	GA02	PE321	35	2010-06-28T14:16:00	100	ocean_biome	ocean	water	Atlantic Ocean	9.5462 N 50.4686 W	634604	PRJNA385854	SRP110813	SAMN07136520	SRR5788194	29117451	SRZ187243	metaSPAdes v3.9.0
S0042	GEOTRACES	GA02	PE321	35	2010-06-28T14:16:00	151	ocean_biome	ocean	water	Atlantic Ocean	9.5462 N 50.4686 W	634601	PRJNA385854	SRP110813	SAMN07136521	SRR5788201	20061969	SRZ187244	metaSPAdes v3.9.0
S0043	GEOTRACES	GA02	PE321	35	2010-06-28T14:16:00	201	ocean_biome	ocean	water	Atlantic Ocean	9.5462 N 50.4686 W	634598	PRJNA385854	SRP110813	SAMN07136522	SRR5788200	29398077	SRZ187245	metaSPAdes v3.9.0
S0044	GEOTRACES	GA02	PE321	31	2010-06-24T15:06:00	8	ocean_biome	ocean	water	Atlantic Ocean	16.8312 N 56.2685 W	636299	PRJNA385854	SRP110813	SAMN07136523	SRR5788144	21922297	SRZ187246	metaSPAdes v3.9.0
S0045	GEOTRACES	GA02	PE321	31	2010-06-24T15:06:00	76	ocean_biome	ocean	water	Atlantic Ocean	16.8312 N 56.2685 W	636290	PRJNA385854	SRP110813	SAMN07136524	SRR5788145	27685906	SRZ187247	metaSPAdes v3.9.0
S0046	GEOTRACES	GA02	PE321	31	2010-06-24T15:06:00	101	ocean_biome	ocean	water	Atlantic Ocean	16.8312 N 56.2685 W	636287	PRJNA385854	SRP110813	SAMN07136525	SRR5788146	27382212	SRZ187248	metaSPAdes v3.9.0
S0047	GEOTRACES	GA02	PE321	31	2010-06-24T15:06:00	150	ocean_biome	ocean	water	Atlantic Ocean	16.8312 N 56.2685 W	636284	PRJNA385854	SRP110813	SAMN07136526	SRR5788147	35963444	SRZ187249	metaSPAdes v3.9.0
S0048	GEOTRACES	GA02	PE321	31	2010-06-24T15:06:00	201	ocean_biome	ocean	water	Atlantic Ocean	16.8312 N 56.2685 W	636281	PRJNA385854	SRP110813	SAMN07136527	SRR5788140	24818828	SRZ187250	metaSPAdes v3.9.0
S0049	GEOTRACES	GA03	KN204	8	2011-11-16T13:59:00	31.3	ocean_biome	ocean	water	Atlantic Ocean	35.41617 N 66.54077 W	844604	PRJNA385854	SRP110813	SAMN07136528	SRR5788141	41868308	SRZ187251	metaSPAdes v3.9.0
S0050	GEOTRACES	GA03	KN204	8	2011-11-16T13:59:00	68	ocean_biome	ocean	water	Atlantic Ocean	35.41617 N 66.54077 W	844607	PRJNA385854	SRP110813	SAMN07136529	SRR5788142	18914125	SRZ187252	metaSPAdes v3.9.0
S0051	GEOTRACES	GA03	KN204	8	2011-11-16T13:59:00	111.1	ocean_biome	ocean	water	Atlantic Ocean	35.41617 N 66.54077 W	844610	PRJNA385854	SRP110813	SAMN07136530	SRR5788143	28826994	SRZ187253	metaSPAdes v3.9.0
S0052	GEOTRACES	GA03	KN204	8	2011-11-16T13:59:00	135.8	ocean_biome	ocean	water	Atlantic Ocean	35.41617 N 66.54077 W	844613	PRJNA385854	SRP110813	SAMN07136531	SRR5788138	23095796	SRZ187254	metaSPAdes v3.9.0
S0053	GEOTRACES	GA03	KN204	8	2011-11-16T13:59:00	186.3	ocean_biome	ocean	water	Atlantic Ocean	35.41617 N 66.54077 W	844616	PRJNA385854	SRP110813	SAMN07136532	SRR5788139	31811654	SRZ187255	metaSPAdes v3.9.0
S0054	GEOTRACES	GA03	KN204	8	2011-11-16T13:59:00	237.3	ocean_biome	ocean	water	Atlantic Ocean	35.41617 N 66.54077 W	844619	PRJNA385854	SRP110813	SAMN07136533	SRR5788008	28732390	SRZ187256	metaSPAdes v3.9.0
S0055	GEOTRACES	GA03	KN204	8	2011-11-16T13:59:00	291.5	ocean_biome	ocean	water	Atlantic Ocean	35.41617 N 66.54077 W	844622	PRJNA385854	SRP110813	SAMN07136534	SRR5788007	28395368	SRZ187257	metaSPAdes v3.9.0
S0057	GEOTRACES	GA03	KN204	18	2011-12-01T17:25:00	38.9	ocean_biome	ocean	water	Atlantic Ocean	24.1497 N 40.21786 W	846086	PRJNA385854	SRP110813	SAMN07136535	SRR5788006	32527412	SRZ187258	metaSPAdes v3.9.0
S0058	GEOTRACES	GA03	KN204	18	2011-12-01T17:25:00	65.2	ocean_biome	ocean	water	Atlantic Ocean	24.1497 N 40.21786 W	846089	PRJNA385854	SRP110813	SAMN07136536	SRR5788005	21593384	SRZ187259	metaSPAdes v3.9.0
S0059	GEOTRACES	GA03	KN204	18	2011-12-01T17:25:00	75.1	ocean_biome	ocean	water	Atlantic Ocean	24.1497 N 40.21786 W	846092	PRJNA385854	SRP110813	SAMN07136537	SRR5788012	49524737	SRZ187260	metaSPAdes v3.9.0
S0060	GEOTRACES	GA03	KN204	18	2011-12-01T17:25:00	113.9	ocean_biome	ocean	water	Atlantic Ocean	24.1497 N 40.21786 W	846095	PRJNA385854	SRP110813	SAMN07136538	SRR5788011	34544320	SRZ187261	metaSPAdes v3.9.0
S0061	GEOTRACES	GA03	KN204	18	2011-12-01T17:25:00	184	ocean_biome	ocean	water	Atlantic Ocean	24.1497 N 40.21786 W	846101	PRJNA385854	SRP110813	SAMN07136539	SRR5788010	29858813	SRZ187262	metaSPAdes v3.9.0
S0062	GEOTRACES	GA03	KN204	18	2011-12-01T17:25:00	233	ocean_biome	ocean	water	Atlantic Ocean	24.1497 N 40.21786 W	846104	PRJNA385854	SRP110813	SAMN07136540	SRR5788009	31210233	SRZ187263	metaSPAdes v3.9.0
S0063	GEOTRACES	GA03	KN204	18	2011-12-01T17:25:00	283.9	ocean_biome	ocean	water	Atlantic Ocean	24.1497 N 40.21786 W	846107	PRJNA385854	SRP110813	SAMN07136541	SRR5788014	27718979	SRZ187264	metaSPAdes v3.9.0
S0064	GEOTRACES	GA03	KN204	3	2011-11-10T14:23:00	28.4	ocean_biome	ocean	water	Atlantic Ocean	38.65497 N 69.13131 W	845300	PRJNA385854	SRP110813	SAMN07136542	SRR5788013	15194120	SRZ187265	metaSPAdes v3.9.0
S0065	GEOTRACES	GA03	KN204	3	2011-11-10T14:23:00	61.2	ocean_biome	ocean	water	Atlantic Ocean	38.65497 N 69.13131 W	845303	PRJNA385854	SRP110813	SAMN07136543	SRR5788065	17515188	SRZ187266	metaSPAdes v3.9.0
S0066	GEOTRACES	GA03	KN204	3	2011-11-10T14:23:00	86.4	ocean_biome	ocean	water	Atlantic Ocean	38.65497 N 69.13131 W	845306	PRJNA385854	SRP110813	SAMN07136544	SRR5788066	18259926	SRZ187267	metaSPAdes v3.9.0
S0067	GEOTRACES	GA03	KN204	3	2011-11-10T14:23:00	108.8	ocean_biome	ocean	water	Atlantic Ocean	38.65497 N 69.13131 W	845309	PRJNA385854	SRP110813	SAMN07136545	SRR5788063	17719388	SRZ187268	metaSPAdes v3.9.0
S0068	GEOTRACES	GA03	KN204	3	2011-11-10T14:23:00	135.6	ocean_biome	ocean	water	Atlantic Ocean	38.65497 N 69.13131 W	845312	PRJNA385854	SRP110813	SAMN07136546	SRR5788064	32346922	SRZ187269	metaSPAdes v3.9.0
S0069	GEOTRACES	GA03	KN204	3	2011-11-10T14:23:00	186.5	ocean_biome	ocean	water	Atlantic Ocean	38.65497 N 69.13131 W	845315	PRJNA385854	SRP110813	SAMN07136547	SRR5788069	30650936	SRZ187270	metaSPAdes v3.9.0
S0070	GEOTRACES	GA03	KN204	3	2011-11-10T14:23:00	234.7	ocean_biome	ocean	water	Atlantic Ocean	38.65497 N 69.13131 W	845318	PRJNA385854	SRP110813	SAMN07136548	SRR5788070	30836529	SRZ187271	metaSPAdes v3.9.0
S0071	GEOTRACES	GA03	KN204	3	2011-11-10T14:23:00	288.8	ocean_biome	ocean	water	Atlantic Ocean	38.65497 N 69.13131 W	845321	PRJNA385854	SRP110813	SAMN07136549	SRR5788067	36118620	SRZ187272	metaSPAdes v3.9.0
S0072	GEOTRACES	GA03	KN204	20	2011-12-03T23:12:00	39.9	ocean_biome	ocean	water	Atlantic Ocean	22.33305 N 35.86703 W	846236	PRJNA385854	SRP110813	SAMN07136550	SRR5788068	18092283	SRZ187273	metaSPAdes v3.9.0
S0073	GEOTRACES	GA03	KN204	20	2011-12-03T23:12:00	74.3	ocean_biome	ocean	water	Atlantic Ocean	22.33305 N 35.86703 W	846239	PRJNA385854	SRP110813	SAMN07136551	SRR5788061	15618010	SRZ187274	metaSPAdes v3.9.0
S0074	GEOTRACES	GA03	KN204	20	2011-12-03T23:12:00	99.7	ocean_biome	ocean	water	Atlantic Ocean	22.33305 N 35.86703 W	846242	PRJNA385854	SRP110813	SAMN07136552	SRR5788062	29168249	SRZ187275	metaSPAdes v3.9.0
S0075	GEOTRACES	GA03	KN204	20	2011-12-03T23:12:00	134.2	ocean_biome	ocean	water	Atlantic Ocean	22.33305 N 35.86703 W	846245	PRJNA385854	SRP110813	SAMN07136553	SRR5788107	35016855	SRZ187276	metaSPAdes v3.9.0
S0076	GEOTRACES	GA03	KN204	20	2011-12-03T23:12:00	183.8	ocean_biome	ocean	water	Atlantic Ocean	22.33305 N 35.86703 W	846248	PRJNA385854	SRP110813	SAMN07136554	SRR5788204	37258714	SRZ187277	metaSPAdes v3.9.0
S0077	GEOTRACES	GA03	KN204	20	2011-12-03T23:12:00	234.1	ocean_biome	ocean	water	Atlantic Ocean	22.33305 N 35.86703 W	846251	PRJNA385854	SRP110813	SAMN07136555	SRR5788214	35121420	SRZ187278	metaSPAdes v3.9.0
S0078	GEOTRACES	GA03	KN204	20	2011-12-03T23:12:00	300.8	ocean_biome	ocean	water	Atlantic Ocean	22.33305 N 35.86703 W	846254	PRJNA385854	SRP110813	SAMN07136556	SRR5788213	27625031	SRZ187279	metaSPAdes v3.9.0
S0079	GEOTRACES	GA03	KN204	20	2011-12-03T23:12:00	374.3	ocean_biome	ocean	water	Atlantic Ocean	22.33305 N 35.86703 W	846257	PRJNA385854	SRP110813	SAMN07136557	SRR5788036	27720001	SRZ187280	metaSPAdes v3.9.0
S0080	GEOTRACES	GA03	KN204	20	2011-12-03T23:12:00	499.6	ocean_biome	ocean	water	Atlantic Ocean	22.33305 N 35.86703 W	846260	PRJNA385854	SRP110813	SAMN07136558	SRR5788215	30757878	SRZ187281	metaSPAdes v3.9.0
S0081	GEOTRACES	GA03	KN204	20	2011-12-03T23:12:00	1055.2	ocean_biome	ocean	water	Atlantic Ocean	22.33305 N 35.86703 W	846269	PRJNA385854	SRP110813	SAMN07136559	SRR5788228	36015165	SRZ187282	metaSPAdes v3.9.0
S0082	GEOTRACES	GA03	KN204	10	2011-11-19T19:31:00	41.8	ocean_biome	ocean	water	Atlantic Ocean	31.75351 N 64.17499 W	844712	PRJNA385854	SRP110813	SAMN07136560	SRR5788224	33877240	SRZ187283	metaSPAdes v3.9.0
S0083	GEOTRACES	GA03	KN204	10	2011-11-19T19:31:00	75.7	ocean_biome	ocean	water	Atlantic Ocean	31.75351 N 64.17499 W	844715	PRJNA385854	SRP110813	SAMN07136561	SRR5788313	33475023	SRZ187284	metaSPAdes v3.9.0
S0084	GEOTRACES	GA03	KN204	10	2011-11-19T19:31:00	89.1	ocean_biome	ocean	water	Atlantic Ocean	31.75351 N 64.17499 W	844718	PRJNA385854	SRP110813	SAMN07136562	SRR5788312	31453590	SRZ187285	metaSPAdes v3.9.0
S0085	GEOTRACES	GA03	KN204	10	2011-11-19T19:31:00	109.7	ocean_biome	ocean	water	Atlantic Ocean	31.75351 N 64.17499 W	844721	PRJNA385854	SRP110813	SAMN07136563	SRR5787993	28189267	SRZ187286	metaSPAdes v3.9.0
S0086	GEOTRACES	GA03	KN204	10	2011-11-19T19:31:00	182.4	ocean_biome	ocean	water	Atlantic Ocean	31.75351 N 64.17499 W	844724	PRJNA385854	SRP110813	SAMN07136564	SRR5787994	26364899	SRZ187287	metaSPAdes v3.9.0
S0087	GEOTRACES	GA03	KN204	10	2011-11-19T19:31:00	233.3	ocean_biome	ocean	water	Atlantic Ocean	31.75351 N 64.17499 W	844727	PRJNA385854	SRP110813	SAMN07136565	SRR5788249	25994646	SRZ187288	metaSPAdes v3.9.0
S0088	GEOTRACES	GA03	KN204	10	2011-11-19T19:31:00	281.4	ocean_biome	ocean	water	Atlantic Ocean	31.75351 N 64.17499 W	844730	PRJNA385854	SRP110813	SAMN07136566	SRR5788250	35932845	SRZ187289	metaSPAdes v3.9.0
S0089	GEOTRACES	GA03	KN204	10	2011-11-19T19:31:00	414.2	ocean_biome	ocean	water	Atlantic Ocean	31.75351 N 64.17499 W	844733	PRJNA385854	SRP110813	SAMN07136567	SRR5788251	22021412	SRZ187290	metaSPAdes v3.9.0
S0090	GEOTRACES	GA03	KN204	16	2011-11-30T03:40:00	39.7	ocean_biome	ocean	water	Atlantic Ocean	26.13688 N 44.82622 W	845942	PRJNA385854	SRP110813	SAMN07136568	SRR5788252	31728111	SRZ187291	metaSPAdes v3.9.0
S0091	GEOTRACES	GA03	KN204	16	2011-11-30T03:40:00	60.7	ocean_biome	ocean	water	Atlantic Ocean	26.13688 N 44.82622 W	845945	PRJNA385854	SRP110813	SAMN07136569	SRR5788253	23180825	SRZ187292	metaSPAdes v3.9.0
S0092	GEOTRACES	GA03	KN204	16	2011-11-30T03:40:00	89.9	ocean_biome	ocean	water	Atlantic Ocean	26.13688 N 44.82622 W	845948	PRJNA385854	SRP110813	SAMN07136570	SRR5788254	30362911	SRZ187293	metaSPAdes v3.9.0
S0093	GEOTRACES	GA03	KN204	16	2011-11-30T03:40:00	110.7	ocean_biome	ocean	water	Atlantic Ocean	26.13688 N 44.82622 W	845951	PRJNA385854	SRP110813	SAMN07136571	SRR5788025	33454639	SRZ187294	metaSPAdes v3.9.0
S0094	GEOTRACES	GA03	KN204	16	2011-11-30T03:40:00	135.6	ocean_biome	ocean	water	Atlantic Ocean	26.13688 N 44.82622 W	845954	PRJNA385854	SRP110813	SAMN07136572	SRR5788225	32430476	SRZ187295	metaSPAdes v3.9.0
S0095	GEOTRACES	GA03	KN204	16	2011-11-30T03:40:00	185.7	ocean_biome	ocean	water	Atlantic Ocean	26.13688 N 44.82622 W	845957	PRJNA385854	SRP110813	SAMN07136573	SRR5788414	49713939	SRZ187296	metaSPAdes v3.9.0
S0096	GEOTRACES	GA03	KN204	16	2011-11-30T03:40:00	234.8	ocean_biome	ocean	water	Atlantic Ocean	26.13688 N 44.82622 W	845960	PRJNA385854	SRP110813	SAMN07136574	SRR5788413	38852478	SRZ187297	metaSPAdes v3.9.0
S0097	GEOTRACES	GA03	KN204	16	2011-11-30T03:40:00	284.8	ocean_biome	ocean	water	Atlantic Ocean	26.13688 N 44.82622 W	845963	PRJNA385854	SRP110813	SAMN07136575	SRR5788412	49405382	SRZ187298	metaSPAdes v3.9.0
S0098	GEOTRACES	GA03	KN204	24	2011-12-10T08:27:00	48.1	ocean_biome	ocean	water	Atlantic Ocean	17.39998 N 24.50003 W	846713	PRJNA385854	SRP110813	SAMN07136576	SRR5788411	29057352	SRZ187299	metaSPAdes v3.9.0
S0099	GEOTRACES	GA03	KN204	24	2011-12-10T08:27:00	71.6	ocean_biome	ocean	water	Atlantic Ocean	17.39998 N 24.50003 W	846716	PRJNA385854	SRP110813	SAMN07136577	SRR5788410	30861159	SRZ187300	metaSPAdes v3.9.0
S0100	GEOTRACES	GA03	KN204	24	2011-12-10T08:27:00	89.3	ocean_biome	ocean	water	Atlantic Ocean	17.39998 N 24.50003 W	846719	PRJNA385854	SRP110813	SAMN07136578	SRR5788409	26991060	SRZ187301	metaSPAdes v3.9.0
S0101	GEOTRACES	GA03	KN204	24	2011-12-10T08:27:00	135	ocean_biome	ocean	water	Atlantic Ocean	17.39998 N 24.50003 W	846722	PRJNA385854	SRP110813	SAMN07136579	SRR5788408	31666043	SRZ187302	metaSPAdes v3.9.0
S0102	GEOTRACES	GA03	KN204	24	2011-12-10T08:27:00	183.7	ocean_biome	ocean	water	Atlantic Ocean	17.39998 N 24.50003 W	846725	PRJNA385854	SRP110813	SAMN07136580	SRR5788407	20144298	SRZ187303	metaSPAdes v3.9.0
S0103	GEOTRACES	GA03	KN204	24	2011-12-10T08:27:00	234.5	ocean_biome	ocean	water	Atlantic Ocean	17.39998 N 24.50003 W	846728	PRJNA385854	SRP110813	SAMN07136581	SRR5788426	26744312	SRZ187304	metaSPAdes v3.9.0
S0104	GEOTRACES	GA03	KN204	24	2011-12-10T08:27:00	285.9	ocean_biome	ocean	water	Atlantic Ocean	17.39998 N 24.50003 W	846731	PRJNA385854	SRP110813	SAMN07136582	SRR5788425	29482548	SRZ187305	metaSPAdes v3.9.0
S0105	GEOTRACES	GA03	KN204	14	2011-11-26T15:01:00	40.1	ocean_biome	ocean	water	Atlantic Ocean	27.58305 N 49.63297 W	845645	PRJNA385854	SRP110813	SAMN07136583	SRR5788087	30438461	SRZ187306	metaSPAdes v3.9.0
S0106	GEOTRACES	GA03	KN204	14	2011-11-26T15:01:00	69.4	ocean_biome	ocean	water	Atlantic Ocean	27.58305 N 49.63297 W	845648	PRJNA385854	SRP110813	SAMN07136584	SRR5788088	27613431	SRZ187307	metaSPAdes v3.9.0
S0107	GEOTRACES	GA03	KN204	14	2011-11-26T15:01:00	99	ocean_biome	ocean	water	Atlantic Ocean	27.58305 N 49.63297 W	845651	PRJNA385854	SRP110813	SAMN07136585	SRR5788085	23444487	SRZ187308	metaSPAdes v3.9.0
S0108	GEOTRACES	GA03	KN204	14	2011-11-26T15:01:00	113.8	ocean_biome	ocean	water	Atlantic Ocean	27.58305 N 49.63297 W	845654	PRJNA385854	SRP110813	SAMN07136586	SRR5788086	20474076	SRZ187309	metaSPAdes v3.9.0
S0109	GEOTRACES	GA03	KN204	14	2011-11-26T15:01:00	133.7	ocean_biome	ocean	water	Atlantic Ocean	27.58305 N 49.63297 W	845657	PRJNA385854	SRP110813	SAMN07136587	SRR5788083	20616566	SRZ187310	metaSPAdes v3.9.0
S0110	GEOTRACES	GA03	KN204	14	2011-11-26T15:01:00	183.8	ocean_biome	ocean	water	Atlantic Ocean	27.58305 N 49.63297 W	845660	PRJNA385854	SRP110813	SAMN07136588	SRR5788084	25598730	SRZ187311	metaSPAdes v3.9.0
S0111	GEOTRACES	GA03	KN204	14	2011-11-26T15:01:00	233.6	ocean_biome	ocean	water	Atlantic Ocean	27.58305 N 49.63297 W	845663	PRJNA385854	SRP110813	SAMN07136589	SRR5788081	26758042	SRZ187312	metaSPAdes v3.9.0
S0112	GEOTRACES	GA03	KN204	14	2011-11-26T15:01:00	285	ocean_biome	ocean	water	Atlantic Ocean	27.58305 N 49.63297 W	845666	PRJNA385854	SRP110813	SAMN07136590	SRR5788082	29851182	SRZ187313	metaSPAdes v3.9.0
S0114	GEOTRACES	GA03	KN204	1	2011-11-08T10:56:00	30.2	ocean_biome	ocean	water	Atlantic Ocean	39.69225 N 69.86286 W	845123	PRJNA385854	SRP110813	SAMN07136591	SRR5788089	28961579	SRZ187314	metaSPAdes v3.9.0
S0115	GEOTRACES	GA03	KN204	1	2011-11-08T10:56:00	57.8	ocean_biome	ocean	water	Atlantic Ocean	39.69225 N 69.86286 W	845126	PRJNA385854	SRP110813	SAMN07136592	SRR5788090	27615447	SRZ187315	metaSPAdes v3.9.0
S0116	GEOTRACES	GA03	KN204	1	2011-11-08T10:56:00	89.2	ocean_biome	ocean	water	Atlantic Ocean	39.69225 N 69.86286 W	845834	PRJNA385854	SRP110813	SAMN07136593	SRR5788221	15631705	SRZ187316	metaSPAdes v3.9.0
S0117	GEOTRACES	GA03	KN204	1	2011-11-08T10:56:00	109.9	ocean_biome	ocean	water	Atlantic Ocean	39.69225 N 69.86286 W	845129	PRJNA385854	SRP110813	SAMN07136594	SRR5788220	49471178	SRZ187317	metaSPAdes v3.9.0
S0118	GEOTRACES	GA03	KN204	1	2011-11-08T10:56:00	135.4	ocean_biome	ocean	water	Atlantic Ocean	39.69225 N 69.86286 W	845132	PRJNA385854	SRP110813	SAMN07136595	SRR5788223	26010726	SRZ187318	metaSPAdes v3.9.0
S0119	GEOTRACES	GA03	KN204	1	2011-11-08T10:56:00	185.8	ocean_biome	ocean	water	Atlantic Ocean	39.69225 N 69.86286 W	845135	PRJNA385854	SRP110813	SAMN07136596	SRR5788222	23609025	SRZ187319	metaSPAdes v3.9.0
S0120	GEOTRACES	GA03	KN204	1	2011-11-08T10:56:00	285.9	ocean_biome	ocean	water	Atlantic Ocean	39.69225 N 69.86286 W	845141	PRJNA385854	SRP110813	SAMN07136597	SRR5788217	35587520	SRZ187320	metaSPAdes v3.9.0
S0122	GEOTRACES	GA03	KN204	4	2011-11-12T14:57:00	39.4	ocean_biome	ocean	water	Atlantic Ocean	38.32433 N 68.87553 W	844343	PRJNA385854	SRP110813	SAMN07136598	SRR5788216	35734267	SRZ187321	metaSPAdes v3.9.0
S0123	GEOTRACES	GA03	KN204	4	2011-11-12T14:57:00	65.6	ocean_biome	ocean	water	Atlantic Ocean	38.32433 N 68.87553 W	844346	PRJNA385854	SRP110813	SAMN07136599	SRR5788219	25283409	SRZ187322	metaSPAdes v3.9.0
S0124	GEOTRACES	GA03	KN204	4	2011-11-12T14:57:00	90.8	ocean_biome	ocean	water	Atlantic Ocean	38.32433 N 68.87553 W	844349	PRJNA385854	SRP110813	SAMN07136600	SRR5788218	22509006	SRZ187323	metaSPAdes v3.9.0
S0125	GEOTRACES	GA03	KN204	4	2011-11-12T14:57:00	111.1	ocean_biome	ocean	water	Atlantic Ocean	38.32433 N 68.87553 W	844352	PRJNA385854	SRP110813	SAMN07136601	SRR5788227	20589622	SRZ187324	metaSPAdes v3.9.0
S0126	GEOTRACES	GA03	KN204	4	2011-11-12T14:57:00	135.2	ocean_biome	ocean	water	Atlantic Ocean	38.32433 N 68.87553 W	844355	PRJNA385854	SRP110813	SAMN07136602	SRR5788226	18701879	SRZ187325	metaSPAdes v3.9.0
S0127	GEOTRACES	GA03	KN204	4	2011-11-12T14:57:00	184.1	ocean_biome	ocean	water	Atlantic Ocean	38.32433 N 68.87553 W	844358	PRJNA385854	SRP110813	SAMN07136603	SRR5788320	18140701	SRZ187326	metaSPAdes v3.9.0
S0128	GEOTRACES	GA03	KN204	4	2011-11-12T14:57:00	236.7	ocean_biome	ocean	water	Atlantic Ocean	38.32433 N 68.87553 W	844361	PRJNA385854	SRP110813	SAMN07136604	SRR5788321	16948875	SRZ187327	metaSPAdes v3.9.0
S0129	GEOTRACES	GA03	KN204	4	2011-11-12T14:57:00	285.3	ocean_biome	ocean	water	Atlantic Ocean	38.32433 N 68.87553 W	844364	PRJNA385854	SRP110813	SAMN07136605	SRR5788322	19695567	SRZ187328	metaSPAdes v3.9.0
S0130	GEOTRACES	GA03	KN204	6	2011-11-15T06:20:00	39.6	ocean_biome	ocean	water	Atlantic Ocean	37.60731 N 68.3895 W	844529	PRJNA385854	SRP110813	SAMN07136606	SRR5788323	22871300	SRZ187329	metaSPAdes v3.9.0
S0131	GEOTRACES	GA03	KN204	6	2011-11-15T06:20:00	64.6	ocean_biome	ocean	water	Atlantic Ocean	37.60731 N 68.3895 W	844532	PRJNA385854	SRP110813	SAMN07136607	SRR5788316	20644218	SRZ187330	metaSPAdes v3.9.0
S0132	GEOTRACES	GA03	KN204	6	2011-11-15T06:20:00	89.6	ocean_biome	ocean	water	Atlantic Ocean	37.60731 N 68.3895 W	844535	PRJNA385854	SRP110813	SAMN07136608	SRR5788317	22698692	SRZ187331	metaSPAdes v3.9.0
S0133	GEOTRACES	GA03	KN204	6	2011-11-15T06:20:00	111.1	ocean_biome	ocean	water	Atlantic Ocean	37.60731 N 68.3895 W	844538	PRJNA385854	SRP110813	SAMN07136609	SRR5788318	22766638	SRZ187332	metaSPAdes v3.9.0
S0134	GEOTRACES	GA03	KN204	6	2011-11-15T06:20:00	139.6	ocean_biome	ocean	water	Atlantic Ocean	37.60731 N 68.3895 W	844541	PRJNA385854	SRP110813	SAMN07136610	SRR5788319	18948044	SRZ187333	metaSPAdes v3.9.0
S0135	GEOTRACES	GA03	KN204	6	2011-11-15T06:20:00	160	ocean_biome	ocean	water	Atlantic Ocean	37.60731 N 68.3895 W	844544	PRJNA385854	SRP110813	SAMN07136611	SRR5788314	19618651	SRZ187334	metaSPAdes v3.9.0
S0136	GEOTRACES	GA03	KN204	6	2011-11-15T06:20:00	232.9	ocean_biome	ocean	water	Atlantic Ocean	37.60731 N 68.3895 W	844547	PRJNA385854	SRP110813	SAMN07136612	SRR5788315	23279778	SRZ187335	metaSPAdes v3.9.0
S0137	GEOTRACES	GA03	KN204	6	2011-11-15T06:20:00	284.7	ocean_biome	ocean	water	Atlantic Ocean	37.60731 N 68.3895 W	844550	PRJNA385854	SRP110813	SAMN07136613	SRR5788452	25544472	SRZ187336	metaSPAdes v3.9.0
S0138	GEOTRACES	GA03	KN204	22	2011-12-07T11:25:00	50	ocean_biome	ocean	water	Atlantic Ocean	19.43336 N 29.38323 W	846488	PRJNA385854	SRP110813	SAMN07136614	SRR5788451	26189052	SRZ187337	metaSPAdes v3.9.0
S0139	GEOTRACES	GA03	KN204	22	2011-12-07T11:25:00	74.5	ocean_biome	ocean	water	Atlantic Ocean	19.43336 N 29.38323 W	846491	PRJNA385854	SRP110813	SAMN07136615	SRR5788450	25116828	SRZ187338	metaSPAdes v3.9.0
S0140	GEOTRACES	GA03	KN204	22	2011-12-07T11:25:00	84	ocean_biome	ocean	water	Atlantic Ocean	19.43336 N 29.38323 W	846494	PRJNA385854	SRP110813	SAMN07136616	SRR5788449	23605125	SRZ187339	metaSPAdes v3.9.0
S0141	GEOTRACES	GA03	KN204	22	2011-12-07T11:25:00	123.8	ocean_biome	ocean	water	Atlantic Ocean	19.43336 N 29.38323 W	846497	PRJNA385854	SRP110813	SAMN07136617	SRR5788456	17687887	SRZ187340	metaSPAdes v3.9.0
S0142	GEOTRACES	GA03	KN204	22	2011-12-07T11:25:00	183.9	ocean_biome	ocean	water	Atlantic Ocean	19.43336 N 29.38323 W	846500	PRJNA385854	SRP110813	SAMN07136618	SRR5788455	27277045	SRZ187341	metaSPAdes v3.9.0
S0143	GEOTRACES	GA03	KN204	22	2011-12-07T11:25:00	233.1	ocean_biome	ocean	water	Atlantic Ocean	19.43336 N 29.38323 W	846503	PRJNA385854	SRP110813	SAMN07136619	SRR5788454	25965736	SRZ187342	metaSPAdes v3.9.0
S0144	GEOTRACES	GA03	KN204	22	2011-12-07T11:25:00	283.3	ocean_biome	ocean	water	Atlantic Ocean	19.43336 N 29.38323 W	846506	PRJNA385854	SRP110813	SAMN07136620	SRR5788453	29159150	SRZ187343	metaSPAdes v3.9.0
S0145	GEOTRACES	GA03	KN204	22	2011-12-07T11:25:00	389.4	ocean_biome	ocean	water	Atlantic Ocean	19.43336 N 29.38323 W	846509	PRJNA385854	SRP110813	SAMN07136621	SRR5788448	19481861	SRZ187344	metaSPAdes v3.9.0
S0147	GEOTRACES	GA02	PE321	25	2010-06-17T19:56:00	11	ocean_biome	ocean	water	Atlantic Ocean	24.7143 N 67.0726 W	633245	PRJNA385854	SRP110813	SAMN07136622	SRR5788447	26848483	SRZ187345	metaSPAdes v3.9.0
S0148	GEOTRACES	GA02	PE321	25	2010-06-17T19:56:00	77	ocean_biome	ocean	water	Atlantic Ocean	24.7143 N 67.0726 W	633236	PRJNA385854	SRP110813	SAMN07136623	SRR5788112	31001923	SRZ187346	metaSPAdes v3.9.0
S0149	GEOTRACES	GA02	PE321	25	2010-06-17T19:56:00	119	ocean_biome	ocean	water	Atlantic Ocean	24.7143 N 67.0726 W	633233	PRJNA385854	SRP110813	SAMN07136624	SRR5788113	22977267	SRZ187347	metaSPAdes v3.9.0
S0150	GEOTRACES	GA02	PE321	25	2010-06-17T19:56:00	152	ocean_biome	ocean	water	Atlantic Ocean	24.7143 N 67.0726 W	633230	PRJNA385854	SRP110813	SAMN07136625	SRR5788110	34290195	SRZ187348	metaSPAdes v3.9.0
S0151	GEOTRACES	GA02	PE321	25	2010-06-17T19:56:00	202	ocean_biome	ocean	water	Atlantic Ocean	24.7143 N 67.0726 W	633227	PRJNA385854	SRP110813	SAMN07136626	SRR5788111	27910895	SRZ187349	metaSPAdes v3.9.0
S0152	GEOTRACES	GA02	PE321	27	2010-06-19T21:44:00	12	ocean_biome	ocean	water	Atlantic Ocean	22.3411 N 63.5833 W	633533	PRJNA385854	SRP110813	SAMN07136627	SRR5788116	26336708	SRZ187350	metaSPAdes v3.10.1
S0153	GEOTRACES	GA02	PE321	27	2010-06-19T21:44:00	75	ocean_biome	ocean	water	Atlantic Ocean	22.3411 N 63.5833 W	633524	PRJNA385854	SRP110813	SAMN07136628	SRR5788117	24569068	SRZ187351	metaSPAdes v3.9.0
S0154	GEOTRACES	GA02	PE321	27	2010-06-19T21:44:00	100	ocean_biome	ocean	water	Atlantic Ocean	22.3411 N 63.5833 W	633521	PRJNA385854	SRP110813	SAMN07136629	SRR5788114	38849924	SRZ187352	metaSPAdes v3.9.0
S0155	GEOTRACES	GA02	PE321	27	2010-06-19T21:44:00	127	ocean_biome	ocean	water	Atlantic Ocean	22.3411 N 63.5833 W	633518	PRJNA385854	SRP110813	SAMN07136630	SRR5788115	36820676	SRZ187353	metaSPAdes v3.9.0
S0156	GEOTRACES	GA02	PE321	27	2010-06-19T21:44:00	200	ocean_biome	ocean	water	Atlantic Ocean	22.3411 N 63.5833 W	633515	PRJNA385854	SRP110813	SAMN07136631	SRR5788108	36687766	SRZ187354	metaSPAdes v3.9.0
S0157	GEOTRACES	GA02	PE319	11	2010-05-11T13:26:00	9	ocean_biome	ocean	water	Atlantic Ocean	47.8007 N 39.399 W	631541	PRJNA385854	SRP110813	SAMN07136632	SRR5788109	31062384	SRZ187355	metaSPAdes v3.9.0
S0158	GEOTRACES	GA02	PE319	11	2010-05-11T13:26:00	50	ocean_biome	ocean	water	Atlantic Ocean	47.8007 N 39.399 W	631535	PRJNA385854	SRP110813	SAMN07136633	SRR5788284	17736592	SRZ187356	metaSPAdes v3.9.0
S0159	GEOTRACES	GA02	PE319	11	2010-05-11T13:26:00	75	ocean_biome	ocean	water	Atlantic Ocean	47.8007 N 39.399 W	631532	PRJNA385854	SRP110813	SAMN07136634	SRR5788283	142905804	SRZ187357	metaSPAdes v3.9.0
S0160	GEOTRACES	GA02	PE319	11	2010-05-11T13:26:00	101	ocean_biome	ocean	water	Atlantic Ocean	47.8007 N 39.399 W	631529	PRJNA385854	SRP110813	SAMN07136635	SRR5788286	39217331	SRZ187358	metaSPAdes v3.9.0
S0161	GEOTRACES	GA02	PE319	11	2010-05-11T13:26:00	152	ocean_biome	ocean	water	Atlantic Ocean	47.8007 N 39.399 W	631526	PRJNA385854	SRP110813	SAMN07136636	SRR5788285	20179411	SRZ187359	metaSPAdes v3.9.0
S0162	GEOTRACES	GA02	PE319	11	2010-05-11T13:26:00	201	ocean_biome	ocean	water	Atlantic Ocean	47.8007 N 39.399 W	631523	PRJNA385854	SRP110813	SAMN07136637	SRR5788288	37928461	SRZ187360	metaSPAdes v3.9.0
S0163	GEOTRACES	GA02	PE321	21	2010-06-13T00:53:00	10	ocean_biome	ocean	water	Atlantic Ocean	31.6667 N 64.167 W	633860	PRJNA385854	SRP110813	SAMN07136638	SRR5788287	38006969	SRZ187361	metaSPAdes v3.9.0
S0164	GEOTRACES	GA02	PE321	21	2010-06-13T00:53:00	75	ocean_biome	ocean	water	Atlantic Ocean	31.6667 N 64.167 W	633851	PRJNA385854	SRP110813	SAMN07136639	SRR5788290	32677279	SRZ187362	metaSPAdes v3.9.0
S0165	GEOTRACES	GA02	PE321	21	2010-06-13T00:53:00	100	ocean_biome	ocean	water	Atlantic Ocean	31.6667 N 64.167 W	633848	PRJNA385854	SRP110813	SAMN07136640	SRR5788289	31425941	SRZ187363	metaSPAdes v3.9.0
S0166	GEOTRACES	GA02	PE321	21	2010-06-13T00:53:00	150	ocean_biome	ocean	water	Atlantic Ocean	31.6667 N 64.167 W	633845	PRJNA385854	SRP110813	SAMN07136641	SRR5788282	24904082	SRZ187364	metaSPAdes v3.9.0
S0167	GEOTRACES	GA02	PE321	21	2010-06-13T00:53:00	202	ocean_biome	ocean	water	Atlantic Ocean	31.6667 N 64.167 W	633842	PRJNA385854	SRP110813	SAMN07136642	SRR5788281	18740939	SRZ187365	metaSPAdes v3.9.0
S0168	GEOTRACES	GA02	PE321	23	2010-06-16T00:22:00	8	ocean_biome	ocean	water	Atlantic Ocean	28.0905 N 67.5015 W	634370	PRJNA385854	SRP110813	SAMN07136643	SRR5788375	37873615	SRZ187366	metaSPAdes v3.9.0
S0169	GEOTRACES	GA02	PE321	23	2010-06-16T00:22:00	76	ocean_biome	ocean	water	Atlantic Ocean	28.0905 N 67.5015 W	634361	PRJNA385854	SRP110813	SAMN07136644	SRR5788376	19720275	SRZ187367	metaSPAdes v3.9.0
S0170	GEOTRACES	GA02	PE321	23	2010-06-16T00:22:00	87	ocean_biome	ocean	water	Atlantic Ocean	28.0905 N 67.5015 W	634358	PRJNA385854	SRP110813	SAMN07136645	SRR5788377	15076646	SRZ187368	metaSPAdes v3.9.0
S0171	GEOTRACES	GA02	PE321	23	2010-06-16T00:22:00	150	ocean_biome	ocean	water	Atlantic Ocean	28.0905 N 67.5015 W	634355	PRJNA385854	SRP110813	SAMN07136646	SRR5788378	21835852	SRZ187369	metaSPAdes v3.9.0
S0172	GEOTRACES	GA02	PE321	23	2010-06-16T00:22:00	200	ocean_biome	ocean	water	Atlantic Ocean	28.0905 N 67.5015 W	634352	PRJNA385854	SRP110813	SAMN07136647	SRR5788379	23183011	SRZ187370	metaSPAdes v3.9.0
S0177	GEOTRACES	GA02	JC057	8	2011-03-13T13:43:00	49	ocean_biome	ocean	water	Atlantic Ocean	35.0093 S 39.431 W	637412	PRJNA385854	SRP110813	SAMN07136648	SRR5788380	48743675	SRZ187371	metaSPAdes v3.9.0
S0178	GEOTRACES	GA02	JC057	8	2011-03-13T13:43:00	151	ocean_biome	ocean	water	Atlantic Ocean	35.0093 S 39.431 W	637403	PRJNA385854	SRP110813	SAMN07136649	SRR5788381	27087463	SRZ187372	metaSPAdes v3.9.0
S0179	GEOTRACES	GA02	JC057	9	2011-03-14T18:45:00	10	ocean_biome	ocean	water	Atlantic Ocean	32.092 S 37.4563 W	639548	PRJNA385854	SRP110813	SAMN07136650	SRR5788382	27291600	SRZ187373	metaSPAdes v3.9.0
S0180	GEOTRACES	GA02	JC057	9	2011-03-14T18:45:00	149	ocean_biome	ocean	water	Atlantic Ocean	32.092 S 37.4563 W	639533	PRJNA385854	SRP110813	SAMN07136651	SRR5788383	32512613	SRZ187374	metaSPAdes v3.9.0
S0181	GEOTRACES	GA02	JC057	10	2011-03-15T23:40:00	11	ocean_biome	ocean	water	Atlantic Ocean	29.0538 S 35.8078 W	639692	PRJNA385854	SRP110813	SAMN07136652	SRR5788384	27483914	SRZ187375	metaSPAdes v3.9.0
S0182	GEOTRACES	GA02	JC057	10	2011-03-15T23:40:00	50	ocean_biome	ocean	water	Atlantic Ocean	29.0538 S 35.8078 W	639686	PRJNA385854	SRP110813	SAMN07136653	SRR5788024	24654021	SRZ187376	metaSPAdes v3.9.0
S0183	GEOTRACES	GA02	JC057	10	2011-03-15T23:40:00	150	ocean_biome	ocean	water	Atlantic Ocean	29.0538 S 35.8078 W	639677	PRJNA385854	SRP110813	SAMN07136654	SRR5788023	21896245	SRZ187377	metaSPAdes v3.9.0
S0184	GEOTRACES	GA02	JC057	11	2011-03-17T00:34:00	10	ocean_biome	ocean	water	Atlantic Ocean	26.0867 S 34.2585 W	639764	PRJNA385854	SRP110813	SAMN07136655	SRR5788022	26689064	SRZ187378	metaSPAdes v3.9.0
S0185	GEOTRACES	GA02	JC057	11	2011-03-17T00:34:00	50	ocean_biome	ocean	water	Atlantic Ocean	26.0867 S 34.2585 W	639758	PRJNA385854	SRP110813	SAMN07136656	SRR5788021	26859299	SRZ187379	metaSPAdes v3.9.0
S0186	GEOTRACES	GA02	JC057	11	2011-03-17T00:34:00	151	ocean_biome	ocean	water	Atlantic Ocean	26.0867 S 34.2585 W	639749	PRJNA385854	SRP110813	SAMN07136657	SRR5788020	31208500	SRZ187380	metaSPAdes v3.9.0
S0187	GEOTRACES	GA02	JC057	12	2011-03-18T14:16:00	9	ocean_biome	ocean	water	Atlantic Ocean	22.4732 S 32.7487 W	638351	PRJNA385854	SRP110813	SAMN07136658	SRR5788019	41426131	SRZ187381	metaSPAdes v3.9.0
S0188	GEOTRACES	GA02	JC057	12	2011-03-18T14:16:00	49	ocean_biome	ocean	water	Atlantic Ocean	22.4732 S 32.7487 W	638345	PRJNA385854	SRP110813	SAMN07136659	SRR5788018	39189883	SRZ187382	metaSPAdes v3.9.0
S0189	GEOTRACES	GA02	JC057	12	2011-03-18T14:16:00	150	ocean_biome	ocean	water	Atlantic Ocean	22.4732 S 32.7487 W	638336	PRJNA385854	SRP110813	SAMN07136660	SRR5788017	29302170	SRZ187383	metaSPAdes v3.9.0
S0190	GEOTRACES	GA02	JC057	13	2011-03-20T14:54:00	49	ocean_biome	ocean	water	Atlantic Ocean	17.0173 S 30.6052 W	638561	PRJNA385854	SRP110813	SAMN07136661	SRR5788016	27855556	SRZ187384	metaSPAdes v3.9.0
S0191	GEOTRACES	GA02	JC057	14	2011-03-22T01:00:00	10	ocean_biome	ocean	water	Atlantic Ocean	12.8957 S 29.2335 W	638711	PRJNA385854	SRP110813	SAMN07136662	SRR5788015	23901114	SRZ187385	metaSPAdes v3.9.0
S0192	GEOTRACES	GA02	JC057	14	2011-03-22T01:00:00	49	ocean_biome	ocean	water	Atlantic Ocean	12.8957 S 29.2335 W	638705	PRJNA385854	SRP110813	SAMN07136663	SRR5788188	29059113	SRZ187386	metaSPAdes v3.9.0
S0193	GEOTRACES	GA02	JC057	14	2011-03-22T01:00:00	149	ocean_biome	ocean	water	Atlantic Ocean	12.8957 S 29.2335 W	638696	PRJNA385854	SRP110813	SAMN07136664	SRR5788189	23175848	SRZ187387	metaSPAdes v3.9.0
S0195	GEOTRACES	GA02	JC057	13	2011-03-20T14:54:00	143	ocean_biome	ocean	water	Atlantic Ocean	17.0173 S 30.6052 W	638552	PRJNA385854	SRP110813	SAMN07136665	SRR5788186	19642047	SRZ187388	metaSPAdes v3.9.0
S0196	GEOTRACES	GA02	JC057	15	2011-03-23T11:11:00	10	ocean_biome	ocean	water	Atlantic Ocean	9.1608 S 28.0012 W	638855	PRJNA385854	SRP110813	SAMN07136666	SRR5788187	19005470	SRZ187389	metaSPAdes v3.9.0
S0197	GEOTRACES	GA02	JC057	15	2011-03-23T11:11:00	50	ocean_biome	ocean	water	Atlantic Ocean	9.1608 S 28.0012 W	638849	PRJNA385854	SRP110813	SAMN07136667	SRR5788184	21337418	SRZ187390	metaSPAdes v3.9.0
S0198	GEOTRACES	GA02	JC057	15	2011-03-23T11:11:00	135	ocean_biome	ocean	water	Atlantic Ocean	9.1608 S 28.0012 W	638840	PRJNA385854	SRP110813	SAMN07136668	SRR5788185	22755372	SRZ187391	metaSPAdes v3.9.0
S0199	GEOTRACES	GA02	JC057	16	2011-03-24T12:54:00	10	ocean_biome	ocean	water	Atlantic Ocean	5.6667 S 28.4572 W	638927	PRJNA385854	SRP110813	SAMN07136669	SRR5788182	27931995	SRZ187392	metaSPAdes v3.9.0
S0200	GEOTRACES	GA02	JC057	16	2011-03-24T12:54:00	50	ocean_biome	ocean	water	Atlantic Ocean	5.6667 S 28.4572 W	638921	PRJNA385854	SRP110813	SAMN07136670	SRR5788183	30978820	SRZ187393	metaSPAdes v3.9.0
S0201	GEOTRACES	GA02	JC057	16	2011-03-24T12:54:00	149	ocean_biome	ocean	water	Atlantic Ocean	5.6667 S 28.4572 W	638912	PRJNA385854	SRP110813	SAMN07136671	SRR5788180	17222947	SRZ187394	metaSPAdes v3.9.0
S0202	GEOTRACES	GA02	JC057	17	2011-03-25T21:08:00	10	ocean_biome	ocean	water	Atlantic Ocean	2.6505 S 28.9172 W	639206	PRJNA385854	SRP110813	SAMN07136672	SRR5788181	19471955	SRZ187395	metaSPAdes v3.9.0
S0203	GEOTRACES	GA02	JC057	17	2011-03-25T21:08:00	65	ocean_biome	ocean	water	Atlantic Ocean	2.6505 S 28.9172 W	639200	PRJNA385854	SRP110813	SAMN07136673	SRR5788352	22439138	SRZ187396	metaSPAdes v3.9.0
S0204	GEOTRACES	GA02	JC057	17	2011-03-25T21:08:00	149	ocean_biome	ocean	water	Atlantic Ocean	2.6505 S 28.9172 W	639191	PRJNA385854	SRP110813	SAMN07136674	SRR5788351	25137503	SRZ187397	metaSPAdes v3.9.0
S0205	GEOTRACES	GA02	JC057	18	2011-03-27T13:11:00	9	ocean_biome	ocean	water	Atlantic Ocean	0.1915 S 32.8745 W	639422	PRJNA385854	SRP110813	SAMN07136675	SRR5788354	17786685	SRZ187398	metaSPAdes v3.9.0
S0206	GEOTRACES	GA02	JC057	18	2011-03-27T13:11:00	61	ocean_biome	ocean	water	Atlantic Ocean	0.1915 S 32.8745 W	639416	PRJNA385854	SRP110813	SAMN07136676	SRR5788353	23722341	SRZ187399	metaSPAdes v3.9.0
S0207	GEOTRACES	GA02	JC057	18	2011-03-27T13:11:00	150	ocean_biome	ocean	water	Atlantic Ocean	0.1915 S 32.8745 W	639407	PRJNA385854	SRP110813	SAMN07136677	SRR5788348	21383021	SRZ187400	metaSPAdes v3.9.0
S0209	GEOTRACES	GA02	JC057	1	2011-03-05T20:56:00	10	ocean_biome	ocean	water	Atlantic Ocean	49.5475 S 52.6885 W	637580	PRJNA385854	SRP110813	SAMN07136678	SRR5788347	19573073	SRZ187401	metaSPAdes v3.9.0
S0210	GEOTRACES	GA02	JC057	1	2011-03-05T20:56:00	49	ocean_biome	ocean	water	Atlantic Ocean	49.5475 S 52.6885 W	637574	PRJNA385854	SRP110813	SAMN07136679	SRR5788350	30987812	SRZ187402	metaSPAdes v3.9.0
S0211	GEOTRACES	GA02	JC057	2	2011-03-06T19:40:00	11	ocean_biome	ocean	water	Atlantic Ocean	48.9358 S 48.7933 W	637724	PRJNA385854	SRP110813	SAMN07136680	SRR5788349	25669096	SRZ187403	metaSPAdes v3.9.0
S0212	GEOTRACES	GA02	JC057	2	2011-03-06T19:40:00	51	ocean_biome	ocean	water	Atlantic Ocean	48.9358 S 48.7933 W	637718	PRJNA385854	SRP110813	SAMN07136681	SRR5788346	28654274	SRZ187404	metaSPAdes v3.9.0
S0213	GEOTRACES	GA02	JC057	2	2011-03-06T19:40:00	150	ocean_biome	ocean	water	Atlantic Ocean	48.9358 S 48.7933 W	637709	PRJNA385854	SRP110813	SAMN07136682	SRR5788345	21335937	SRZ187405	metaSPAdes v3.9.0
S0214	GEOTRACES	GA02	JC057	3	2011-03-08T03:21:00	10	ocean_biome	ocean	water	Atlantic Ocean	46.907 S 47.179 W	638012	PRJNA385854	SRP110813	SAMN07136683	SRR5788104	24326587	SRZ187406	metaSPAdes v3.9.0
S0215	GEOTRACES	GA02	JC057	3	2011-03-08T03:21:00	51	ocean_biome	ocean	water	Atlantic Ocean	46.907 S 47.179 W	638006	PRJNA385854	SRP110813	SAMN07136684	SRR5788103	22973602	SRZ187407	metaSPAdes v3.9.0
S0216	GEOTRACES	GA02	JC057	3	2011-03-08T03:21:00	151	ocean_biome	ocean	water	Atlantic Ocean	46.907 S 47.179 W	637997	PRJNA385854	SRP110813	SAMN07136685	SRR5788106	20401030	SRZ187408	metaSPAdes v3.9.0
S0217	GEOTRACES	GA02	JC057	4	2011-03-09T03:22:00	10	ocean_biome	ocean	water	Atlantic Ocean	44.7117 S 45.5265 W	638156	PRJNA385854	SRP110813	SAMN07136686	SRR5788105	24739783	SRZ187409	metaSPAdes v3.9.0
S0218	GEOTRACES	GA02	JC057	4	2011-03-09T03:22:00	51	ocean_biome	ocean	water	Atlantic Ocean	44.7117 S 45.5265 W	638150	PRJNA385854	SRP110813	SAMN07136687	SRR5788100	24077900	SRZ187410	metaSPAdes v3.9.0
S0219	GEOTRACES	GA02	JC057	4	2011-03-09T03:22:00	149	ocean_biome	ocean	water	Atlantic Ocean	44.7117 S 45.5265 W	638141	PRJNA385854	SRP110813	SAMN07136688	SRR5788099	22869731	SRZ187411	metaSPAdes v3.9.0
S0220	GEOTRACES	GA02	JC057	5	2011-03-10T02:43:00	10	ocean_biome	ocean	water	Atlantic Ocean	42.364 S 44.0318 W	638300	PRJNA385854	SRP110813	SAMN07136689	SRR5788060	25044040	SRZ187412	metaSPAdes v3.9.0
S0221	GEOTRACES	GA02	JC057	5	2011-03-10T02:43:00	50	ocean_biome	ocean	water	Atlantic Ocean	42.364 S 44.0318 W	638294	PRJNA385854	SRP110813	SAMN07136690	SRR5788059	20689131	SRZ187413	metaSPAdes v3.9.0
S0222	GEOTRACES	GA02	JC057	5	2011-03-10T02:43:00	150	ocean_biome	ocean	water	Atlantic Ocean	42.364 S 44.0318 W	638285	PRJNA385854	SRP110813	SAMN07136691	SRR5788048	18166045	SRZ187414	metaSPAdes v3.9.0
S0223	GEOTRACES	GA02	JC057	6	2011-03-11T02:17:00	10	ocean_biome	ocean	water	Atlantic Ocean	39.9648 S 42.4232 W	636995	PRJNA385854	SRP110813	SAMN07136692	SRR5788047	27158330	SRZ187415	metaSPAdes v3.9.0
S0224	GEOTRACES	GA02	JC057	6	2011-03-11T02:17:00	50	ocean_biome	ocean	water	Atlantic Ocean	39.9648 S 42.4232 W	636989	PRJNA385854	SRP110813	SAMN07136693	SRR5788190	19325678	SRZ187416	metaSPAdes v3.9.0
S0225	GEOTRACES	GA02	JC057	6	2011-03-11T02:17:00	149	ocean_biome	ocean	water	Atlantic Ocean	39.9648 S 42.4232 W	636980	PRJNA385854	SRP110813	SAMN07136694	SRR5787992	34436503	SRZ187417	metaSPAdes v3.9.0
S0226	GEOTRACES	GA02	JC057	7	2011-03-12T01:56:00	9	ocean_biome	ocean	water	Atlantic Ocean	37.8305 S 41.1248 W	637139	PRJNA385854	SRP110813	SAMN07136695	SRR5787989	22895492	SRZ187418	metaSPAdes v3.9.0
S0227	GEOTRACES	GA02	JC057	7	2011-03-12T01:56:00	50	ocean_biome	ocean	water	Atlantic Ocean	37.8305 S 41.1248 W	637133	PRJNA385854	SRP110813	SAMN07136696	SRR5787990	18691363	SRZ187419	metaSPAdes v3.9.0
S0228	GEOTRACES	GA02	JC057	7	2011-03-12T01:56:00	148	ocean_biome	ocean	water	Atlantic Ocean	37.8305 S 41.1248 W	637124	PRJNA385854	SRP110813	SAMN07136697	SRR5788467	18234842	SRZ187420	metaSPAdes v3.9.0
S0229	GEOTRACES	GA02	JC057	8	2011-03-13T13:43:00	11	ocean_biome	ocean	water	Atlantic Ocean	35.0093 S 39.431 W	637418	PRJNA385854	SRP110813	SAMN07136698	SRR5788468	23750415	SRZ187421	metaSPAdes v3.9.0
S0231	GEOTRACES	GA03	KN199-4	7	2010-10-24T11:14:00	33.2	ocean_biome	ocean	water	Atlantic Ocean	24.00011 N 22.00011 W	841889	PRJNA385854	SRP110813	SAMN07136699	SRR5788465	37402264	SRZ187422	metaSPAdes v3.9.0
S0232	GEOTRACES	GA03	KN199-4	7	2010-10-24T11:14:00	57.7	ocean_biome	ocean	water	Atlantic Ocean	24.00011 N 22.00011 W	841892	PRJNA385854	SRP110813	SAMN07136700	SRR5788466	22328431	SRZ187423	metaSPAdes v3.9.0
S0233	GEOTRACES	GA03	KN199-4	7	2010-10-24T11:14:00	72.9	ocean_biome	ocean	water	Atlantic Ocean	24.00011 N 22.00011 W	841895	PRJNA385854	SRP110813	SAMN07136701	SRR5788463	17924691	SRZ187424	metaSPAdes v3.9.0
S0234	GEOTRACES	GA03	KN199-4	7	2010-10-24T11:14:00	151.8	ocean_biome	ocean	water	Atlantic Ocean	24.00011 N 22.00011 W	841898	PRJNA385854	SRP110813	SAMN07136702	SRR5788464	19340187	SRZ187425	metaSPAdes v3.9.0
S0235	GEOTRACES	GA03	KN199-4	7	2010-10-24T11:14:00	201.1	ocean_biome	ocean	water	Atlantic Ocean	24.00011 N 22.00011 W	841901	PRJNA385854	SRP110813	SAMN07136703	SRR5788338	18637764	SRZ187426	metaSPAdes v3.9.0
S0236	GEOTRACES	GA03	KN199-4	7	2010-10-24T11:14:00	349	ocean_biome	ocean	water	Atlantic Ocean	24.00011 N 22.00011 W	841904	PRJNA385854	SRP110813	SAMN07136704	SRR5788337	20019777	SRZ187427	metaSPAdes v3.9.0
S0237	GEOTRACES	GA03	KN199-4	7	2010-10-24T09:46:00	2	ocean_biome	ocean	water	Atlantic Ocean	24 N 22.0001 W	853052	PRJNA385854	SRP110813	SAMN07136705	SRR5788336	27875862	SRZ187428	metaSPAdes v3.9.0
S0238	GEOTRACES	GA03	KN199-4	10	2010-10-30T03:06:00	51.8	ocean_biome	ocean	water	Atlantic Ocean	17.35162 N 20.81672 W	842069	PRJNA385854	SRP110813	SAMN07136706	SRR5788335	23482108	SRZ187429	metaSPAdes v3.9.0
S0239	GEOTRACES	GA03	KN199-4	10	2010-10-30T03:06:00	86.6	ocean_biome	ocean	water	Atlantic Ocean	17.35162 N 20.81672 W	842072	PRJNA385854	SRP110813	SAMN07136707	SRR5788342	30959166	SRZ187430	metaSPAdes v3.9.0
S0240	GEOTRACES	GA03	KN199-4	10	2010-10-30T03:06:00	101	ocean_biome	ocean	water	Atlantic Ocean	17.35162 N 20.81672 W	842075	PRJNA385854	SRP110813	SAMN07136708	SRR5788341	41247247	SRZ187431	metaSPAdes v3.9.0
S0241	GEOTRACES	GA03	KN199-4	10	2010-10-30T03:06:00	140.7	ocean_biome	ocean	water	Atlantic Ocean	17.35162 N 20.81672 W	842078	PRJNA385854	SRP110813	SAMN07136709	SRR5788340	20753177	SRZ187432	metaSPAdes v3.9.0
S0242	GEOTRACES	GA03	KN199-4	10	2010-10-30T03:06:00	185	ocean_biome	ocean	water	Atlantic Ocean	17.35162 N 20.81672 W	842081	PRJNA385854	SRP110813	SAMN07136710	SRR5788339	26443918	SRZ187433	metaSPAdes v3.9.0
S0243	GEOTRACES	GA03	KN199-4	10	2010-10-30T00:58:00	2	ocean_biome	ocean	water	Atlantic Ocean	17.3487 N 20.7835 W	853163	PRJNA385854	SRP110813	SAMN07136711	SRR5788344	39074236	SRZ187434	metaSPAdes v3.9.0
S0244	GEOTRACES	GA03	KN199-4	12	2010-11-02T09:05:00	31.8	ocean_biome	ocean	water	Atlantic Ocean	17.39978 N 24.4998 W	842801	PRJNA385854	SRP110813	SAMN07136712	SRR5788343	41023797	SRZ187435	metaSPAdes v3.9.0
S0245	GEOTRACES	GA03	KN199-4	12	2010-11-02T09:05:00	50.4	ocean_biome	ocean	water	Atlantic Ocean	17.39978 N 24.4998 W	842804	PRJNA385854	SRP110813	SAMN07136713	SRR5788172	22851056	SRZ187436	metaSPAdes v3.9.0
S0246	GEOTRACES	GA03	KN199-4	12	2010-11-02T09:05:00	71.9	ocean_biome	ocean	water	Atlantic Ocean	17.39978 N 24.4998 W	842807	PRJNA385854	SRP110813	SAMN07136714	SRR5788173	21782561	SRZ187437	metaSPAdes v3.9.0
S0247	GEOTRACES	GA03	KN199-4	12	2010-11-02T09:05:00	91.6	ocean_biome	ocean	water	Atlantic Ocean	17.39978 N 24.4998 W	842810	PRJNA385854	SRP110813	SAMN07136715	SRR5788174	24677667	SRZ187438	metaSPAdes v3.9.0
S0248	GEOTRACES	GA03	KN199-4	12	2010-11-02T09:05:00	136.5	ocean_biome	ocean	water	Atlantic Ocean	17.39978 N 24.4998 W	842813	PRJNA385854	SRP110813	SAMN07136716	SRR5788175	21813258	SRZ187439	metaSPAdes v3.9.0
S0249	GEOTRACES	GA03	KN199-4	12	2010-11-02T09:05:00	185.4	ocean_biome	ocean	water	Atlantic Ocean	17.39978 N 24.4998 W	842816	PRJNA385854	SRP110813	SAMN07136717	SRR5788168	21576284	SRZ187440	metaSPAdes v3.9.0
S0250	GEOTRACES	GA03	KN199-4	12	2010-11-02T07:57:00	2	ocean_biome	ocean	water	Atlantic Ocean	17.4 N 24.4998 W	853064	PRJNA385854	SRP110813	SAMN07136718	SRR5788169	22125940	SRZ187441	metaSPAdes v3.9.0
S0252	GEOTRACES	GP13	TAN1109	GT14	2011-06-17T01:54:00	101	ocean_biome	ocean	water	Pacific Ocean	32.46367 S 159.01933 W	1155512	PRJNA385854	SRP110813	SAMN07136719	SRR5788170	21390525	SRZ187442	metaSPAdes v3.9.0
S0253	GEOTRACES	GP13	TAN1109	GT15	2011-06-17T14:06:00	14	ocean_biome	ocean	water	Pacific Ocean	32.495 S 157.9945 W	1155698	PRJNA385854	SRP110813	SAMN07136720	SRR5788171	31801807	SRZ187443	metaSPAdes v3.9.0
S0254	GEOTRACES	GP13	TAN1109	GT15	2011-06-17T14:06:00	31	ocean_biome	ocean	water	Pacific Ocean	32.495 S 157.9945 W	1155674	PRJNA385854	SRP110813	SAMN07136721	SRR5788176	27682186	SRZ187444	metaSPAdes v3.9.0
S0255	GEOTRACES	GP13	TAN1109	GT15	2011-06-17T14:06:00	51	ocean_biome	ocean	water	Pacific Ocean	32.495 S 157.9945 W	1155668	PRJNA385854	SRP110813	SAMN07136722	SRR5788177	20325731	SRZ187445	metaSPAdes v3.9.0
S0256	GEOTRACES	GP13	TAN1109	GT15	2011-06-17T14:06:00	76	ocean_biome	ocean	water	Pacific Ocean	32.495 S 157.9945 W	1155662	PRJNA385854	SRP110813	SAMN07136723	SRR5788149	25464364	SRZ187446	metaSPAdes v3.9.0
S0257	GEOTRACES	GP13	TAN1109	GT15	2011-06-17T14:06:00	100	ocean_biome	ocean	water	Pacific Ocean	32.495 S 157.9945 W	1155656	PRJNA385854	SRP110813	SAMN07136724	SRR5788148	28544146	SRZ187447	metaSPAdes v3.9.0
S0258	GEOTRACES	GP13	TAN1109	GT15	2011-06-17T14:06:00	202	ocean_biome	ocean	water	Pacific Ocean	32.495 S 157.9945 W	1155638	PRJNA385854	SRP110813	SAMN07136725	SRR5788151	24710896	SRZ187448	metaSPAdes v3.9.0
S0259	GEOTRACES	GP13	TAN1109	GT15	2011-06-17T20:11:00	1008	ocean_biome	ocean	water	Pacific Ocean	32.5125 S 158.028 W	1155833	PRJNA385854	SRP110813	SAMN07136726	SRR5788150	18318834	SRZ187449	metaSPAdes v3.9.0
S0260	GEOTRACES	GP13	TAN1109	GT15	2011-06-17T20:11:00	5601	ocean_biome	ocean	water	Pacific Ocean	32.5125 S 158.028 W	1155776	PRJNA385854	SRP110813	SAMN07136727	SRR5788153	25320424	SRZ187450	metaSPAdes v3.9.0
S0261	GEOTRACES	GP13	TAN1109	GT16	2011-06-18T10:34:00	15	ocean_biome	ocean	water	Pacific Ocean	32.494 S 157.00883 W	1155905	PRJNA385854	SRP110813	SAMN07136728	SRR5788152	27186550	SRZ187451	metaSPAdes v3.9.0
S0262	GEOTRACES	GP13	TAN1109	GT16	2011-06-18T10:34:00	30	ocean_biome	ocean	water	Pacific Ocean	32.494 S 157.00883 W	1155878	PRJNA385854	SRP110813	SAMN07136729	SRR5788155	28456586	SRZ187452	metaSPAdes v3.9.0
S0263	GEOTRACES	GP13	SS2011	7	2011-05-17T19:55:38	15.5	ocean_biome	ocean	water	Pacific Ocean	29.9999 S 159 E	1223669	PRJNA385854	SRP110813	SAMN07136730	SRR5788154	32873273	SRZ187453	metaSPAdes v3.9.0
S0264	GEOTRACES	GP13	SS2011	7	2011-05-17T19:55:38	30	ocean_biome	ocean	water	Pacific Ocean	29.9999 S 159 E	1223666	PRJNA385854	SRP110813	SAMN07136731	SRR5788157	29833436	SRZ187454	metaSPAdes v3.9.0
S0265	GEOTRACES	GP13	SS2011	7	2011-05-17T19:55:38	50.6	ocean_biome	ocean	water	Pacific Ocean	29.9999 S 159 E	1223663	PRJNA385854	SRP110813	SAMN07136732	SRR5788156	23120115	SRZ187455	metaSPAdes v3.9.0
S0266	GEOTRACES	GP13	SS2011	7	2011-05-17T19:55:38	78	ocean_biome	ocean	water	Pacific Ocean	29.9999 S 159 E	1222871	PRJNA385854	SRP110813	SAMN07136733	SRR5788039	26592876	SRZ187456	metaSPAdes v3.9.0
S0267	GEOTRACES	GP13	SS2011	7	2011-05-17T19:55:38	101	ocean_biome	ocean	water	Pacific Ocean	29.9999 S 159 E	1223687	PRJNA385854	SRP110813	SAMN07136734	SRR5788040	28032741	SRZ187457	metaSPAdes v3.9.0
S0268	GEOTRACES	GP13	SS2011	11	2011-05-19T15:42:38	16.1	ocean_biome	ocean	water	Pacific Ocean	30.0006 S 162.998 E	1223804	PRJNA385854	SRP110813	SAMN07136735	SRR5788037	22009497	SRZ187458	metaSPAdes v3.9.0
S0269	GEOTRACES	GP13	SS2011	11	2011-05-19T15:42:38	29.9	ocean_biome	ocean	water	Pacific Ocean	30.0006 S 162.998 E	1223798	PRJNA385854	SRP110813	SAMN07136736	SRR5788038	31907021	SRZ187459	metaSPAdes v3.9.0
S0270	GEOTRACES	GP13	SS2011	11	2011-05-19T15:42:38	51.3	ocean_biome	ocean	water	Pacific Ocean	30.0006 S 162.998 E	1223792	PRJNA385854	SRP110813	SAMN07136737	SRR5788043	28012015	SRZ187460	metaSPAdes v3.9.0
S0271	GEOTRACES	GP13	SS2011	11	2011-05-19T15:42:38	74	ocean_biome	ocean	water	Pacific Ocean	30.0006 S 162.998 E	1223834	PRJNA385854	SRP110813	SAMN07136738	SRR5788044	28695563	SRZ187461	metaSPAdes v3.9.0
S0272	GEOTRACES	GP13	SS2011	11	2011-05-19T15:42:38	100.8	ocean_biome	ocean	water	Pacific Ocean	30.0006 S 162.998 E	1223831	PRJNA385854	SRP110813	SAMN07136739	SRR5788041	16979826	SRZ187462	metaSPAdes v3.9.0
S0273	GEOTRACES	GP13	SS2011	15	2011-05-21T16:06:42	13.8	ocean_biome	ocean	water	Pacific Ocean	30 S 166.987 E	1223984	PRJNA385854	SRP110813	SAMN07136740	SRR5788042	17796661	SRZ187463	metaSPAdes v3.9.0
S0274	GEOTRACES	GP13	SS2011	15	2011-05-21T16:06:42	30.8	ocean_biome	ocean	water	Pacific Ocean	30 S 166.987 E	1223981	PRJNA385854	SRP110813	SAMN07136741	SRR5788045	17218320	SRZ187464	metaSPAdes v3.9.0
S0275	GEOTRACES	GP13	SS2011	15	2011-05-21T16:06:42	51.1	ocean_biome	ocean	water	Pacific Ocean	30 S 166.987 E	1223978	PRJNA385854	SRP110813	SAMN07136742	SRR5788046	15195243	SRZ187465	metaSPAdes v3.9.0
S0276	GEOTRACES	GP13	SS2011	15	2011-05-21T16:06:42	74.8	ocean_biome	ocean	water	Pacific Ocean	30 S 166.987 E	1224011	PRJNA385854	SRP110813	SAMN07136743	SRR5788403	16871150	SRZ187466	metaSPAdes v3.9.0
S0277	GEOTRACES	GP13	SS2011	15	2011-05-21T16:06:42	101.7	ocean_biome	ocean	water	Pacific Ocean	30 S 166.987 E	1224008	PRJNA385854	SRP110813	SAMN07136744	SRR5788402	24646835	SRZ187467	metaSPAdes v3.9.0
S0278	GEOTRACES	GP13	SS2011	19	2011-05-23T11:54:38	15.1	ocean_biome	ocean	water	Pacific Ocean	29.9997 S 171 E	1222262	PRJNA385854	SRP110813	SAMN07136745	SRR5788401	14444871	SRZ187468	metaSPAdes v3.9.0
S0279	GEOTRACES	GP13	SS2011	19	2011-05-23T11:54:38	30.7	ocean_biome	ocean	water	Pacific Ocean	29.9997 S 171 E	1222256	PRJNA385854	SRP110813	SAMN07136746	SRR5788400	18301023	SRZ187469	metaSPAdes v3.9.0
S0280	GEOTRACES	GP13	SS2011	19	2011-05-23T11:54:38	50.4	ocean_biome	ocean	water	Pacific Ocean	29.9997 S 171 E	1222250	PRJNA385854	SRP110813	SAMN07136747	SRR5788399	20428536	SRZ187470	metaSPAdes v3.9.0
S0281	GEOTRACES	GP13	SS2011	19	2011-05-23T11:54:38	75.4	ocean_biome	ocean	water	Pacific Ocean	29.9997 S 171 E	1222292	PRJNA385854	SRP110813	SAMN07136748	SRR5788398	18354078	SRZ187471	metaSPAdes v3.9.0
S0282	GEOTRACES	GP13	SS2011	19	2011-05-23T11:54:38	100.5	ocean_biome	ocean	water	Pacific Ocean	29.9997 S 171 E	1222289	PRJNA385854	SRP110813	SAMN07136749	SRR5788397	23614957	SRZ187472	metaSPAdes v3.9.0
S0283	GEOTRACES	GP13	SS2011	32	2011-05-29T16:18:17	14.9	ocean_biome	ocean	water	Pacific Ocean	32.4995 S 176.03 W	1224224	PRJNA385854	SRP110813	SAMN07136750	SRR5788396	19195872	SRZ187473	metaSPAdes v3.9.0
S0284	GEOTRACES	GP13	SS2011	32	2011-05-29T16:18:17	29.3	ocean_biome	ocean	water	Pacific Ocean	32.4995 S 176.03 W	1223207	PRJNA385854	SRP110813	SAMN07136751	SRR5788406	21830840	SRZ187474	metaSPAdes v3.9.0
S0285	GEOTRACES	GP13	SS2011	32	2011-05-29T16:18:17	48.9	ocean_biome	ocean	water	Pacific Ocean	32.4995 S 176.03 W	1223204	PRJNA385854	SRP110813	SAMN07136752	SRR5788405	20628269	SRZ187475	metaSPAdes v3.9.0
S0286	GEOTRACES	GP13	SS2011	32	2011-05-29T16:18:17	76.8	ocean_biome	ocean	water	Pacific Ocean	32.4995 S 176.03 W	1224218	PRJNA385854	SRP110813	SAMN07136753	SRR5788301	26041646	SRZ187476	metaSPAdes v3.9.0
S0287	GEOTRACES	GP13	SS2011	32	2011-05-29T16:18:17	100.3	ocean_biome	ocean	water	Pacific Ocean	32.4995 S 176.03 W	1223198	PRJNA385854	SRP110813	SAMN07136754	SRR5788404	21887350	SRZ187477	metaSPAdes v3.9.0
S0288	GEOTRACES	GP13	SS2011	32	2011-05-29T16:18:17	999.9	ocean_biome	ocean	water	Pacific Ocean	32.4995 S 176.03 W	1223225	PRJNA385854	SRP110813	SAMN07136755	SRR5788265	66585301	SRZ187478	metaSPAdes v3.10.1
S0289	GEOTRACES	GP13	SS2011	4	2011-05-16T17:59:17	74.5	ocean_biome	ocean	water	Pacific Ocean	30.0006 S 155.98599 E	1222811	PRJNA385854	SRP110813	SAMN07136756	SRR5788266	23038780	SRZ187479	metaSPAdes v3.9.0
S0290	GEOTRACES	GP13	SS2011	4	2011-05-16T17:59:17	100.9	ocean_biome	ocean	water	Pacific Ocean	30.0006 S 155.98599 E	1223579	PRJNA385854	SRP110813	SAMN07136757	SRR5788267	19232368	SRZ187480	metaSPAdes v3.9.0
S0291	GEOTRACES	GP13	SS2011	22	2011-05-24T15:11:38	14.9	ocean_biome	ocean	water	Pacific Ocean	30.0013 S 174.00101 E	1222409	PRJNA385854	SRP110813	SAMN07136758	SRR5788268	24564664	SRZ187481	metaSPAdes v3.9.0
S0292	GEOTRACES	GP13	SS2011	22	2011-05-24T15:11:38	30.1	ocean_biome	ocean	water	Pacific Ocean	30.0013 S 174.00101 E	1222406	PRJNA385854	SRP110813	SAMN07136759	SRR5788269	25093955	SRZ187482	metaSPAdes v3.9.0
S0293	GEOTRACES	GP13	SS2011	22	2011-05-24T15:11:38	50.4	ocean_biome	ocean	water	Pacific Ocean	30.0013 S 174.00101 E	1222400	PRJNA385854	SRP110813	SAMN07136760	SRR5788270	25005859	SRZ187483	metaSPAdes v3.9.0
S0294	GEOTRACES	GA02	PE319	18	2010-05-22T13:07:00	9	ocean_biome	ocean	water	Atlantic Ocean	33.4334 N 58.0503 W	632273	PRJNA385854	SRP110813	SAMN07136761	SRR5788387	37800395	SRZ187484	metaSPAdes v3.9.0
S0295	GEOTRACES	GA02	PE319	18	2010-05-22T13:07:00	51	ocean_biome	ocean	water	Atlantic Ocean	33.4334 N 58.0503 W	632267	PRJNA385854	SRP110813	SAMN07136762	SRR5788256	28286252	SRZ187485	metaSPAdes v3.9.0
S0296	GEOTRACES	GA02	PE319	18	2010-05-22T13:07:00	76	ocean_biome	ocean	water	Atlantic Ocean	33.4334 N 58.0503 W	632264	PRJNA385854	SRP110813	SAMN07136763	SRR5788000	19821300	SRZ187486	metaSPAdes v3.9.0
S0297	GEOTRACES	GA02	PE319	18	2010-05-22T13:07:00	151	ocean_biome	ocean	water	Atlantic Ocean	33.4334 N 58.0503 W	632258	PRJNA385854	SRP110813	SAMN07136764	SRR5787999	24052904	SRZ187487	metaSPAdes v3.9.0
S0298	GEOTRACES	GA02	PE319	18	2010-05-22T13:07:00	201	ocean_biome	ocean	water	Atlantic Ocean	33.4334 N 58.0503 W	632255	PRJNA385854	SRP110813	SAMN07136765	SRR5788002	16699984	SRZ187488	metaSPAdes v3.9.0
S0299	GEOTRACES	GP13	SS2011	1	2011-05-14T10:37:58	15	ocean_biome	ocean	water	Pacific Ocean	29.9982 S 153.50101 E	1223384	PRJNA385854	SRP110813	SAMN07136766	SRR5788001	24108811	SRZ187489	metaSPAdes v3.9.0
S0300	GEOTRACES	GP13	SS2011	1	2011-05-14T10:37:58	49.8	ocean_biome	ocean	water	Pacific Ocean	29.9982 S 153.50101 E	1223423	PRJNA385854	SRP110813	SAMN07136767	SRR5787996	26856634	SRZ187490	metaSPAdes v3.9.0
S0301	GEOTRACES	GP13	SS2011	2	2011-05-15T01:28:38	15.5	ocean_biome	ocean	water	Pacific Ocean	30.0019 S 154.004 E	1223459	PRJNA385854	SRP110813	SAMN07136768	SRR5787995	19481133	SRZ187491	metaSPAdes v3.9.0
S0302	GEOTRACES	GP13	SS2011	2	2011-05-15T01:28:38	30.8	ocean_biome	ocean	water	Pacific Ocean	30.0019 S 154.004 E	1223453	PRJNA385854	SRP110813	SAMN07136769	SRR5787998	24252995	SRZ187492	metaSPAdes v3.9.0
S0303	GEOTRACES	GP13	SS2011	2	2011-05-15T01:28:38	51.7	ocean_biome	ocean	water	Pacific Ocean	30.0019 S 154.004 E	1223447	PRJNA385854	SRP110813	SAMN07136770	SRR5787997	22850699	SRZ187493	metaSPAdes v3.9.0
S0304	GEOTRACES	GP13	SS2011	2	2011-05-15T01:28:38	75.2	ocean_biome	ocean	water	Pacific Ocean	30.0019 S 154.004 E	1223486	PRJNA385854	SRP110813	SAMN07136771	SRR5788004	19058795	SRZ187494	metaSPAdes v3.9.0
S0305	GEOTRACES	GP13	SS2011	2	2011-05-15T01:28:38	99	ocean_biome	ocean	water	Pacific Ocean	30.0019 S 154.004 E	1223483	PRJNA385854	SRP110813	SAMN07136772	SRR5788003	22365162	SRZ187495	metaSPAdes v3.9.0
S0306	GEOTRACES	GP13	SS2011	4	2011-05-16T17:59:17	15.4	ocean_biome	ocean	water	Pacific Ocean	30.0006 S 155.98599 E	1223552	PRJNA385854	SRP110813	SAMN07136773	SRR5788361	23708877	SRZ187496	metaSPAdes v3.9.0
S0307	GEOTRACES	GP13	SS2011	4	2011-05-16T17:59:17	30.9	ocean_biome	ocean	water	Pacific Ocean	30.0006 S 155.98599 E	1222814	PRJNA385854	SRP110813	SAMN07136774	SRR5788362	41123843	SRZ187497	metaSPAdes v3.9.0
S0308	GEOTRACES	GP13	SS2011	4	2011-05-16T17:59:17	50.6	ocean_biome	ocean	water	Pacific Ocean	30.0006 S 155.98599 E	1223543	PRJNA385854	SRP110813	SAMN07136775	SRR5788359	21699729	SRZ187498	metaSPAdes v3.9.0
S0309	GEOTRACES	GP13	SS2011	22	2011-05-24T15:11:38	75.1	ocean_biome	ocean	water	Pacific Ocean	30.0013 S 174.00101 E	1222397	PRJNA385854	SRP110813	SAMN07136776	SRR5788360	19692083	SRZ187499	metaSPAdes v3.9.0
S0310	GEOTRACES	GP13	SS2011	22	2011-05-24T15:11:38	100.4	ocean_biome	ocean	water	Pacific Ocean	30.0013 S 174.00101 E	1222430	PRJNA385854	SRP110813	SAMN07136777	SRR5788357	21519848	SRZ187500	metaSPAdes v3.9.0
S0311	GEOTRACES	GP13	SS2011	24	2011-05-25T19:16:06	15.6	ocean_biome	ocean	water	Pacific Ocean	30.0002 S 175.98 E	1222499	PRJNA385854	SRP110813	SAMN07136778	SRR5788358	20660085	SRZ187501	metaSPAdes v3.9.0
S0312	GEOTRACES	GP13	SS2011	24	2011-05-25T19:16:06	30.6	ocean_biome	ocean	water	Pacific Ocean	30.0002 S 175.98 E	1222496	PRJNA385854	SRP110813	SAMN07136779	SRR5788355	18269141	SRZ187502	metaSPAdes v3.9.0
S0313	GEOTRACES	GP13	SS2011	24	2011-05-25T19:16:06	50.3	ocean_biome	ocean	water	Pacific Ocean	30.0002 S 175.98 E	1222490	PRJNA385854	SRP110813	SAMN07136780	SRR5788356	13911489	SRZ187503	metaSPAdes v3.9.0
S0314	GEOTRACES	GP13	SS2011	24	2011-05-25T19:16:06	74.5	ocean_biome	ocean	water	Pacific Ocean	30.0002 S 175.98 E	1222487	PRJNA385854	SRP110813	SAMN07136781	SRR5788363	15002166	SRZ187504	metaSPAdes v3.9.0
S0320	GEOTRACES	GP13	SS2011	38	2011-05-31T18:10:31	100.3	ocean_biome	ocean	water	Pacific Ocean	32.5001 S 170 W	1223297	PRJNA385854	SRP110813	SAMN07136787	SRR5788271	29330937	SRZ187510	metaSPAdes v3.9.0
S0321	GEOTRACES	GP13	SS2011	38	2011-05-31T18:10:31	1001.5	ocean_biome	ocean	water	Pacific Ocean	32.5001 S 170 W	1222625	PRJNA385854	SRP110813	SAMN07136788	SRR5788272	13772733	SRZ187511	metaSPAdes v3.9.0
S0322	GEOTRACES	GP13	SS2011	36	2011-06-01T12:02:53	15.1	ocean_biome	ocean	water	Pacific Ocean	32.4998 S 171.99899 W	1222715	PRJNA385854	SRP110813	SAMN07136789	SRR5788273	18400054	SRZ187512	metaSPAdes v3.9.0
S0323	GEOTRACES	GP13	SS2011	36	2011-06-01T12:02:53	30.9	ocean_biome	ocean	water	Pacific Ocean	32.4998 S 171.99899 W	1222706	PRJNA385854	SRP110813	SAMN07136790	SRR5788274	49526662	SRZ187513	metaSPAdes v3.10.1
S0324	GEOTRACES	GP13	SS2011	36	2011-06-01T12:02:53	49.8	ocean_biome	ocean	water	Pacific Ocean	32.4998 S 171.99899 W	1222700	PRJNA385854	SRP110813	SAMN07136791	SRR5788279	13229663	SRZ187514	metaSPAdes v3.9.0
S0325	GEOTRACES	GP13	SS2011	36	2011-06-01T12:02:53	75.1	ocean_biome	ocean	water	Pacific Ocean	32.4998 S 171.99899 W	1222742	PRJNA385854	SRP110813	SAMN07136792	SRR5788280	14210122	SRZ187515	metaSPAdes v3.9.0
S0326	GEOTRACES	GP13	SS2011	36	2011-06-01T12:02:53	100.3	ocean_biome	ocean	water	Pacific Ocean	32.4998 S 171.99899 W	1222739	PRJNA385854	SRP110813	SAMN07136793	SRR5788418	16550153	SRZ187516	metaSPAdes v3.9.0
S0331	GEOTRACES	GP13	TAN1109	GT8	2011-06-13T22:40:00	50	ocean_biome	ocean	water	Pacific Ocean	32.49567 S 164.99233 W	1154516	PRJNA385854	SRP110813	SAMN07136794	SRR5788417	35104271	SRZ187517	metaSPAdes v3.9.0
S0332	GEOTRACES	GP13	TAN1109	GT8	2011-06-13T22:40:00	75	ocean_biome	ocean	water	Pacific Ocean	32.49567 S 164.99233 W	1154510	PRJNA385854	SRP110813	SAMN07136795	SRR5788416	18683493	SRZ187518	metaSPAdes v3.9.0
S0333	GEOTRACES	GP13	TAN1109	GT8	2011-06-13T22:40:00	100	ocean_biome	ocean	water	Pacific Ocean	32.49567 S 164.99233 W	1154504	PRJNA385854	SRP110813	SAMN07136796	SRR5788415	19783515	SRZ187519	metaSPAdes v3.9.0
S0334	GEOTRACES	GP13	TAN1109	GT8	2011-06-13T22:40:00	204	ocean_biome	ocean	water	Pacific Ocean	32.49567 S 164.99233 W	1154486	PRJNA385854	SRP110813	SAMN07136797	SRR5788422	31611680	SRZ187520	metaSPAdes v3.9.0
S0335	GEOTRACES	GP13	TAN1109	GT8	2011-06-14T04:11:00	1023	ocean_biome	ocean	water	Pacific Ocean	32.515 S 164.98117 W	1152842	PRJNA385854	SRP110813	SAMN07136798	SRR5788421	23640245	SRZ187521	metaSPAdes v3.9.0
S0336	GEOTRACES	GP13	TAN1109	GT8	2011-06-14T04:11:00	5100	ocean_biome	ocean	water	Pacific Ocean	32.515 S 164.98117 W	1152779	PRJNA385854	SRP110813	SAMN07136799	SRR5788420	26910641	SRZ187522	metaSPAdes v3.9.0
S0337	GEOTRACES	GP13	TAN1109	GT9	2011-06-14T16:46:00	17	ocean_biome	ocean	water	Pacific Ocean	32.50433 S 163.98967 W	1152896	PRJNA385854	SRP110813	SAMN07136800	SRR5788419	29257699	SRZ187523	metaSPAdes v3.9.0
S0338	GEOTRACES	GP13	TAN1109	GT9	2011-06-14T16:46:00	31	ocean_biome	ocean	water	Pacific Ocean	32.50433 S 163.98967 W	1152887	PRJNA385854	SRP110813	SAMN07136801	SRR5788424	39878937	SRZ187524	metaSPAdes v3.9.0
S0339	GEOTRACES	GP13	TAN1109	GT9	2011-06-14T16:46:00	51	ocean_biome	ocean	water	Pacific Ocean	32.50433 S 163.98967 W	1152881	PRJNA385854	SRP110813	SAMN07136802	SRR5788423	24751268	SRZ187525	metaSPAdes v3.9.0
S0340	GEOTRACES	GP13	TAN1109	GT9	2011-06-14T16:46:00	76	ocean_biome	ocean	water	Pacific Ocean	32.50433 S 163.98967 W	1152875	PRJNA385854	SRP110813	SAMN07136803	SRR5788057	26157822	SRZ187526	metaSPAdes v3.9.0
S0341	GEOTRACES	GP13	TAN1109	GT9	2011-06-14T16:46:00	102	ocean_biome	ocean	water	Pacific Ocean	32.50433 S 163.98967 W	1152866	PRJNA385854	SRP110813	SAMN07136804	SRR5788058	22082858	SRZ187527	metaSPAdes v3.9.0
S0342	GEOTRACES	GP13	TAN1109	GT16	2011-06-18T10:34:00	49	ocean_biome	ocean	water	Pacific Ocean	32.494 S 157.00883 W	1155872	PRJNA385854	SRP110813	SAMN07136805	SRR5788055	21046094	SRZ187528	metaSPAdes v3.9.0
S0343	GEOTRACES	GP13	TAN1109	GT16	2011-06-18T10:34:00	76	ocean_biome	ocean	water	Pacific Ocean	32.494 S 157.00883 W	1155866	PRJNA385854	SRP110813	SAMN07136806	SRR5788056	23393374	SRZ187529	metaSPAdes v3.9.0
S0344	GEOTRACES	GP13	TAN1109	GT16	2011-06-18T10:34:00	102	ocean_biome	ocean	water	Pacific Ocean	32.494 S 157.00883 W	1155860	PRJNA385854	SRP110813	SAMN07136807	SRR5788053	20135065	SRZ187530	metaSPAdes v3.9.0
S0345	GEOTRACES	GP13	TAN1109	GT17	2011-06-18T20:24:00	15	ocean_biome	ocean	water	Pacific Ocean	32.50383 S 156.01067 W	1156046	PRJNA385854	SRP110813	SAMN07136808	SRR5788054	24557138	SRZ187531	metaSPAdes v3.9.0
S0346	GEOTRACES	GP13	TAN1109	GT17	2011-06-18T20:24:00	31	ocean_biome	ocean	water	Pacific Ocean	32.50383 S 156.01067 W	1156019	PRJNA385854	SRP110813	SAMN07136809	SRR5788051	57253336	SRZ187532	metaSPAdes v3.9.0
S0347	GEOTRACES	GP13	TAN1109	GT17	2011-06-18T20:24:00	52	ocean_biome	ocean	water	Pacific Ocean	32.50383 S 156.01067 W	1156013	PRJNA385854	SRP110813	SAMN07136810	SRR5788052	24848456	SRZ187533	metaSPAdes v3.9.0
S0348	GEOTRACES	GP13	TAN1109	GT17	2011-06-18T20:24:00	77	ocean_biome	ocean	water	Pacific Ocean	32.50383 S 156.01067 W	1156007	PRJNA385854	SRP110813	SAMN07136811	SRR5788049	22413635	SRZ187534	metaSPAdes v3.9.0
S0349	GEOTRACES	GP13	TAN1109	GT17	2011-06-18T20:24:00	102	ocean_biome	ocean	water	Pacific Ocean	32.50383 S 156.01067 W	1156001	PRJNA385854	SRP110813	SAMN07136812	SRR5788050	23286308	SRZ187535	metaSPAdes v3.9.0
S0350	GEOTRACES	GP13	TAN1109	GT18	2011-06-19T06:47:00	14	ocean_biome	ocean	water	Pacific Ocean	32.4995 S 154.99933 W	1156181	PRJNA385854	SRP110813	SAMN07136813	SRR5788165	19681955	SRZ187536	metaSPAdes v3.9.0
S0351	GEOTRACES	GP13	TAN1109	GT18	2011-06-19T06:47:00	30	ocean_biome	ocean	water	Pacific Ocean	32.4995 S 154.99933 W	1156163	PRJNA385854	SRP110813	SAMN07136814	SRR5788164	32325722	SRZ187537	metaSPAdes v3.9.0
S0352	GEOTRACES	GP13	TAN1109	GT18	2011-06-19T06:47:00	51	ocean_biome	ocean	water	Pacific Ocean	32.4995 S 154.99933 W	1156157	PRJNA385854	SRP110813	SAMN07136815	SRR5788167	49007326	SRZ187538	metaSPAdes v3.10.1
S0353	GEOTRACES	GP13	TAN1109	GT18	2011-06-19T06:47:00	74	ocean_biome	ocean	water	Pacific Ocean	32.4995 S 154.99933 W	1156151	PRJNA385854	SRP110813	SAMN07136816	SRR5788166	34522597	SRZ187539	metaSPAdes v3.9.0
S0354	GEOTRACES	GP13	TAN1109	GT18	2011-06-19T06:47:00	101	ocean_biome	ocean	water	Pacific Ocean	32.4995 S 154.99933 W	1156145	PRJNA385854	SRP110813	SAMN07136817	SRR5788161	45891951	SRZ187540	metaSPAdes v3.9.0
S0355	GEOTRACES	GP13	TAN1109	GT19	2011-06-19T18:28:00	15	ocean_biome	ocean	water	Pacific Ocean	32.50133 S 153.99817 W	1156325	PRJNA385854	SRP110813	SAMN07136818	SRR5788160	50071414	SRZ187541	metaSPAdes v3.9.0
S0356	GEOTRACES	GP13	TAN1109	GT19	2011-06-19T18:28:00	32	ocean_biome	ocean	water	Pacific Ocean	32.50133 S 153.99817 W	1156301	PRJNA385854	SRP110813	SAMN07136819	SRR5788163	31995419	SRZ187542	metaSPAdes v3.9.0
S0357	GEOTRACES	GP13	TAN1109	GT19	2011-06-19T18:28:00	51	ocean_biome	ocean	water	Pacific Ocean	32.50133 S 153.99817 W	1156295	PRJNA385854	SRP110813	SAMN07136820	SRR5788162	40428965	SRZ187543	metaSPAdes v3.9.0
S0358	GEOTRACES	GP13	TAN1109	GT19	2011-06-19T18:28:00	77	ocean_biome	ocean	water	Pacific Ocean	32.50133 S 153.99817 W	1156289	PRJNA385854	SRP110813	SAMN07136821	SRR5788159	46879404	SRZ187544	metaSPAdes v3.9.0
S0359	GEOTRACES	GP13	TAN1109	GT19	2011-06-19T18:28:00	100	ocean_biome	ocean	water	Pacific Ocean	32.50133 S 153.99817 W	1156283	PRJNA385854	SRP110813	SAMN07136822	SRR5788158	28005890	SRZ187545	metaSPAdes v3.9.0
S0360	GEOTRACES	GP13	TAN1109	GT19	2011-06-19T18:28:00	201	ocean_biome	ocean	water	Pacific Ocean	32.50133 S 153.99817 W	1156265	PRJNA385854	SRP110813	SAMN07136823	SRR5788327	14940894	SRZ187546	metaSPAdes v3.9.0
S0361	GEOTRACES	GP13	TAN1109	GT19	2011-06-19T22:18:00	1008	ocean_biome	ocean	water	Pacific Ocean	32.49483 S 154.00417 W	1156466	PRJNA385854	SRP110813	SAMN07136824	SRR5788328	32602172	SRZ187547	metaSPAdes v3.9.0
S0362	GEOTRACES	GP13	TAN1109	GT19	2011-06-19T22:18:00	5095	ocean_biome	ocean	water	Pacific Ocean	32.49483 S 154.00417 W	1156412	PRJNA385854	SRP110813	SAMN07136825	SRR5788329	20836757	SRZ187548	metaSPAdes v3.9.0
S0363	GEOTRACES	GP13	TAN1109	GT20	2011-06-20T13:01:00	15	ocean_biome	ocean	water	Pacific Ocean	32.494 S 153.00567 W	1156538	PRJNA385854	SRP110813	SAMN07136826	SRR5788330	16750427	SRZ187549	metaSPAdes v3.9.0
S0365	GEOTRACES	GP13	TAN1109	GT5	2011-06-12T16:59:00	75	ocean_biome	ocean	water	Pacific Ocean	32.495 S 168.002 W	1154141	PRJNA385854	SRP110813	SAMN07136827	SRR5788331	23589892	SRZ187550	metaSPAdes v3.9.0
S0366	GEOTRACES	GP13	TAN1109	GT5	2011-06-12T16:59:00	101	ocean_biome	ocean	water	Pacific Ocean	32.495 S 168.002 W	1154138	PRJNA385854	SRP110813	SAMN07136828	SRR5788332	22450261	SRZ187551	metaSPAdes v3.9.0
S0367	GEOTRACES	GP13	TAN1109	GT6	2011-06-13T03:37:00	14	ocean_biome	ocean	water	Pacific Ocean	32.49717 S 167.00217 W	1154327	PRJNA385854	SRP110813	SAMN07136829	SRR5788333	23218521	SRZ187552	metaSPAdes v3.9.0
S0368	GEOTRACES	GP13	TAN1109	GT6	2011-06-13T03:37:00	31	ocean_biome	ocean	water	Pacific Ocean	32.49717 S 167.00217 W	1154300	PRJNA385854	SRP110813	SAMN07136830	SRR5788334	24366634	SRZ187553	metaSPAdes v3.9.0
S0369	GEOTRACES	GP13	TAN1109	GT6	2011-06-13T03:37:00	52	ocean_biome	ocean	water	Pacific Ocean	32.49717 S 167.00217 W	1154294	PRJNA385854	SRP110813	SAMN07136831	SRR5788325	18396289	SRZ187554	metaSPAdes v3.9.0
S0370	GEOTRACES	GP13	TAN1109	GT6	2011-06-13T03:37:00	78	ocean_biome	ocean	water	Pacific Ocean	32.49717 S 167.00217 W	1154288	PRJNA385854	SRP110813	SAMN07136832	SRR5788326	22968413	SRZ187555	metaSPAdes v3.9.0
S0371	GEOTRACES	GP13	TAN1109	GT6	2011-06-13T03:37:00	102	ocean_biome	ocean	water	Pacific Ocean	32.49717 S 167.00217 W	1154282	PRJNA385854	SRP110813	SAMN07136833	SRR5788179	26069956	SRZ187556	metaSPAdes v3.9.0
S0372	GEOTRACES	GP13	TAN1109	GT7	2011-06-13T12:49:00	16	ocean_biome	ocean	water	Pacific Ocean	32.4985 S 165.99883 W	1154399	PRJNA385854	SRP110813	SAMN07136834	SRR5788178	20672936	SRZ187557	metaSPAdes v3.9.0
S0373	GEOTRACES	GP13	TAN1109	GT7	2011-06-13T12:49:00	30	ocean_biome	ocean	water	Pacific Ocean	32.4985 S 165.99883 W	1154372	PRJNA385854	SRP110813	SAMN07136835	SRR5788462	27975409	SRZ187558	metaSPAdes v3.9.0
S0374	GEOTRACES	GP13	TAN1109	GT7	2011-06-13T12:49:00	48	ocean_biome	ocean	water	Pacific Ocean	32.4985 S 165.99883 W	1154366	PRJNA385854	SRP110813	SAMN07136836	SRR5788461	28820623	SRZ187559	metaSPAdes v3.9.0
S0375	GEOTRACES	GP13	TAN1109	GT7	2011-06-13T12:49:00	76	ocean_biome	ocean	water	Pacific Ocean	32.4985 S 165.99883 W	1154360	PRJNA385854	SRP110813	SAMN07136837	SRR5788460	30318403	SRZ187560	metaSPAdes v3.9.0
S0376	GEOTRACES	GP13	TAN1109	GT7	2011-06-13T12:49:00	102	ocean_biome	ocean	water	Pacific Ocean	32.4985 S 165.99883 W	1154354	PRJNA385854	SRP110813	SAMN07136838	SRR5788459	25360404	SRZ187561	metaSPAdes v3.9.0
S0377	GEOTRACES	GP13	TAN1109	GT8	2011-06-13T22:40:00	15	ocean_biome	ocean	water	Pacific Ocean	32.49567 S 164.99233 W	1152704	PRJNA385854	SRP110813	SAMN07136839	SRR5788458	23713880	SRZ187562	metaSPAdes v3.9.0
S0378	GEOTRACES	GP13	TAN1109	GT8	2011-06-13T22:40:00	28	ocean_biome	ocean	water	Pacific Ocean	32.49567 S 164.99233 W	1154522	PRJNA385854	SRP110813	SAMN07136840	SRR5788457	26096985	SRZ187563	metaSPAdes v3.9.0
S0379	GEOTRACES	GP13	TAN1109	GT10	2011-06-15T11:03:00	15	ocean_biome	ocean	water	Pacific Ocean	32.50633 S 162.9935 W	1153013	PRJNA385854	SRP110813	SAMN07136841	SRR5788191	37613607	SRZ187564	metaSPAdes v3.9.0
S0380	GEOTRACES	GP13	TAN1109	GT10	2011-06-15T11:03:00	33	ocean_biome	ocean	water	Pacific Ocean	32.50633 S 162.9935 W	1153007	PRJNA385854	SRP110813	SAMN07136842	SRR5787991	21828256	SRZ187565	metaSPAdes v3.9.0
S0381	GEOTRACES	GP13	TAN1109	GT10	2011-06-15T11:03:00	50	ocean_biome	ocean	water	Pacific Ocean	32.50633 S 162.9935 W	1153001	PRJNA385854	SRP110813	SAMN07136843	SRR5788093	25280632	SRZ187566	metaSPAdes v3.9.0
S0382	GEOTRACES	GP13	TAN1109	GT10	2011-06-15T11:03:00	75	ocean_biome	ocean	water	Pacific Ocean	32.50633 S 162.9935 W	1152995	PRJNA385854	SRP110813	SAMN07136844	SRR5788094	26513334	SRZ187567	metaSPAdes v3.9.0
S0383	GEOTRACES	GP13	TAN1109	GT10	2011-06-15T11:03:00	102	ocean_biome	ocean	water	Pacific Ocean	32.50633 S 162.9935 W	1152989	PRJNA385854	SRP110813	SAMN07136845	SRR5788091	24852584	SRZ187568	metaSPAdes v3.9.0
S0384	GEOTRACES	GP13	TAN1109	GT11	2011-06-15T21:47:00	16	ocean_biome	ocean	water	Pacific Ocean	32.509 S 162.009 W	1153175	PRJNA385854	SRP110813	SAMN07136846	SRR5788092	25882482	SRZ187569	metaSPAdes v3.9.0
S0385	GEOTRACES	GP13	TAN1109	GT11	2011-06-15T21:47:00	30	ocean_biome	ocean	water	Pacific Ocean	32.509 S 162.009 W	1153148	PRJNA385854	SRP110813	SAMN07136847	SRR5788097	23154518	SRZ187570	metaSPAdes v3.9.0
S0386	GEOTRACES	GP13	TAN1109	GT11	2011-06-15T21:47:00	52	ocean_biome	ocean	water	Pacific Ocean	32.509 S 162.009 W	1153142	PRJNA385854	SRP110813	SAMN07136848	SRR5788098	38570180	SRZ187571	metaSPAdes v3.9.0
S0387	GEOTRACES	GP13	TAN1109	GT11	2011-06-15T21:47:00	76	ocean_biome	ocean	water	Pacific Ocean	32.509 S 162.009 W	1153136	PRJNA385854	SRP110813	SAMN07136849	SRR5788095	20334266	SRZ187572	metaSPAdes v3.9.0
S0388	GEOTRACES	GP13	TAN1109	GT11	2011-06-15T21:47:00	101	ocean_biome	ocean	water	Pacific Ocean	32.509 S 162.009 W	1153130	PRJNA385854	SRP110813	SAMN07136850	SRR5788096	17863516	SRZ187573	metaSPAdes v3.9.0
S0389	GEOTRACES	GP13	TAN1109	GT12	2011-06-16T08:38:00	15	ocean_biome	ocean	water	Pacific Ocean	32.49783 S 160.99283 W	1153316	PRJNA385854	SRP110813	SAMN07136851	SRR5788101	35290609	SRZ187574	metaSPAdes v3.9.0
S0390	GEOTRACES	GP13	TAN1109	GT12	2011-06-16T08:38:00	30	ocean_biome	ocean	water	Pacific Ocean	32.49783 S 160.99283 W	1153289	PRJNA385854	SRP110813	SAMN07136852	SRR5788102	25738308	SRZ187575	metaSPAdes v3.9.0
S0391	GEOTRACES	GP13	TAN1109	GT12	2011-06-16T08:38:00	50	ocean_biome	ocean	water	Pacific Ocean	32.49783 S 160.99283 W	1153283	PRJNA385854	SRP110813	SAMN07136853	SRR5788324	21444337	SRZ187576	metaSPAdes v3.9.0
S0392	GEOTRACES	GP13	TAN1109	GT12	2011-06-16T08:38:00	73	ocean_biome	ocean	water	Pacific Ocean	32.49783 S 160.99283 W	1153277	PRJNA385854	SRP110813	SAMN07136854	SRR5788255	27930115	SRZ187577	metaSPAdes v3.9.0
S0393	GEOTRACES	GP13	TAN1109	GT12	2011-06-16T08:38:00	100	ocean_biome	ocean	water	Pacific Ocean	32.49783 S 160.99283 W	1153271	PRJNA385854	SRP110813	SAMN07136855	SRR5788258	23006446	SRZ187578	metaSPAdes v3.9.0
S0394	GEOTRACES	GP13	TAN1109	GT13	2011-06-16T16:16:00	15	ocean_biome	ocean	water	Pacific Ocean	32.51033 S 160.00783 W	1155413	PRJNA385854	SRP110813	SAMN07136856	SRR5788257	9194168	SRZ187579	metaSPAdes v3.9.0
S0395	GEOTRACES	GP13	TAN1109	GT13	2011-06-16T16:16:00	32	ocean_biome	ocean	water	Pacific Ocean	32.51033 S 160.00783 W	1155410	PRJNA385854	SRP110813	SAMN07136857	SRR5788260	12126318	SRZ187580	metaSPAdes v3.9.0
S0396	GEOTRACES	GP13	TAN1109	GT13	2011-06-16T16:16:00	49	ocean_biome	ocean	water	Pacific Ocean	32.51033 S 160.00783 W	1155404	PRJNA385854	SRP110813	SAMN07136858	SRR5788259	13822451	SRZ187581	metaSPAdes v3.9.0
S0397	GEOTRACES	GP13	TAN1109	GT13	2011-06-16T16:16:00	72	ocean_biome	ocean	water	Pacific Ocean	32.51033 S 160.00783 W	1155398	PRJNA385854	SRP110813	SAMN07136859	SRR5788262	23080374	SRZ187582	metaSPAdes v3.9.0
S0398	GEOTRACES	GP13	TAN1109	GT13	2011-06-16T16:16:00	102	ocean_biome	ocean	water	Pacific Ocean	32.51033 S 160.00783 W	1155392	PRJNA385854	SRP110813	SAMN07136860	SRR5788261	21240431	SRZ187583	metaSPAdes v3.9.0
S0399	GEOTRACES	GP13	TAN1109	GT14	2011-06-17T01:54:00	15	ocean_biome	ocean	water	Pacific Ocean	32.46367 S 159.01933 W	1155560	PRJNA385854	SRP110813	SAMN07136861	SRR5788264	15298157	SRZ187584	metaSPAdes v3.9.0
S0400	GEOTRACES	GP13	TAN1109	GT14	2011-06-17T01:54:00	32	ocean_biome	ocean	water	Pacific Ocean	32.46367 S 159.01933 W	1155533	PRJNA385854	SRP110813	SAMN07136862	SRR5788263	26481316	SRZ187585	metaSPAdes v3.9.0
S0401	GEOTRACES	GP13	TAN1109	GT14	2011-06-17T01:54:00	49	ocean_biome	ocean	water	Pacific Ocean	32.46367 S 159.01933 W	1155527	PRJNA385854	SRP110813	SAMN07136863	SRR5788124	16706385	SRZ187586	metaSPAdes v3.9.0
S0402	GEOTRACES	GP13	TAN1109	GT14	2011-06-17T01:54:00	76	ocean_biome	ocean	water	Pacific Ocean	32.46367 S 159.01933 W	1155521	PRJNA385854	SRP110813	SAMN07136864	SRR5788125	22209104	SRZ187587	metaSPAdes v3.9.0
S0408	GEOTRACES	GP13	TAN1109	GT21	2011-06-21T00:03:00	52	ocean_biome	ocean	water	Pacific Ocean	32.4885 S 151.9995 W	1156649	PRJNA385854	SRP110813	SAMN07136870	SRR5788123	18548063	SRZ187593	metaSPAdes v3.9.0
S0409	GEOTRACES	GP13	TAN1109	GT21	2011-06-21T00:03:00	75	ocean_biome	ocean	water	Pacific Ocean	32.4885 S 151.9995 W	1156643	PRJNA385854	SRP110813	SAMN07136871	SRR5788118	17325652	SRZ187594	metaSPAdes v3.9.0
S0410	GEOTRACES	GP13	TAN1109	GT21	2011-06-21T00:03:00	102	ocean_biome	ocean	water	Pacific Ocean	32.4885 S 151.9995 W	1156637	PRJNA385854	SRP110813	SAMN07136872	SRR5788119	21384181	SRZ187595	metaSPAdes v3.9.0
S0411	GEOTRACES	GP13	TAN1109	GT22	2011-06-21T13:10:00	15	ocean_biome	ocean	water	Pacific Ocean	32.49567 S 151.00067 W	1156883	PRJNA385854	SRP110813	SAMN07136873	SRR5788296	16822336	SRZ187596	metaSPAdes v3.9.0
S0412	GEOTRACES	GP13	TAN1109	GT22	2011-06-21T13:10:00	30	ocean_biome	ocean	water	Pacific Ocean	32.49567 S 151.00067 W	1156871	PRJNA385854	SRP110813	SAMN07136874	SRR5788295	34831503	SRZ187597	metaSPAdes v3.9.0
S0413	GEOTRACES	GP13	TAN1109	GT22	2011-06-21T13:10:00	50	ocean_biome	ocean	water	Pacific Ocean	32.49567 S 151.00067 W	1156865	PRJNA385854	SRP110813	SAMN07136875	SRR5788294	26513020	SRZ187598	metaSPAdes v3.9.0
S0414	GEOTRACES	GP13	TAN1109	GT22	2011-06-21T13:10:00	76	ocean_biome	ocean	water	Pacific Ocean	32.49567 S 151.00067 W	1156859	PRJNA385854	SRP110813	SAMN07136876	SRR5788293	18990041	SRZ187599	metaSPAdes v3.9.0
S0415	GEOTRACES	GP13	SS2011	26	2011-05-26T22:16:59	15.2	ocean_biome	ocean	water	Pacific Ocean	31.5988 S 178 E	1222577	PRJNA385854	SRP110813	SAMN07136877	SRR5788300	17695124	SRZ187600	metaSPAdes v3.9.0
S0416	GEOTRACES	GP13	SS2011	26	2011-05-26T22:16:59	30	ocean_biome	ocean	water	Pacific Ocean	31.5988 S 178 E	1222574	PRJNA385854	SRP110813	SAMN07136878	SRR5788299	39184164	SRZ187601	metaSPAdes v3.9.0
S0417	GEOTRACES	GP13	SS2011	26	2011-05-26T22:16:59	51.2	ocean_biome	ocean	water	Pacific Ocean	31.5988 S 178 E	1222571	PRJNA385854	SRP110813	SAMN07136879	SRR5788298	26216805	SRZ187602	metaSPAdes v3.9.0
S0418	GEOTRACES	GP13	SS2011	26	2011-05-26T22:16:59	74.1	ocean_biome	ocean	water	Pacific Ocean	31.5988 S 178 E	1222601	PRJNA385854	SRP110813	SAMN07136880	SRR5788297	16368240	SRZ187603	metaSPAdes v3.9.0
S0419	GEOTRACES	GP13	SS2011	26	2011-05-26T22:16:59	99.8	ocean_biome	ocean	water	Pacific Ocean	31.5988 S 178 E	1222598	PRJNA385854	SRP110813	SAMN07136881	SRR5788292	30862080	SRZ187604	metaSPAdes v3.9.0
S0420	GEOTRACES	GP13	SS2011	29	2011-05-28T11:09:40	15.6	ocean_biome	ocean	water	Pacific Ocean	32.4995 S 179.125 W	1223060	PRJNA385854	SRP110813	SAMN07136882	SRR5788291	23704703	SRZ187605	metaSPAdes v3.9.0
S0421	GEOTRACES	GP13	SS2011	29	2011-05-28T11:09:40	29.5	ocean_biome	ocean	water	Pacific Ocean	32.4995 S 179.125 W	1223054	PRJNA385854	SRP110813	SAMN07136883	SRR5788391	24662809	SRZ187606	metaSPAdes v3.9.0
S0422	GEOTRACES	GP13	SS2011	29	2011-05-28T11:09:40	51.1	ocean_biome	ocean	water	Pacific Ocean	32.4995 S 179.125 W	1223048	PRJNA385854	SRP110813	SAMN07136884	SRR5788390	17069017	SRZ187607	metaSPAdes v3.9.0
S0423	GEOTRACES	GP13	SS2011	29	2011-05-28T11:09:40	74.7	ocean_biome	ocean	water	Pacific Ocean	32.4995 S 179.125 W	1223090	PRJNA385854	SRP110813	SAMN07136885	SRR5788389	14988541	SRZ187608	metaSPAdes v3.9.0
S0424	GEOTRACES	GP13	SS2011	29	2011-05-28T11:09:40	99.9	ocean_biome	ocean	water	Pacific Ocean	32.4995 S 179.125 W	1223087	PRJNA385854	SRP110813	SAMN07136886	SRR5788388	17668777	SRZ187609	metaSPAdes v3.9.0
S0425	GEOTRACES	GP13	SS2011	29	2011-05-28T11:09:40	1001.5	ocean_biome	ocean	water	Pacific Ocean	32.4995 S 179.125 W	1223069	PRJNA385854	SRP110813	SAMN07136887	SRR5788395	13647414	SRZ187610	metaSPAdes v3.9.0
S0426	GEOTRACES	GP13	TAN1109	GT2	2011-06-10T22:48:00	16	ocean_biome	ocean	water	Pacific Ocean	32.49667 S 170.99533 W	1153688	PRJNA385854	SRP110813	SAMN07136888	SRR5788394	22076215	SRZ187611	metaSPAdes v3.9.0
S0427	GEOTRACES	GP13	TAN1109	GT2	2011-06-10T22:48:00	32	ocean_biome	ocean	water	Pacific Ocean	32.49667 S 170.99533 W	1153676	PRJNA385854	SRP110813	SAMN07136889	SRR5788393	25248983	SRZ187612	metaSPAdes v3.9.0
S0428	GEOTRACES	GP13	TAN1109	GT2	2011-06-10T22:48:00	52	ocean_biome	ocean	water	Pacific Ocean	32.49667 S 170.99533 W	1153670	PRJNA385854	SRP110813	SAMN07136890	SRR5788392	29743814	SRZ187613	metaSPAdes v3.9.0
S0429	GEOTRACES	GP13	TAN1109	GT2	2011-06-10T22:48:00	75	ocean_biome	ocean	water	Pacific Ocean	32.49667 S 170.99533 W	1153664	PRJNA385854	SRP110813	SAMN07136891	SRR5788386	24480902	SRZ187614	metaSPAdes v3.9.0
S0430	GEOTRACES	GP13	TAN1109	GT2	2011-06-10T22:48:00	103	ocean_biome	ocean	water	Pacific Ocean	32.49667 S 170.99533 W	1153658	PRJNA385854	SRP110813	SAMN07136892	SRR5788385	24061880	SRZ187615	metaSPAdes v3.9.0
S0431	GEOTRACES	GP13	TAN1109	GT3	2011-06-11T08:25:00	16	ocean_biome	ocean	water	Pacific Ocean	32.49367 S 169.99517 W	1153841	PRJNA385854	SRP110813	SAMN07136893	SRR5788245	21158057	SRZ187616	metaSPAdes v3.9.0
S0432	GEOTRACES	GP13	TAN1109	GT3	2011-06-11T08:25:00	32	ocean_biome	ocean	water	Pacific Ocean	32.49367 S 169.99517 W	1153820	PRJNA385854	SRP110813	SAMN07136894	SRR5788246	36725570	SRZ187617	metaSPAdes v3.9.0
S0433	GEOTRACES	GP13	TAN1109	GT3	2011-06-11T08:25:00	51	ocean_biome	ocean	water	Pacific Ocean	32.49367 S 169.99517 W	1153814	PRJNA385854	SRP110813	SAMN07136895	SRR5788247	20941560	SRZ187618	metaSPAdes v3.9.0
S0434	GEOTRACES	GP13	TAN1109	GT3	2011-06-11T08:25:00	75	ocean_biome	ocean	water	Pacific Ocean	32.49367 S 169.99517 W	1153808	PRJNA385854	SRP110813	SAMN07136896	SRR5788248	21290766	SRZ187619	metaSPAdes v3.9.0
S0435	GEOTRACES	GP13	TAN1109	GT3	2011-06-11T08:25:00	103	ocean_biome	ocean	water	Pacific Ocean	32.49367 S 169.99517 W	1153802	PRJNA385854	SRP110813	SAMN07136897	SRR5788241	22211220	SRZ187620	metaSPAdes v3.9.0
S0436	GEOTRACES	GP13	TAN1109	GT3	2011-06-11T08:25:00	203	ocean_biome	ocean	water	Pacific Ocean	32.49367 S 169.99517 W	1153793	PRJNA385854	SRP110813	SAMN07136898	SRR5788242	18685468	SRZ187621	metaSPAdes v3.9.0
S0437	GEOTRACES	GP13	TAN1109	GT3	2011-06-11T14:02:00	1012	ocean_biome	ocean	water	Pacific Ocean	32.4875 S 169.991 W	1153967	PRJNA385854	SRP110813	SAMN07136899	SRR5788243	15479438	SRZ187622	metaSPAdes v3.9.0
S0438	GEOTRACES	GP13	TAN1109	GT3	2011-06-11T14:02:00	4580	ocean_biome	ocean	water	Pacific Ocean	32.4875 S 169.991 W	1153916	PRJNA385854	SRP110813	SAMN07136900	SRR5788244	11990970	SRZ187623	metaSPAdes v3.9.0
S0439	GEOTRACES	GP13	TAN1109	GT4	2011-06-12T07:26:00	17	ocean_biome	ocean	water	Pacific Ocean	32.499 S 169.00233 W	1154021	PRJNA385854	SRP110813	SAMN07136901	SRR5788239	26503549	SRZ187624	metaSPAdes v3.9.0
S0440	GEOTRACES	GP13	TAN1109	GT4	2011-06-12T07:26:00	32	ocean_biome	ocean	water	Pacific Ocean	32.499 S 169.00233 W	1154009	PRJNA385854	SRP110813	SAMN07136902	SRR5788240	15805512	SRZ187625	metaSPAdes v3.9.0
S0441	GEOTRACES	GP13	TAN1109	GT4	2011-06-12T07:26:00	52	ocean_biome	ocean	water	Pacific Ocean	32.499 S 169.00233 W	1154003	PRJNA385854	SRP110813	SAMN07136903	SRR5788133	17282275	SRZ187626	metaSPAdes v3.9.0
S0442	GEOTRACES	GP13	TAN1109	GT4	2011-06-12T07:26:00	76	ocean_biome	ocean	water	Pacific Ocean	32.499 S 169.00233 W	1153997	PRJNA385854	SRP110813	SAMN07136904	SRR5788132	44757684	SRZ187627	metaSPAdes v3.9.0
S0443	GEOTRACES	GP13	TAN1109	GT4	2011-06-12T07:26:00	101	ocean_biome	ocean	water	Pacific Ocean	32.499 S 169.00233 W	1153991	PRJNA385854	SRP110813	SAMN07136905	SRR5788135	19489583	SRZ187628	metaSPAdes v3.9.0
S0444	GEOTRACES	GP13	TAN1109	GT5	2011-06-12T16:59:00	15	ocean_biome	ocean	water	Pacific Ocean	32.495 S 168.002 W	1154183	PRJNA385854	SRP110813	SAMN07136906	SRR5788134	20595351	SRZ187629	metaSPAdes v3.9.0
S0445	GEOTRACES	GP13	TAN1109	GT5	2011-06-12T16:59:00	30	ocean_biome	ocean	water	Pacific Ocean	32.495 S 168.002 W	1154156	PRJNA385854	SRP110813	SAMN07136907	SRR5788129	20855176	SRZ187630	metaSPAdes v3.9.0
S0446	GEOTRACES	GP13	TAN1109	GT5	2011-06-12T16:59:00	50	ocean_biome	ocean	water	Pacific Ocean	32.495 S 168.002 W	1154147	PRJNA385854	SRP110813	SAMN07136908	SRR5788128	17858224	SRZ187631	metaSPAdes v3.9.0
S0447	GEOTRACES	GA10	D357	1	2010-10-19T06:14:00	6.1	ocean_biome	ocean	water	Atlantic Ocean	34.62898 S 17.06995 E	237617	PRJNA385854	SRP110813	SAMN07136909	SRR5788131	20500870	SRZ187632	metaSPAdes v3.9.0
S0448	GEOTRACES	GA10	D357	1	2010-10-19T06:14:00	21.1	ocean_biome	ocean	water	Atlantic Ocean	34.62898 S 17.06995 E	237616	PRJNA385854	SRP110813	SAMN07136910	SRR5788130	23963719	SRZ187633	metaSPAdes v3.9.0
S0449	GEOTRACES	GA10	D357	1	2010-10-19T06:14:00	31.2	ocean_biome	ocean	water	Atlantic Ocean	34.62898 S 17.06995 E	237615	PRJNA385854	SRP110813	SAMN07136911	SRR5788137	17977059	SRZ187634	metaSPAdes v3.9.0
S0450	GEOTRACES	GA10	D357	1	2010-10-19T06:14:00	40.8	ocean_biome	ocean	water	Atlantic Ocean	34.62898 S 17.06995 E	237614	PRJNA385854	SRP110813	SAMN07136912	SRR5788136	12717788	SRZ187635	metaSPAdes v3.9.0
S0451	GEOTRACES	GA10	D357	1	2010-10-19T06:14:00	51.4	ocean_biome	ocean	water	Atlantic Ocean	34.62898 S 17.06995 E	237613	PRJNA385854	SRP110813	SAMN07136913	SRR5788032	21510615	SRZ187636	metaSPAdes v3.9.0
S0452	GEOTRACES	GA10	D357	1	2010-10-19T06:14:00	60.9	ocean_biome	ocean	water	Atlantic Ocean	34.62898 S 17.06995 E	237607	PRJNA385854	SRP110813	SAMN07136914	SRR5788033	24932198	SRZ187637	metaSPAdes v3.9.0
S0453	GEOTRACES	GA10	D357	2	2010-10-20T10:08:00	6.4	ocean_biome	ocean	water	Atlantic Ocean	35.36689 S 14.90645 E	237665	PRJNA385854	SRP110813	SAMN07136915	SRR5788030	37413305	SRZ187638	metaSPAdes v3.9.0
S0454	GEOTRACES	GA10	D357	2	2010-10-20T10:08:00	21.3	ocean_biome	ocean	water	Atlantic Ocean	35.36689 S 14.90645 E	237663	PRJNA385854	SRP110813	SAMN07136916	SRR5788031	30995468	SRZ187639	metaSPAdes v3.10.1
S0455	GEOTRACES	GA10	D357	2	2010-10-20T10:08:00	51.1	ocean_biome	ocean	water	Atlantic Ocean	35.36689 S 14.90645 E	237662	PRJNA385854	SRP110813	SAMN07136917	SRR5788028	28264789	SRZ187640	metaSPAdes v3.9.0
S0456	GEOTRACES	GA10	D357	2	2010-10-20T10:08:00	61.4	ocean_biome	ocean	water	Atlantic Ocean	35.36689 S 14.90645 E	237656	PRJNA385854	SRP110813	SAMN07136918	SRR5788029	18737160	SRZ187641	metaSPAdes v3.9.0
S0457	GEOTRACES	GA10	D357	2	2010-10-20T10:08:00	81.5	ocean_biome	ocean	water	Atlantic Ocean	35.36689 S 14.90645 E	237655	PRJNA385854	SRP110813	SAMN07136919	SRR5788026	33396452	SRZ187642	metaSPAdes v3.9.0
S0458	GEOTRACES	GA10	D357	2	2010-10-20T10:08:00	101.9	ocean_biome	ocean	water	Atlantic Ocean	35.36689 S 14.90645 E	237654	PRJNA385854	SRP110813	SAMN07136920	SRR5788027	38872304	SRZ187643	metaSPAdes v3.9.0
S0459	GEOTRACES	GA10	D357	3	2010-10-23T03:39:00	10	ocean_biome	ocean	water	Atlantic Ocean	36.48458 S 13.276 E	237705	PRJNA385854	SRP110813	SAMN07136921	SRR5788034	35885564	SRZ187644	metaSPAdes v3.9.0
S0460	GEOTRACES	GA10	D357	3	2010-10-23T03:39:00	21.1	ocean_biome	ocean	water	Atlantic Ocean	36.48458 S 13.276 E	237702	PRJNA385854	SRP110813	SAMN07136922	SRR5788035	24432069	SRZ187645	metaSPAdes v3.9.0
S0461	GEOTRACES	GA10	D357	3	2010-10-23T03:39:00	50.2	ocean_biome	ocean	water	Atlantic Ocean	36.48458 S 13.276 E	237700	PRJNA385854	SRP110813	SAMN07136923	SRR5788446	22114386	SRZ187646	metaSPAdes v3.9.0
S0462	GEOTRACES	GA10	D357	3	2010-10-23T03:39:00	59.8	ocean_biome	ocean	water	Atlantic Ocean	36.48458 S 13.276 E	237698	PRJNA385854	SRP110813	SAMN07136924	SRR5788445	21437570	SRZ187647	metaSPAdes v3.9.0
S0463	GEOTRACES	GA10	D357	3	2010-10-23T03:39:00	81.5	ocean_biome	ocean	water	Atlantic Ocean	36.48458 S 13.276 E	237697	PRJNA385854	SRP110813	SAMN07136925	SRR5788444	3030493	SRZ187648	metaSPAdes v3.9.0
S0464	GEOTRACES	GA10	D357	3	2010-10-23T03:39:00	101	ocean_biome	ocean	water	Atlantic Ocean	36.48458 S 13.276 E	237693	PRJNA385854	SRP110813	SAMN07136926	SRR5788443	3340457	SRZ187649	metaSPAdes v3.9.0
S0465	GEOTRACES	GA10	D357	4	2010-10-26T06:22:00	6.5	ocean_biome	ocean	water	Atlantic Ocean	38.42539 S 10.08884 E	237500	PRJNA385854	SRP110813	SAMN07136927	SRR5788442	4023804	SRZ187650	metaSPAdes v3.9.0
S0466	GEOTRACES	GA10	D357	4	2010-10-26T06:22:00	21.4	ocean_biome	ocean	water	Atlantic Ocean	38.42539 S 10.08884 E	237498	PRJNA385854	SRP110813	SAMN07136928	SRR5788441	4073413	SRZ187651	metaSPAdes v3.9.0
S0467	GEOTRACES	GA10	D357	4	2010-10-26T06:22:00	42.2	ocean_biome	ocean	water	Atlantic Ocean	38.42539 S 10.08884 E	237497	PRJNA385854	SRP110813	SAMN07136929	SRR5788440	4614269	SRZ187652	metaSPAdes v3.9.0
S0468	GEOTRACES	GA10	D357	4	2010-10-26T06:22:00	52.1	ocean_biome	ocean	water	Atlantic Ocean	38.42539 S 10.08884 E	237496	PRJNA385854	SRP110813	SAMN07136930	SRR5788439	2621276	SRZ187653	metaSPAdes v3.9.0
S0469	GEOTRACES	GA10	D357	4	2010-10-26T06:22:00	62	ocean_biome	ocean	water	Atlantic Ocean	38.42539 S 10.08884 E	237494	PRJNA385854	SRP110813	SAMN07136931	SRR5788438	4628766	SRZ187654	metaSPAdes v3.9.0
S0470	GEOTRACES	GA10	D357	4	2010-10-26T06:22:00	101.9	ocean_biome	ocean	water	Atlantic Ocean	38.42539 S 10.08884 E	237491	PRJNA385854	SRP110813	SAMN07136932	SRR5788437	8488936	SRZ187655	metaSPAdes v3.9.0
S0471	GEOTRACES	GA10	D357	5	2010-10-27T19:24:00	6.3	ocean_biome	ocean	water	Atlantic Ocean	40.06269 S 5.52721 E	237547	PRJNA385854	SRP110813	SAMN07136933	SRR5788302	4633907	SRZ187656	metaSPAdes v3.9.0
S0472	GEOTRACES	GA10	D357	5	2010-10-27T19:24:00	21.6	ocean_biome	ocean	water	Atlantic Ocean	40.06269 S 5.52721 E	237545	PRJNA385854	SRP110813	SAMN07136934	SRR5788303	37316272	SRZ187657	metaSPAdes v3.9.0
S0473	GEOTRACES	GA10	D357	5	2010-10-27T19:24:00	51.2	ocean_biome	ocean	water	Atlantic Ocean	40.06269 S 5.52721 E	237538	PRJNA385854	SRP110813	SAMN07136935	SRR5788304	31544686	SRZ187658	metaSPAdes v3.9.0
S0474	GEOTRACES	GA10	D357	5	2010-10-27T19:24:00	66.3	ocean_biome	ocean	water	Atlantic Ocean	40.06269 S 5.52721 E	237537	PRJNA385854	SRP110813	SAMN07136936	SRR5788305	136553716	SRZ187659	metaSPAdes v3.9.0
S0475	GEOTRACES	GA10	D357	5	2010-10-27T19:24:00	81.4	ocean_biome	ocean	water	Atlantic Ocean	40.06269 S 5.52721 E	237535	PRJNA385854	SRP110813	SAMN07136937	SRR5788306	179603272	SRZ187660	metaSPAdes v3.9.0
S0476	GEOTRACES	GA10	D357	5	2010-10-27T19:24:00	101.9	ocean_biome	ocean	water	Atlantic Ocean	40.06269 S 5.52721 E	237533	PRJNA385854	SRP110813	SAMN07136938	SRR5788307	63169988	SRZ187661	metaSPAdes v3.9.0
S0477	GEOTRACES	GA10	D357	6	2010-10-29T18:23:00	6	ocean_biome	ocean	water	Atlantic Ocean	40.01935 S 0.88755 E	237745	PRJNA385854	SRP110813	SAMN07136939	SRR5788308	15108191	SRZ187662	metaSPAdes v3.9.0
S0478	GEOTRACES	GA10	D357	6	2010-10-29T18:23:00	21.4	ocean_biome	ocean	water	Atlantic Ocean	40.01935 S 0.88755 E	237743	PRJNA385854	SRP110813	SAMN07136940	SRR5788309	23994618	SRZ187663	metaSPAdes v3.9.0
S0479	GEOTRACES	GA10	D357	6	2010-10-29T18:23:00	50.9	ocean_biome	ocean	water	Atlantic Ocean	40.01935 S 0.88755 E	237741	PRJNA385854	SRP110813	SAMN07136941	SRR5788310	22535725	SRZ187664	metaSPAdes v3.9.0
S0480	GEOTRACES	GA10	D357	6	2010-10-29T18:23:00	61.4	ocean_biome	ocean	water	Atlantic Ocean	40.01935 S 0.88755 E	237739	PRJNA385854	SRP110813	SAMN07136942	SRR5788311	18642753	SRZ187665	metaSPAdes v3.9.0
S0481	GEOTRACES	GA10	D357	6	2010-10-29T18:23:00	76.2	ocean_biome	ocean	water	Atlantic Ocean	40.01935 S 0.88755 E	237737	PRJNA385854	SRP110813	SAMN07136943	SRR5788206	17579682	SRZ187666	metaSPAdes v3.9.0
S0487	GEOTRACES	GA10	D357	9	2010-11-10T12:29:00	71.6	ocean_biome	ocean	water	Atlantic Ocean	34.89531 S 16.07692 E	237829	PRJNA385854	SRP110813	SAMN07136949	SRR5788212	27914646	SRZ187672	metaSPAdes v3.9.0
S0488	GEOTRACES	GA10	D357	9	2010-11-10T12:29:00	101.1	ocean_biome	ocean	water	Atlantic Ocean	34.89531 S 16.07692 E	237826	PRJNA385854	SRP110813	SAMN07136950	SRR5788211	23142791	SRZ187673	metaSPAdes v3.9.0
S0489	GEOTRACES	GA10	D357	3 reoccupied	2010-11-12T07:08:00	6.3	ocean_biome	ocean	water	Atlantic Ocean	36.4495 S 13.21958 E	237889	PRJNA385854	SRP110813	SAMN07136951	SRR5788203	24121575	SRZ187674	metaSPAdes v3.9.0
S0490	GEOTRACES	GA10	D357	3 reoccupied	2010-11-12T07:08:00	21.1	ocean_biome	ocean	water	Atlantic Ocean	36.4495 S 13.21958 E	237887	PRJNA385854	SRP110813	SAMN07136952	SRR5788202	26180530	SRZ187675	metaSPAdes v3.9.0
S0491	GEOTRACES	GA10	D357	3 reoccupied	2010-11-12T07:08:00	40.7	ocean_biome	ocean	water	Atlantic Ocean	36.4495 S 13.21958 E	237886	PRJNA385854	SRP110813	SAMN07136953	SRR5788075	24460059	SRZ187676	metaSPAdes v3.9.0
S0492	GEOTRACES	GA10	D357	3 reoccupied	2010-11-12T07:08:00	61.5	ocean_biome	ocean	water	Atlantic Ocean	36.4495 S 13.21958 E	237882	PRJNA385854	SRP110813	SAMN07136954	SRR5788076	30260345	SRZ187677	metaSPAdes v3.9.0
S0493	GEOTRACES	GA10	D357	3 reoccupied	2010-11-12T07:08:00	80.9	ocean_biome	ocean	water	Atlantic Ocean	36.4495 S 13.21958 E	237881	PRJNA385854	SRP110813	SAMN07136955	SRR5788073	26635656	SRZ187678	metaSPAdes v3.9.0
S0494	GEOTRACES	GA10	D357	3 reoccupied	2010-11-12T07:08:00	100.9	ocean_biome	ocean	water	Atlantic Ocean	36.4495 S 13.21958 E	237880	PRJNA385854	SRP110813	SAMN07136956	SRR5788074	23191986	SRZ187679	metaSPAdes v3.9.0
S0495	GEOTRACES	GA10	D357	11	2010-11-15T02:37:00	6.1	ocean_biome	ocean	water	Atlantic Ocean	39.25553 S 7.72506 E	237937	PRJNA385854	SRP110813	SAMN07136957	SRR5788079	27445687	SRZ187680	metaSPAdes v3.10.1
S0496	GEOTRACES	GA10	D357	11	2010-11-15T02:37:00	21.4	ocean_biome	ocean	water	Atlantic Ocean	39.25553 S 7.72506 E	237936	PRJNA385854	SRP110813	SAMN07136958	SRR5788080	27301346	SRZ187681	metaSPAdes v3.10.1
S0497	GEOTRACES	GA10	D357	11	2010-11-15T02:37:00	51.2	ocean_biome	ocean	water	Atlantic Ocean	39.25553 S 7.72506 E	237934	PRJNA385854	SRP110813	SAMN07136959	SRR5788077	18333948	SRZ187682	metaSPAdes v3.10.1
S0498	GEOTRACES	GA10	D357	11	2010-11-15T02:37:00	71.3	ocean_biome	ocean	water	Atlantic Ocean	39.25553 S 7.72506 E	237933	PRJNA385854	SRP110813	SAMN07136960	SRR5788078	25004485	SRZ187683	metaSPAdes v3.10.1
S0499	GEOTRACES	GA10	D357	11	2010-11-15T02:37:00	91.5	ocean_biome	ocean	water	Atlantic Ocean	39.25553 S 7.72506 E	237932	PRJNA385854	SRP110813	SAMN07136961	SRR5788071	323810664	SRZ187684	metaSPAdes v3.10.1
S0500	GEOTRACES	GA10	D357	11	2010-11-15T02:37:00	121.1	ocean_biome	ocean	water	Atlantic Ocean	39.25553 S 7.72506 E	237930	PRJNA385854	SRP110813	SAMN07136962	SRR5788072	11099605	SRZ187685	metaSPAdes v3.9.0
S0501	HOT		HOT156	Station ALOHA	2004-02-24	5	ocean_biome	ocean	water	Pacific Ocean: North Pacific Subtropical Gyre, Station ALOHA	22.75 N 158 W	1560200314	PRJNA385855	SRP109831	SAMN07136998	SRR5720299	13333617	SRZ187079	metaSPAdes v3.9.0
S0502	HOT		HOT156	Station ALOHA	2004-02-24	100	ocean_biome	ocean	water	Pacific Ocean: North Pacific Subtropical Gyre, Station ALOHA	22.75 N 158 W	1560200308	PRJNA385855	SRP109831	SAMN07136999	SRR5720300	10540927	SRZ187080	metaSPAdes v3.9.0
S0503	HOT		HOT156	Station ALOHA	2004-02-24	175	ocean_biome	ocean	water	Pacific Ocean: North Pacific Subtropical Gyre, Station ALOHA	22.75 N 158 W	1560200304	PRJNA385855	SRP109831	SAMN07137000	SRR5720301	12242781	SRZ187081	metaSPAdes v3.9.0
S0504	HOT		HOT158	Station ALOHA	2004-04-20	25	ocean_biome	ocean	water	Pacific Ocean: North Pacific Subtropical Gyre, Station ALOHA	22.75 N 158 W	1580200313	PRJNA385855	SRP109831	SAMN07137001	SRR5720302	41507610	SRZ187082	metaSPAdes v3.10.1
S0505	HOT		HOT158	Station ALOHA	2004-04-20	125	ocean_biome	ocean	water	Pacific Ocean: North Pacific Subtropical Gyre, Station ALOHA	22.75 N 158 W	1580200306	PRJNA385855	SRP109831	SAMN07137002	SRR5720303	26554323	SRZ187083	metaSPAdes v3.9.0
S0506	HOT		HOT158	Station ALOHA	2004-04-20	175	ocean_biome	ocean	water	Pacific Ocean: North Pacific Subtropical Gyre, Station ALOHA	22.75 N 158 W	1580200304	PRJNA385855	SRP109831	SAMN07137003	SRR5720304	20096976	SRZ187084	metaSPAdes v3.9.0
S0507	HOT		HOT159	Station ALOHA	2004-05-18	5	ocean_biome	ocean	water	Pacific Ocean: North Pacific Subtropical Gyre, Station ALOHA	22.75 N 158 W	1590200314	PRJNA385855	SRP109831	SAMN07137004	SRR5720305	28122089	SRZ187085	metaSPAdes v3.9.0
S0508	HOT		HOT159	Station ALOHA	2004-05-18	115	ocean_biome	ocean	water	Pacific Ocean: North Pacific Subtropical Gyre, Station ALOHA	22.75 N 158 W	1590200307	PRJNA385855	SRP109831	SAMN07137005	SRR5720306	21950314	SRZ187086	metaSPAdes v3.9.0
S0509	HOT		HOT159	Station ALOHA	2004-05-18	175	ocean_biome	ocean	water	Pacific Ocean: North Pacific Subtropical Gyre, Station ALOHA	22.75 N 158 W	1590200304	PRJNA385855	SRP109831	SAMN07137006	SRR5720297	31649155	SRZ187087	metaSPAdes v3.9.0
S0510	HOT		HOT160	Station ALOHA	2004-06-15	5	ocean_biome	ocean	water	Pacific Ocean: North Pacific Subtropical Gyre, Station ALOHA	22.75 N 158 W	1600200414	PRJNA385855	SRP109831	SAMN07137007	SRR5720298	23448593	SRZ187088	metaSPAdes v3.9.0
S0511	HOT		HOT160	Station ALOHA	2004-06-15	125	ocean_biome	ocean	water	Pacific Ocean: North Pacific Subtropical Gyre, Station ALOHA	22.75 N 158 W	1600200406	PRJNA385855	SRP109831	SAMN07137008	SRR5720225	25291252	SRZ187089	metaSPAdes v3.9.0
S0512	HOT		HOT160	Station ALOHA	2004-06-15	175	ocean_biome	ocean	water	Pacific Ocean: North Pacific Subtropical Gyre, Station ALOHA	22.75 N 158 W	1600200404	PRJNA385855	SRP109831	SAMN07137009	SRR5720226	31199129	SRZ187090	metaSPAdes v3.9.0
S0513	HOT		HOT162	Station ALOHA	2004-08-15	5	ocean_biome	ocean	water	Pacific Ocean: North Pacific Subtropical Gyre, Station ALOHA	22.75 N 158 W	1620200414	PRJNA385855	SRP109831	SAMN07137010	SRR5720227	22370133	SRZ187091	metaSPAdes v3.9.0
S0514	HOT		HOT162	Station ALOHA	2004-08-15	100	ocean_biome	ocean	water	Pacific Ocean: North Pacific Subtropical Gyre, Station ALOHA	22.75 N 158 W	1620200408	PRJNA385855	SRP109831	SAMN07137011	SRR5720228	31512521	SRZ187092	metaSPAdes v3.9.0
S0515	HOT		HOT162	Station ALOHA	2004-08-15	175	ocean_biome	ocean	water	Pacific Ocean: North Pacific Subtropical Gyre, Station ALOHA	22.75 N 158 W	1620200404	PRJNA385855	SRP109831	SAMN07137012	SRR5720221	25125604	SRZ187093	metaSPAdes v3.9.0
S0516	HOT		HOT163	Station ALOHA	2004-09-28	5	ocean_biome	ocean	water	Pacific Ocean: North Pacific Subtropical Gyre, Station ALOHA	22.75 N 158 W	1630200414	PRJNA385855	SRP109831	SAMN07137013	SRR5720222	26379124	SRZ187094	metaSPAdes v3.9.0
S0517	HOT		HOT163	Station ALOHA	2004-09-28	115	ocean_biome	ocean	water	Pacific Ocean: North Pacific Subtropical Gyre, Station ALOHA	22.75 N 158 W	1630200407	PRJNA385855	SRP109831	SAMN07137014	SRR5720223	29188845	SRZ187095	metaSPAdes v3.9.0
S0518	HOT		HOT163	Station ALOHA	2004-09-28	175	ocean_biome	ocean	water	Pacific Ocean: North Pacific Subtropical Gyre, Station ALOHA	22.75 N 158 W	1630200404	PRJNA385855	SRP109831	SAMN07137015	SRR5720224	19503759	SRZ187096	metaSPAdes v3.9.0
S0519	HOT		HOT164	Station ALOHA	2004-10-10	5	ocean_biome	ocean	water	Pacific Ocean: North Pacific Subtropical Gyre, Station ALOHA	22.75 N 158 W	1640201117	PRJNA385855	SRP109831	SAMN07137016	SRR5720219	22377770	SRZ187097	metaSPAdes v3.9.0
S0520	HOT		HOT164	Station ALOHA	2004-10-10	115	ocean_biome	ocean	water	Pacific Ocean: North Pacific Subtropical Gyre, Station ALOHA	22.75 N 158 W	1640201108	PRJNA385855	SRP109831	SAMN07137017	SRR5720220	18532257	SRZ187098	metaSPAdes v3.9.0
S0521	HOT		HOT164	Station ALOHA	2004-10-10	175	ocean_biome	ocean	water	Pacific Ocean: North Pacific Subtropical Gyre, Station ALOHA	22.75 N 158 W	1640201104	PRJNA385855	SRP109831	SAMN07137018	SRR5720242	25512857	SRZ187099	metaSPAdes v3.9.0
S0522	HOT		HOT165	Station ALOHA	2004-11-10	5	ocean_biome	ocean	water	Pacific Ocean: North Pacific Subtropical Gyre, Station ALOHA	22.75 N 158 W	1650200419	PRJNA385855	SRP109831	SAMN07137019	SRR5720241	32721051	SRZ187100	metaSPAdes v3.9.0
S0523	HOT		HOT165	Station ALOHA	2004-11-10	100	ocean_biome	ocean	water	Pacific Ocean: North Pacific Subtropical Gyre, Station ALOHA	22.75 N 158 W	1650200409	PRJNA385855	SRP109831	SAMN07137020	SRR5720244	39013537	SRZ187101	metaSPAdes v3.9.0
S0524	HOT		HOT165	Station ALOHA	2004-11-10	175	ocean_biome	ocean	water	Pacific Ocean: North Pacific Subtropical Gyre, Station ALOHA	22.75 N 158 W	1650200404	PRJNA385855	SRP109831	SAMN07137021	SRR5720243	40531155	SRZ187102	metaSPAdes v3.9.0
S0525	HOT		HOT166	Station ALOHA	2004-12-14	5	ocean_biome	ocean	water	Pacific Ocean: North Pacific Subtropical Gyre, Station ALOHA	22.75 N 158 W	1660200514	PRJNA385855	SRP109831	SAMN07137022	SRR5720246	33468362	SRZ187103	metaSPAdes v3.9.0
S0526	HOT		HOT166	Station ALOHA	2004-12-14	100	ocean_biome	ocean	water	Pacific Ocean: North Pacific Subtropical Gyre, Station ALOHA	22.75 N 158 W	1660200508	PRJNA385855	SRP109831	SAMN07137023	SRR5720245	31170529	SRZ187104	metaSPAdes v3.9.0
S0527	HOT		HOT166	Station ALOHA	2004-12-14	175	ocean_biome	ocean	water	Pacific Ocean: North Pacific Subtropical Gyre, Station ALOHA	22.75 N 158 W	1660200504	PRJNA385855	SRP109831	SAMN07137024	SRR5720248	23767421	SRZ187105	metaSPAdes v3.9.0
S0528	HOT		HOT144	Station ALOHA	2003-01-17	5	ocean_biome	ocean	water	Pacific Ocean: North Pacific Subtropical Gyre, Station ALOHA	22.75 N 158 W	1440200914	PRJNA385855	SRP109831	SAMN07137025	SRR5720247	26441873	SRZ187106	metaSPAdes v3.9.0
S0529	HOT		HOT144	Station ALOHA	2003-01-17	125	ocean_biome	ocean	water	Pacific Ocean: North Pacific Subtropical Gyre, Station ALOHA	22.75 N 158 W	1440200906	PRJNA385855	SRP109831	SAMN07137026	SRR5720240	26191174	SRZ187107	metaSPAdes v3.9.0
S0530	HOT		HOT144	Station ALOHA	2003-01-17	175	ocean_biome	ocean	water	Pacific Ocean: North Pacific Subtropical Gyre, Station ALOHA	22.75 N 158 W	1440200904	PRJNA385855	SRP109831	SAMN07137027	SRR5720239	34151795	SRZ187108	metaSPAdes v3.9.0
S0531	HOT		HOT145	Station ALOHA	2003-02-25	5	ocean_biome	ocean	water	Pacific Ocean: North Pacific Subtropical Gyre, Station ALOHA	22.75 N 158 W	1450200318	PRJNA385855	SRP109831	SAMN07137028	SRR5720269	17902513	SRZ187109	metaSPAdes v3.9.0
S0532	HOT		HOT145	Station ALOHA	2003-02-25	115	ocean_biome	ocean	water	Pacific Ocean: North Pacific Subtropical Gyre, Station ALOHA	22.75 N 158 W	1450200308	PRJNA385855	SRP109831	SAMN07137029	SRR5720270	45464228	SRZ187110	metaSPAdes v3.9.0
S0533	HOT		HOT145	Station ALOHA	2003-02-25	175	ocean_biome	ocean	water	Pacific Ocean: North Pacific Subtropical Gyre, Station ALOHA	22.75 N 158 W	1450200305	PRJNA385855	SRP109831	SAMN07137030	SRR5720267	45638515	SRZ187111	metaSPAdes v3.9.0
S0534	HOT		HOT146	Station ALOHA	2003-03-28	5	ocean_biome	ocean	water	Pacific Ocean: North Pacific Subtropical Gyre, Station ALOHA	22.75 N 158 W	1460200314	PRJNA385855	SRP109831	SAMN07137031	SRR5720268	17826774	SRZ187112	metaSPAdes v3.9.0
S0535	HOT		HOT146	Station ALOHA	2003-03-28	125	ocean_biome	ocean	water	Pacific Ocean: North Pacific Subtropical Gyre, Station ALOHA	22.75 N 158 W	1460200307	PRJNA385855	SRP109831	SAMN07137032	SRR5720273	31242438	SRZ187113	metaSPAdes v3.9.0
S0536	HOT		HOT146	Station ALOHA	2003-03-28	175	ocean_biome	ocean	water	Pacific Ocean: North Pacific Subtropical Gyre, Station ALOHA	22.75 N 158 W	1460200304	PRJNA385855	SRP109831	SAMN07137033	SRR5720274	35956418	SRZ187114	metaSPAdes v3.9.0
S0537	HOT		HOT147	Station ALOHA	2003-04-23	5	ocean_biome	ocean	water	Pacific Ocean: North Pacific Subtropical Gyre, Station ALOHA	22.75 N 158 W	1470200314	PRJNA385855	SRP109831	SAMN07137034	SRR5720271	25313647	SRZ187115	metaSPAdes v3.9.0
S0538	HOT		HOT147	Station ALOHA	2003-04-23	125	ocean_biome	ocean	water	Pacific Ocean: North Pacific Subtropical Gyre, Station ALOHA	22.75 N 158 W	1470200306	PRJNA385855	SRP109831	SAMN07137035	SRR5720272	31854278	SRZ187116	metaSPAdes v3.9.0
S0539	HOT		HOT147	Station ALOHA	2003-04-23	175	ocean_biome	ocean	water	Pacific Ocean: North Pacific Subtropical Gyre, Station ALOHA	22.75 N 158 W	1470200304	PRJNA385855	SRP109831	SAMN07137036	SRR5720265	38669468	SRZ187117	metaSPAdes v3.9.0
S0540	HOT		HOT148	Station ALOHA	2003-05-20	5	ocean_biome	ocean	water	Pacific Ocean: North Pacific Subtropical Gyre, Station ALOHA	22.75 N 158 W	1480200316	PRJNA385855	SRP109831	SAMN07137037	SRR5720266	23802307	SRZ187118	metaSPAdes v3.9.0
S0541	HOT		HOT148	Station ALOHA	2003-05-20	115	ocean_biome	ocean	water	Pacific Ocean: North Pacific Subtropical Gyre, Station ALOHA	22.75 N 158 W	1480200307	PRJNA385855	SRP109831	SAMN07137038	SRR5720296	25392429	SRZ187119	metaSPAdes v3.9.0
S0542	HOT		HOT148	Station ALOHA	2003-05-20	175	ocean_biome	ocean	water	Pacific Ocean: North Pacific Subtropical Gyre, Station ALOHA	22.75 N 158 W	1480200304	PRJNA385855	SRP109831	SAMN07137039	SRR5720295	15276603	SRZ187120	metaSPAdes v3.9.0
S0548	HOT		HOT150	Station ALOHA	2003-07-19	175	ocean_biome	ocean	water	Pacific Ocean: North Pacific Subtropical Gyre, Station ALOHA	22.75 N 158 W	1500200304	PRJNA385855	SRP109831	SAMN07137045	SRR5720289	15341429	SRZ187126	metaSPAdes v3.9.0
S0549	HOT		HOT151	Station ALOHA	2003-08-20	5	ocean_biome	ocean	water	Pacific Ocean: North Pacific Subtropical Gyre, Station ALOHA	22.75 N 158 W	1510200316	PRJNA385855	SRP109831	SAMN07137046	SRR5720288	12150878	SRZ187127	metaSPAdes v3.9.0
S0550	HOT		HOT151	Station ALOHA	2003-08-20	115	ocean_biome	ocean	water	Pacific Ocean: North Pacific Subtropical Gyre, Station ALOHA	22.75 N 158 W	1510200307	PRJNA385855	SRP109831	SAMN07137047	SRR5720287	13236718	SRZ187128	metaSPAdes v3.9.0
S0551	HOT		HOT151	Station ALOHA	2003-08-20	175	ocean_biome	ocean	water	Pacific Ocean: North Pacific Subtropical Gyre, Station ALOHA	22.75 N 158 W	1510200304	PRJNA385855	SRP109831	SAMN07137048	SRR5720310	24098907	SRZ187129	metaSPAdes v3.9.0
S0552	HOT		HOT153	Station ALOHA	2003-11-09	5	ocean_biome	ocean	water	Pacific Ocean: North Pacific Subtropical Gyre, Station ALOHA	22.75 N 158 W	1530200314	PRJNA385855	SRP109831	SAMN07137049	SRR5720311	35891185	SRZ187130	metaSPAdes v3.10.1
S0553	HOT		HOT153	Station ALOHA	2003-11-09	100	ocean_biome	ocean	water	Pacific Ocean: North Pacific Subtropical Gyre, Station ALOHA	22.75 N 158 W	1530200308	PRJNA385855	SRP109831	SAMN07137050	SRR5720312	29558270	SRZ187131	metaSPAdes v3.10.1
S0554	HOT		HOT153	Station ALOHA	2003-11-09	175	ocean_biome	ocean	water	Pacific Ocean: North Pacific Subtropical Gyre, Station ALOHA	22.75 N 158 W	1530200304	PRJNA385855	SRP109831	SAMN07137051	SRR5720313	18120933	SRZ187132	metaSPAdes v3.10.1
S0555	HOT		HOT154	Station ALOHA	2003-12-20	5	ocean_biome	ocean	water	Pacific Ocean: North Pacific Subtropical Gyre, Station ALOHA	22.75 N 158 W	1540201018	PRJNA385855	SRP109831	SAMN07137052	SRR5720314	15653365	SRZ187133	metaSPAdes v3.10.1
S0556	HOT		HOT154	Station ALOHA	2003-12-20	85	ocean_biome	ocean	water	Pacific Ocean: North Pacific Subtropical Gyre, Station ALOHA	22.75 N 158 W	1540201010	PRJNA385855	SRP109831	SAMN07137053	SRR5720315	33827557	SRZ187134	metaSPAdes v3.10.1
S0557	HOT		HOT154	Station ALOHA	2003-12-20	175	ocean_biome	ocean	water	Pacific Ocean: North Pacific Subtropical Gyre, Station ALOHA	22.75 N 158 W	1540201004	PRJNA385855	SRP109831	SAMN07137054	SRR5720316	34235985	SRZ187135	metaSPAdes v3.10.1
S0558	HOT		HOT152	Station ALOHA	2003-10-14	10	ocean_biome	ocean	water	Pacific Ocean: North Pacific Subtropical Gyre, Station ALOHA	22.75 N 158 W	1520200320	PRJNA385855	SRP109831	SAMN07137055	SRR5720317	30146983	SRZ187136	metaSPAdes v3.10.1
S0559	HOT		HOT152	Station ALOHA	2003-10-14	100	ocean_biome	ocean	water	Pacific Ocean: North Pacific Subtropical Gyre, Station ALOHA	22.75 N 158 W	1520200308	PRJNA385855	SRP109831	SAMN07137056	SRR5720308	17632241	SRZ187137	metaSPAdes v3.10.1
S0560	HOT		HOT152	Station ALOHA	2003-10-14	175	ocean_biome	ocean	water	Pacific Ocean: North Pacific Subtropical Gyre, Station ALOHA	22.75 N 158 W	1520200304	PRJNA385855	SRP109831	SAMN07137057	SRR5720309	20542115	SRZ187138	metaSPAdes v3.10.1
S0561	HOT		HOT155	Station ALOHA	2004-01-21	5	ocean_biome	ocean	water	Pacific Ocean: North Pacific Subtropical Gyre, Station ALOHA	22.75 N 158 W	1550200314	PRJNA385855	SRP109831	SAMN07137058	SRR5720329	27896151	SRZ187139	metaSPAdes v3.10.1
S0562	HOT		HOT155	Station ALOHA	2004-01-21	100	ocean_biome	ocean	water	Pacific Ocean: North Pacific Subtropical Gyre, Station ALOHA	22.75 N 158 W	1550200308	PRJNA385855	SRP109831	SAMN07137059	SRR5720328	48913098	SRZ187140	metaSPAdes v3.10.1
S0563	HOT		HOT155	Station ALOHA	2004-01-21	175	ocean_biome	ocean	water	Pacific Ocean: North Pacific Subtropical Gyre, Station ALOHA	22.75 N 158 W	1550200304	PRJNA385855	SRP109831	SAMN07137060	SRR5720331	15289992	SRZ187141	metaSPAdes v3.10.1
S0564	HOT		HOT157	Station ALOHA	2004-03-19	10	ocean_biome	ocean	water	Pacific Ocean: North Pacific Subtropical Gyre, Station ALOHA	22.75 N 158 W	1570200323	PRJNA385855	SRP109831	SAMN07137061	SRR5720330	29804104	SRZ187142	metaSPAdes v3.10.1
S0565	HOT		HOT157	Station ALOHA	2004-03-19	115	ocean_biome	ocean	water	Pacific Ocean: North Pacific Subtropical Gyre, Station ALOHA	22.75 N 158 W	1570200316	PRJNA385855	SRP109831	SAMN07137062	SRR5720325	27717415	SRZ187143	metaSPAdes v3.10.1
S0566	HOT		HOT157	Station ALOHA	2004-03-19	175	ocean_biome	ocean	water	Pacific Ocean: North Pacific Subtropical Gyre, Station ALOHA	22.75 N 158 W	1570200313	PRJNA385855	SRP109831	SAMN07137063	SRR5720324	22836170	SRZ187144	metaSPAdes v3.10.1
S0567	BATS		BATS175	BATS	2003-04-22	10	ocean_biome	ocean	water	Atlantic Ocean: Sargasso Sea, BATS	31.66 N 64.16 W	1017500401	PRJNA385855	SRP109831	SAMN07137064	SRR5720327	27210818	SRZ187145	metaSPAdes v3.10.1
S0568	BATS		BATS175	BATS	2003-04-22	80	ocean_biome	ocean	water	Atlantic Ocean: Sargasso Sea, BATS	31.66 N 64.16 W	1017500406	PRJNA385855	SRP109831	SAMN07137065	SRR5720326	37828867	SRZ187146	metaSPAdes v3.10.1
S0569	BATS		BATS175	BATS	2003-04-22	180	ocean_biome	ocean	water	Atlantic Ocean: Sargasso Sea, BATS	31.66 N 64.16 W	1017500411	PRJNA385855	SRP109831	SAMN07137066	SRR5720333	27382064	SRZ187147	metaSPAdes v3.10.1
S0570	BATS		BATS181	BATS	2003-10-07	1	ocean_biome	ocean	water	Atlantic Ocean: Sargasso Sea, BATS	31.66 N 64.16 W	1018100401	PRJNA385855	SRP109831	SAMN07137067	SRR5720332	22197442	SRZ187148	metaSPAdes v3.10.1
S0571	BATS		BATS181	BATS	2003-10-07	80	ocean_biome	ocean	water	Atlantic Ocean: Sargasso Sea, BATS	31.66 N 64.16 W	1018100406	PRJNA385855	SRP109831	SAMN07137068	SRR5720340	22703083	SRZ187149	metaSPAdes v3.10.1
S0572	BATS		BATS181	BATS	2003-10-07	180	ocean_biome	ocean	water	Atlantic Ocean: Sargasso Sea, BATS	31.66 N 64.16 W	1018100411	PRJNA385855	SRP109831	SAMN07137069	SRR5720341	30123808	SRZ187150	metaSPAdes v3.10.1
S0573	BATS		BATS184	BATS	2004-01-27	1	ocean_biome	ocean	water	Atlantic Ocean: Sargasso Sea, BATS	31.66 N 64.16 W	1018400401	PRJNA385855	SRP109831	SAMN07137070	SRR5720338	36979656	SRZ187151	metaSPAdes v3.10.1
S0574	BATS		BATS184	BATS	2004-01-27	40	ocean_biome	ocean	water	Atlantic Ocean: Sargasso Sea, BATS	31.66 N 64.16 W	1018400408	PRJNA385855	SRP109831	SAMN07137071	SRR5720339	39799254	SRZ187152	metaSPAdes v3.10.1
S0575	BATS		BATS184	BATS	2004-01-27	180	ocean_biome	ocean	water	Atlantic Ocean: Sargasso Sea, BATS	31.66 N 64.16 W	1018400418	PRJNA385855	SRP109831	SAMN07137072	SRR5720336	35071483	SRZ187153	metaSPAdes v3.10.1
S0576	BATS		BATS186	BATS	2004-03-23	1	ocean_biome	ocean	water	Atlantic Ocean: Sargasso Sea, BATS	31.66 N 64.16 W	1018600401	PRJNA385855	SRP109831	SAMN07137073	SRR5720337	21473448	SRZ187154	metaSPAdes v3.10.1
S0577	BATS		BATS186	BATS	2004-03-23	60	ocean_biome	ocean	water	Atlantic Ocean: Sargasso Sea, BATS	31.66 N 64.16 W	1018600411	PRJNA385855	SRP109831	SAMN07137074	SRR5720334	30926179	SRZ187155	metaSPAdes v3.10.1
S0578	BATS		BATS186	BATS	2004-03-23	180	ocean_biome	ocean	water	Atlantic Ocean: Sargasso Sea, BATS	31.66 N 64.16 W	1018600419	PRJNA385855	SRP109831	SAMN07137075	SRR5720335	34743413	SRZ187156	metaSPAdes v3.10.1
S0579	BATS		BATS195	BATS	2004-12-08	1	ocean_biome	ocean	water	Atlantic Ocean: Sargasso Sea, BATS	31.66 N 64.16 W	1019500401	PRJNA385855	SRP109831	SAMN07137076	SRR5720342	31522929	SRZ187157	metaSPAdes v3.10.1
S0580	BATS		BATS195	BATS	2004-12-08	100	ocean_biome	ocean	water	Atlantic Ocean: Sargasso Sea, BATS	31.66 N 64.16 W	1019500407	PRJNA385855	SRP109831	SAMN07137077	SRR5720343	28856326	SRZ187158	metaSPAdes v3.10.1
S0581	BATS		BATS195	BATS	2004-12-08	180	ocean_biome	ocean	water	Atlantic Ocean: Sargasso Sea, BATS	31.66 N 64.16 W	1019500411	PRJNA385855	SRP109831	SAMN07137078	SRR5720234	26654563	SRZ187159	metaSPAdes v3.10.1
S0582	BATS		BATS173	BATS	2003-02-21	1	ocean_biome	ocean	water	Atlantic Ocean: Sargasso Sea, BATS	31.66 N 64.16 W	1017300307	PRJNA385855	SRP109831	SAMN07137079	SRR5720233	20031665	SRZ187160	metaSPAdes v3.10.1
S0583	BATS		BATS173	BATS	2003-02-21	40	ocean_biome	ocean	water	Atlantic Ocean: Sargasso Sea, BATS	31.66 N 64.16 W	1017300310	PRJNA385855	SRP109831	SAMN07137080	SRR5720232	27855946	SRZ187161	metaSPAdes v3.10.1
S0584	BATS		BATS173	BATS	2003-02-21	180	ocean_biome	ocean	water	Atlantic Ocean: Sargasso Sea, BATS	31.66 N 64.16 W	1017300317	PRJNA385855	SRP109831	SAMN07137081	SRR5720231	27754160	SRZ187162	metaSPAdes v3.10.1
S0585	BATS		BATS174	BATS	2003-03-22	1	ocean_biome	ocean	water	Atlantic Ocean: Sargasso Sea, BATS	31.66 N 64.16 W	1017400411	PRJNA385855	SRP109831	SAMN07137082	SRR5720238	26097780	SRZ187163	metaSPAdes v3.10.1
S0586	BATS		BATS174	BATS	2003-03-22	80	ocean_biome	ocean	water	Atlantic Ocean: Sargasso Sea, BATS	31.66 N 64.16 W	1017400416	PRJNA385855	SRP109831	SAMN07137083	SRR5720237	27661750	SRZ187164	metaSPAdes v3.10.1
S0587	BATS		BATS174	BATS	2003-03-22	180	ocean_biome	ocean	water	Atlantic Ocean: Sargasso Sea, BATS	31.66 N 64.16 W	1017400421	PRJNA385855	SRP109831	SAMN07137084	SRR5720236	40112857	SRZ187165	metaSPAdes v3.10.1
S0588	BATS		BATS178	BATS	2003-07-15	10	ocean_biome	ocean	water	Atlantic Ocean: Sargasso Sea, BATS	31.66 N 64.16 W	1017800407	PRJNA385855	SRP109831	SAMN07137085	SRR5720235	13300975	SRZ187166	metaSPAdes v3.10.1
S0589	BATS		BATS178	BATS	2003-07-15	120	ocean_biome	ocean	water	Atlantic Ocean: Sargasso Sea, BATS	31.66 N 64.16 W	1017800414	PRJNA385855	SRP109831	SAMN07137086	SRR5720230	33880679	SRZ187167	metaSPAdes v3.10.1
S0590	BATS		BATS178	BATS	2003-07-15	180	ocean_biome	ocean	water	Atlantic Ocean: Sargasso Sea, BATS	31.66 N 64.16 W	1017800417	PRJNA385855	SRP109831	SAMN07137087	SRR5720229	33805701	SRZ187168	metaSPAdes v3.10.1
S0591	BATS		BATS179	BATS	2003-08-12	10	ocean_biome	ocean	water	Atlantic Ocean: Sargasso Sea, BATS	31.66 N 64.16 W	1017900401	PRJNA385855	SRP109831	SAMN07137088	SRR5720286	12732789	SRZ187169	metaSPAdes v3.10.1
S0597	BATS		BATS192	BATS	2004-09-14	1	ocean_biome	ocean	water	Atlantic Ocean: Sargasso Sea, BATS	31.66 N 64.16 W	1019200501	PRJNA385855	SRP109831	SAMN07137094	SRR5720251	17374067	SRZ187175	metaSPAdes v3.10.1
S0598	BATS		BATS192	BATS	2004-09-14	100	ocean_biome	ocean	water	Atlantic Ocean: Sargasso Sea, BATS	31.66 N 64.16 W	1019200507	PRJNA385855	SRP109831	SAMN07137095	SRR5720252	30163252	SRZ187176	metaSPAdes v3.10.1
S0599	BATS		BATS192	BATS	2004-09-14	180	ocean_biome	ocean	water	Atlantic Ocean: Sargasso Sea, BATS	31.66 N 64.16 W	1019200511	PRJNA385855	SRP109831	SAMN07137096	SRR5720344	23249078	SRZ187177	metaSPAdes v3.10.1
S0600	BATS		BATS193	BATS	2004-10-13	1	ocean_biome	ocean	water	Atlantic Ocean: Sargasso Sea, BATS	31.66 N 64.16 W	1019300401	PRJNA385855	SRP109831	SAMN07137097	SRR5720307	18278548	SRZ187178	metaSPAdes v3.10.1
S0601	BATS		BATS193	BATS	2004-10-13	100	ocean_biome	ocean	water	Atlantic Ocean: Sargasso Sea, BATS	31.66 N 64.16 W	1019300407	PRJNA385855	SRP109831	SAMN07137098	SRR5720280	37895083	SRZ187179	metaSPAdes v3.10.1
S0602	BATS		BATS193	BATS	2004-10-13	180	ocean_biome	ocean	water	Atlantic Ocean: Sargasso Sea, BATS	31.66 N 64.16 W	1019300411	PRJNA385855	SRP109831	SAMN07137099	SRR5720279	31018220	SRZ187180	metaSPAdes v3.10.1
S0603	BATS		BATS194	BATS	2004-11-12	1	ocean_biome	ocean	water	Atlantic Ocean: Sargasso Sea, BATS	31.66 N 64.16 W	1019400401	PRJNA385855	SRP109831	SAMN07137100	SRR5720278	24861137	SRZ187181	metaSPAdes v3.10.1
S0604	BATS		BATS194	BATS	2004-11-12	80	ocean_biome	ocean	water	Atlantic Ocean: Sargasso Sea, BATS	31.66 N 64.16 W	1019400406	PRJNA385855	SRP109831	SAMN07137101	SRR5720277	30565919	SRZ187182	metaSPAdes v3.10.1
S0605	BATS		BATS194	BATS	2004-11-12	180	ocean_biome	ocean	water	Atlantic Ocean: Sargasso Sea, BATS	31.66 N 64.16 W	1019400411	PRJNA385855	SRP109831	SAMN07137102	SRR5720284	32686035	SRZ187183	metaSPAdes v3.10.1
S0606	BATS		BATS176	BATS	2003-05-20	1	ocean_biome	ocean	water	Atlantic Ocean: Sargasso Sea, BATS	31.66 N 64.16 W	1017600501	PRJNA385855	SRP109831	SAMN07137103	SRR5720283	21770198	SRZ187184	metaSPAdes v3.10.1
S0607	BATS		BATS176	BATS	2003-05-20	100	ocean_biome	ocean	water	Atlantic Ocean: Sargasso Sea, BATS	31.66 N 64.16 W	1017600513	PRJNA385855	SRP109831	SAMN07137104	SRR5720282	31485248	SRZ187185	metaSPAdes v3.10.1
S0608	BATS		BATS176	BATS	2003-05-20	180	ocean_biome	ocean	water	Atlantic Ocean: Sargasso Sea, BATS	31.66 N 64.16 W	1017600517	PRJNA385855	SRP109831	SAMN07137105	SRR5720281	33845957	SRZ187186	metaSPAdes v3.10.1
S0609	BATS		BATS182	BATS	2003-11-04	1	ocean_biome	ocean	water	Atlantic Ocean: Sargasso Sea, BATS	31.66 N 64.16 W	1018200401	PRJNA385855	SRP109831	SAMN07137106	SRR5720276	24558490	SRZ187187	metaSPAdes v3.10.1
S0610	BATS		BATS182	BATS	2003-11-04	80	ocean_biome	ocean	water	Atlantic Ocean: Sargasso Sea, BATS	31.66 N 64.16 W	1018200406	PRJNA385855	SRP109831	SAMN07137107	SRR5720275	39505982	SRZ187188	metaSPAdes v3.10.1
S0611	BATS		BATS182	BATS	2003-11-04	180	ocean_biome	ocean	water	Atlantic Ocean: Sargasso Sea, BATS	31.66 N 64.16 W	1018200411	PRJNA385855	SRP109831	SAMN07137108	SRR5720261	29153986	SRZ187189	metaSPAdes v3.10.1
S0612	BATS		BATS183	BATS	2003-12-02	1	ocean_biome	ocean	water	Atlantic Ocean: Sargasso Sea, BATS	31.66 N 64.16 W	1018300401	PRJNA385855	SRP109831	SAMN07137109	SRR5720262	22769159	SRZ187190	metaSPAdes v3.10.1
S0613	BATS		BATS183	BATS	2003-12-02	100	ocean_biome	ocean	water	Atlantic Ocean: Sargasso Sea, BATS	31.66 N 64.16 W	1018300410	PRJNA385855	SRP109831	SAMN07137110	SRR5720263	31385119	SRZ187191	metaSPAdes v3.10.1
S0614	BATS		BATS183	BATS	2003-12-02	180	ocean_biome	ocean	water	Atlantic Ocean: Sargasso Sea, BATS	31.66 N 64.16 W	1018300414	PRJNA385855	SRP109831	SAMN07137111	SRR5720264	18682062	SRZ187192	metaSPAdes v3.10.1
S0615	BATS		BATS188	BATS	2004-05-18	10	ocean_biome	ocean	water	Atlantic Ocean: Sargasso Sea, BATS	31.66 N 64.16 W	1018800504	PRJNA385855	SRP109831	SAMN07137112	SRR5720257	14907037	SRZ187193	metaSPAdes v3.10.1
S0616	BATS		BATS188	BATS	2004-05-18	80	ocean_biome	ocean	water	Atlantic Ocean: Sargasso Sea, BATS	31.66 N 64.16 W	1018800509	PRJNA385855	SRP109831	SAMN07137113	SRR5720258	21495920	SRZ187194	metaSPAdes v3.10.1
S0617	BATS		BATS188	BATS	2004-05-18	180	ocean_biome	ocean	water	Atlantic Ocean: Sargasso Sea, BATS	31.66 N 64.16 W	1018800514	PRJNA385855	SRP109831	SAMN07137114	SRR5720259	64480797	SRZ187195	metaSPAdes v3.10.1
S0618	BATS		BATS189	BATS	2004-06-15	10	ocean_biome	ocean	water	Atlantic Ocean: Sargasso Sea, BATS	31.66 N 64.16 W	1018900301	PRJNA385855	SRP109831	SAMN07137115	SRR5720260	12568946	SRZ187196	metaSPAdes v3.10.1
S0619	BATS		BATS189	BATS	2004-06-15	100	ocean_biome	ocean	water	Atlantic Ocean: Sargasso Sea, BATS	31.66 N 64.16 W	1018900309	PRJNA385855	SRP109831	SAMN07137116	SRR5720253	20315575	SRZ187197	metaSPAdes v3.10.1
S0620	BATS		BATS189	BATS	2004-06-15	180	ocean_biome	ocean	water	Atlantic Ocean: Sargasso Sea, BATS	31.66 N 64.16 W	1018900313	PRJNA385855	SRP109831	SAMN07137117	SRR5720254	51753735	SRZ187198	metaSPAdes v3.10.1
S0621	BATS		BATS191	BATS	2004-08-17	1	ocean_biome	ocean	water	Atlantic Ocean: Sargasso Sea, BATS	31.66 N 64.16 W	1019100604	PRJNA385855	SRP109831	SAMN07137118	SRR5720321	22106643	SRZ187199	metaSPAdes v3.10.1
S0622	BATS		BATS191	BATS	2004-08-17	120	ocean_biome	ocean	water	Atlantic Ocean: Sargasso Sea, BATS	31.66 N 64.16 W	1019100611	PRJNA385855	SRP109831	SAMN07137119	SRR5720320	52129751	SRZ187200	metaSPAdes v3.10.1
S0623	BATS		BATS191	BATS	2004-08-17	180	ocean_biome	ocean	water	Atlantic Ocean: Sargasso Sea, BATS	31.66 N 64.16 W	1019100614	PRJNA385855	SRP109831	SAMN07137120	SRR5720323	37421267	SRZ187201	metaSPAdes v3.10.1
S0624	BATS		BATS185	BATS	2004-02-24	1	ocean_biome	ocean	water	Atlantic Ocean: Sargasso Sea, BATS	31.66 N 64.16 W	1018500501	PRJNA385855	SRP109831	SAMN07137121	SRR5720322	32403141	SRZ187202	metaSPAdes v3.10.1
S0625	BATS		BATS185	BATS	2004-02-24	40	ocean_biome	ocean	water	Atlantic Ocean: Sargasso Sea, BATS	31.66 N 64.16 W	1018500504	PRJNA385855	SRP109831	SAMN07137122	SRR5720319	33499189	SRZ187203	metaSPAdes v3.10.1
S0626	BATS		BATS185	BATS	2004-02-24	180	ocean_biome	ocean	water	Atlantic Ocean: Sargasso Sea, BATS	31.66 N 64.16 W	1018500511	PRJNA385855	SRP109831	SAMN07137123	SRR5720318	34361585	SRZ187204	metaSPAdes v3.10.1
S0627	HOT		HOT214	Station ALOHA	2009-08-19	5	ocean_biome	ocean	water	Pacific Ocean: North Pacific Subtropical Gyre, Station ALOHA	22.75 N 158 W	2140200308	PRJNA385855	SRP109831	SAMN08390922	SRR6507277	53778607	SRZ187076	metaSPAdes v3.10.1
S0628	HOT		HOT216	Station ALOHA	2009-11-04	100	ocean_biome	ocean	water	Pacific Ocean: North Pacific Subtropical Gyre, Station ALOHA	22.75 N 158 W	2160200304	PRJNA385855	SRP109831	SAMN08390923	SRR6507278	52916530	SRZ187075	metaSPAdes v3.10.1
S0629	BATS		BATS248	BATS	2009-07-14	10	ocean_biome	ocean	water	Atlantic Ocean: Sargasso Sea, BATS	31.66 N 64.16 W	1024800503	PRJNA385855	SRP109831	SAMN08390924	SRR6507279	50232751	SRZ187077	metaSPAdes v3.10.1
S0630	BATS		BATS252	BATS	2009-11-07	100	ocean_biome	ocean	water	Atlantic Ocean: Sargasso Sea, BATS	31.66 N 64.16 W	1025200510	PRJNA385855	SRP109831	SAMN08390925	SRR6507280	86024360	SRZ187078	metaSPAdes v3.10.1

**Table 4:** Per-library summary statistics of the metagenome datasets.

	**Minimum**	**Mean**	**Median**	**Maximum**
Paired-end reads sequenced	2,621,276	27,234,974	25,105,392	323,810,664
Total bases sequenced	7.86×10^8^	8.17×10^9^	7.53×10^9^	9.71×10^10^
