# Publisher Correction: Marine microbial metagenomes sampled across space and time

**DOI:** 10.1038/s41597-019-0054-1

**Published:** 2019-05-21

**Authors:** Steven J. Biller, Paul M. Berube, Keven Dooley, Madeline Williams, Brandon M. Satinsky, Thomas Hackl, Shane L. Hogle, Allison Coe, Kristin Bergauer, Heather A. Bouman, Thomas J. Browning, Daniele De Corte, Christel Hassler, Debbie Hulston, Jeremy E. Jacquot, Elizabeth W. Maas, Thomas Reinthaler, Eva Sintes, Taichi Yokokawa, Sallie W. Chisholm

**Affiliations:** 1 Department of Civil and Environmental Engineering, Massachusetts Institute of Technology, Cambridge, MA 02139 USA; 2 Department of Limnology and Bio-Oceanography, University of Vienna, Vienna, 1090 Austria; 3 Department of Earth Sciences, University of Oxford, Oxford, OX1 3AN UK; 4 Marine Biogeochemistry Division, GEOMAR Helmholtz Centre for Ocean Research, Kiel, 24148 Germany; 5 Research and Development Center for Marine Biosciences, Japan Agency for Marine-Earth Science and Technology, Yokosuka, 237-0061 Japan; 6 Department F.-A. Forel for Environmental and Aquatic Sciences, University of Geneva, Geneva, 1211 Switzerland; 7 National Institute of Water and Atmospheric Research, Auckland, 1010 New Zealand; 8 Department of Biological Sciences, University of Southern California, Los Angeles, CA 90089 USA; 9 Ministry for Primary Industries, Napier, 4144 New Zealand; 10 Department of Biology, Massachusetts Institute of Technology, Cambridge, MA 02139 USA; 11 Present Address: Department of Biological Sciences, Wellesley College, Wellesley, MA 02481 USA

Correction to: *Scientific Data* 10.1038/sdata.2018.176 Published online 4 September 2018

Due to a typesetting error, 25 rows were omitted from Table 3 in the original version of this Data Descriptor. These missing rows correspond to the following sample names:

S0315, S0316, S0317, S0318, S0319, S0403, S0404, S0405, S0406, S0407, S0482, S0483, S0484, S0485, S0486, S0543, S0544, S0545, S0546, S0547, S0592, S0593, S0594, S0595, S0596.

In addition, a column in the table indicating the total bases for each sample was omitted.

Table 3 has been updated to include the missing data.

